# Candidalysin activates innate epithelial immune responses via epidermal growth factor receptor

**DOI:** 10.1038/s41467-019-09915-2

**Published:** 2019-05-24

**Authors:** Jemima Ho, Xuexin Yang, Spyridoula-Angeliki Nikou, Nessim Kichik, Andrew Donkin, Nicole O. Ponde, Jonathan P. Richardson, Remi L. Gratacap, Linda S. Archambault, Christian P. Zwirner, Celia Murciano, Rhonda Henley-Smith, Selvam Thavaraj, Christopher J. Tynan, Sarah L. Gaffen, Bernhard Hube, Robert T. Wheeler, David L. Moyes, Julian R. Naglik

**Affiliations:** 1 Centre for Host-Microbiome Interactions, Faculty of Dental, Oral and Craniofacial Sciences, King’s College London, London, SE1 1UL UK; 2 Department of Life Sciences, Imperial College London, London, SW7 2AZ UK; 3 Department of Molecular & Biomedical Science, University of Maine, Orono, ME 04469 USA; 4 Centre for Oral, Clinical & Translational Science, Faculty of Dental, Oral and Craniofacial Sciences, King’s College London, London, SE1 1UL UK; 5 Central Laser Facility, Science and Technology Facilities Council, Research Complex at Harwell, Rutherford Appleton Laboratory, Didcot, OX11 0QX UK; 6 Division of Rheumatology and Clinical Immunology, University of Pittsburgh, Pittsburgh, PA 15261 USA; 7 Department of Microbial Pathogenicity Mechanisms, Leibniz Institute for Natural Product Research and Infection Biology, Hans Knöll Institute, Jena, 07745 Germany; 8 Friedrich Schiller University, Jena, 07745 Germany; 9 Graduate School of Biomedical Sciences and Engineering, University of Maine, Orono, ME 04469 USA; 10 Present Address: Protein Phosphorylation Lab, The Francis Crick Institute, London, NW1 1AT UK; 11 Present Address: Roslin Institute, University of Edinburgh, Edinburgh, EH25 9PS UK; 12 Present Address: Postharvest Technology Department, Productos Citrosol, 46721 Potries, Valencia Spain

**Keywords:** Growth factor signalling, Fungal infection, Innate immunity, Fungal host response

## Abstract

*Candida albicans* is a fungal pathobiont, able to cause epithelial cell damage and immune activation. These functions have been attributed to its secreted toxin, candidalysin, though the molecular mechanisms are poorly understood. Here, we identify epidermal growth factor receptor (EGFR) as a critical component of candidalysin-triggered immune responses. We find that both *C. albicans* and candidalysin activate human epithelial EGFR receptors and candidalysin-deficient fungal mutants poorly induce EGFR phosphorylation during murine oropharyngeal candidiasis. Furthermore, inhibition of EGFR impairs candidalysin-triggered MAPK signalling and release of neutrophil activating chemokines in vitro, and diminishes neutrophil recruitment, causing significant mortality in an EGFR-inhibited zebrafish swimbladder model of infection. Investigation into the mechanism of EGFR activation revealed the requirement of matrix metalloproteinases (MMPs), EGFR ligands and calcium. We thus identify a PAMP-independent mechanism of immune stimulation and highlight candidalysin and EGFR signalling components as potential targets for prophylactic and therapeutic intervention of mucosal candidiasis.

## Abstract (web summary)

Candida albicans is an opportunistic fungus primarily affecting immunocompromised patients. Here, the authors identify a novel mechanism of host immune stimulation and highlight candidalysin and EGFR signalling components as potential targets for prophylactic and therapeutic intervention of mucosal candidiasis.

## Introduction

C*andida albicans* is a fungus commonly present in the healthy microbiota of oral, gut and vaginal mucosae. Infections with this species can be superficial or systemic and are particularly common in immunocompromised patients, where significant morbidity and mortality is attributed^1^. A defining feature of *C. albicans* pathogenesis is the generation of filamentous hyphae. Hyphae damage mucosal epithelia and induce immune activation. In a recent study we identified candidalysin, a cytolytic peptide toxin secreted by *C. albicans* hyphae that accounts for both the epithelial damage and immunostimulatory capacity of this fungus^2^. Candidalysin is generated from its parent protein (Ece1p) via sequential enzymatic processing by fungal kexin enzymes and secreted from hyphae^3^. In oral and vaginal epithelial cells, candidalysin induces the release of lactate dehydrogenase (LDH)^2,4^, indicative of cell damage and membrane destabilisation. Candidalysin activates epithelial immunity via mitogen-activated protein kinase (MAPK) signalling molecules, namely c-Fos transcription factor and MAPK phosphatase 1 (MKP1)^2,5,6^. MAPK signalling constitutes a “danger-response” pathway^7–9^ which induces neutrophil recruitment and innate Type-17 immunity, critical for protection against mucosal candidiasis^2,4,10,11^. The mechanism of candidalysin detection by epithelial cells is unknown.

The epidermal growth factor receptor (EGFR or ErbB1/Her1) is a membrane-bound tyrosine kinase, which, together with, ErbB2 (Her2), ErbB3 (Her3) and ErbB4 (Her4), constitute the ErbB family^12^. The distribution of EGFR is diverse throughout the body and receptor activation can trigger signalling via several major pathways, including MAPK, phosphoinositide 3 kinase (PI3K), nuclear factor kappa light chain enhancer of activated B cells (NF-ĸB) and janus kinase/signal transducer and activator of transcription (JAK/STAT) pathways^13–15^. EGFR signalling can result in a number of outcomes primarily associated with growth, including cell proliferation, survival, angiogenesis, adhesion, differentiation and motility. A wide variety of viruses and bacteria are known to exploit EGFR functions for infectious and replicative benefit. However, EGFR also functions to protect the host during disease^16–19^ and can contribute to the maintenance of epithelial barriers and defences^16,20^.

We now document the EGFR as a critical component of candidalysin-triggered immune responses at the epithelium and identify a protective role for EGFR during *C. albicans* infection. We demonstrate that EGFR is activated by both *C. albicans* and candidalysin, with candidalysin-deficient fungi exhibiting impaired ability to induce EGFR phosphorylation during murine oropharyngeal candidiasis (OPC). In vitro use of EGFR kinase inhibitors (including FDA approved Gefitinib) block *C. albicans* and candidalysin-induced MAPK signalling and secretion of neutrophil activating cytokines. Accordingly, suppressed neutrophil recruitment and significant mortality in a zebrafish swimbladder model of infection is also observed following EGFR inhibition. Investigation into the mechanism of EGFR activation during infection revealed the contribution of EGFR ligands, MMPs and calcium flux as key drivers of EGFR signalling and immune stimulation.

Herein, we identify a mechanism of candidalysin-triggered EGFR activation and signalling, which initiates early epithelial cell responses during *C. albicans* infection. As such, we highlight EGFR and its related signalling molecules as potential targets for therapeutic intervention against *Candida* infection.

## Results

### EGFR is activated by *C. albicans* and candidalysin

To identify a potential host receptor for candidalysin, we first investigated the involvement of well-documented pattern recognition receptors (PRRs) and their adapters in TR146 human oral epithelial cells. siRNA-mediated knockdown of dectin-1, dectin-2, mincle, MyD88, TRIF, TRAM, MAL, TRAF6, DC-SIGN, NOD1, NOD2, TLR1 or TLR6 had no significant effect on the ability of *C. albicans* to induce c-Fos expression or MKP1 phosphorylation at 2 h post infection (p.i.) (previously determined to be the optimal time for activation^2,21^), indicating their collective lack of involvement in the candidalysin response pathway (Supplementary Fig. 1). Additionally, siRNA knockdown of TLR2 and TLR4 was previously documented to have no effect on *C.* albicans-induced c-Fos activation or MKP1 phosphorylation^21^.

**Fig. 1:**
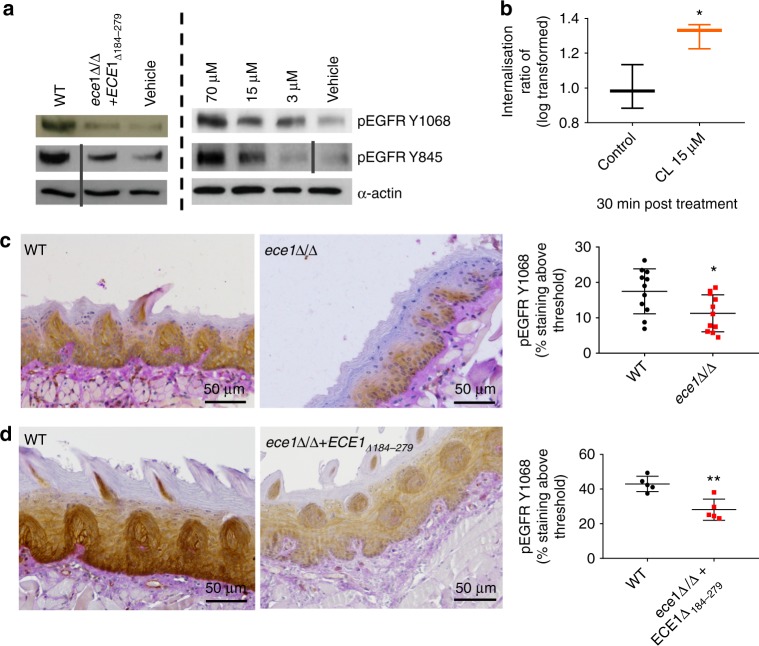
Candidalysin (CL) activates EGFR. **a** Phosphorylation: Infection of TR146 cells with WT *C. albicans* induces EGFR phosphorylation at two distinct tyrosine sites, Y1068 and Y845, while a strain lacking the candidalysin-encoding region (*ece1*Δ/Δ*+ECE1*_*Δ184–279*_) does not (left panel). Phosphorylation of EGFR at both sites was also induced following direct candidalysin treatment in a dose-dependent manner (right panel). Data are representative of three biological repeats. Protein lysates were taken at 2 h p.i. for western blot analysis. Solid vertical lines indicate omitted, extraneous portions of blot images. **b** Internalisation: At 30 min post CL treatment, EGFR is significantly internalised. Data collected from a total of 120,000 cells per group over three independent experiments. Median value indicated, error bars represent min-max data points. Values are shown as a Log transformed ratio of EGFR staining intensity inside the cell to the total cell intensity of EGFR staining. **c** Murine pEGFR staining: Immune compromised mice were infected with a *C. albicans ece1*Δ/Δ mutant (lacking *ECE1* which encodes the parent protein from which candidalysin is derived) and tongues were harvested at day 1 p.i. Paraffin-embedded, 3 mm tissue sections were prepared and stained for pEGFR (Y1068). The *ece1*Δ/Δ mutant exhibits reduced capacity to phosphorylate EGFR above a given threshold (threshold set using Image J software), when compared to WT *C. albicans* infections. Data from 9 (WT) and 11 (*ece1*Δ/Δ) individual mice obtained over two independent experiments. **d** Murine pEGFR staining: Immune competent mice were infected with the candidalysin-deficient *ece1*Δ/Δ*+ECE1*_*Δ184–279*_ mutant which also exhibited a reduced ability to phosphorylate EGFR at Y1068, when compared to WT infection. Data obtained from one experiment, 5 mice per group. Unpaired *T*-tests were used to determine statistical significance in (**b**, **c**, **d**). Error bars from (**c**, **d**) represent Standard deviation (SD). *p < 0.05, **p < 0.01

Next, we utilised previously published transcription array datasets of *C. albicans*-infected TR146 cells to identify possible pathways activated by candidalysin^22^. Ontologocial analysis of differentially expressed genes showed that EGFR binding was the third most significantly different ontology within the molecular function and biological processes categories. Further, functional annotation clustering of all ontology profiles using database for annotation, Visualisation and Integrated Discovery (DAVID), indicated the most significant cluster incorporated extensive EGFR/ErbB-associated gene sets, networks and pathways (Supplementary Fig. 2). Accordingly, we demonstrated that EGFR (but not phospho-sites of other ErbB family members (Supplementary Fig. 3)), is phosphorylated. This is observed at two distinct tyrosine sites, Y1068 and Y845, following *C. albicans* infection (Fig. 1a, left panel) or candidalysin treatment in a dose-dependent manner (Fig. 1a, right panel). Additionally, a *C. albicans* mutant strain deficient in the candidalysin-encoding region (*ece1*Δ/Δ+*ECE1*_*Δ184–279*_) was poorly able to induce EGFR phosphorylation at either tyrosine site (Fig. 1a, left panel). In light of these findings, we assessed the TR146 cell line for EGFR mutations to rule out potential genetic predisposition towards these observations. No EGFR mutations were found (Supplementary Fig. 4).

Given that EGFR is internalised upon activation, we used Imagestream X analysis, combining in-built fluorescent imaging and flow cytometry, to measure EGFR internalisation following candidalysin exposure. At 30 min post candidalysin treatment, a statistically significant increase in internalised cytoplasmic EGFR was observed (Fig. 1b). Furthermore, in a murine model of oropharyngeal candidiasis (OPC), a *C. albicans* strain lacking the candidalysin parent gene *ECE1* (*ece1*Δ/Δ) or candidalysin-encoding region (*ece1*Δ/Δ*+ECE1*_*Δ184–279*_) induced lower levels of pEGFR Y1068 in tongue tissues as compared with wild-type (WT) *C. albicans* infection (Fig. 1c, d). Collectively, these data demonstrate that candidalysin activates the EGFR.

### EGFR governs candidalysin-induced immune responses

**Fig. 2:**
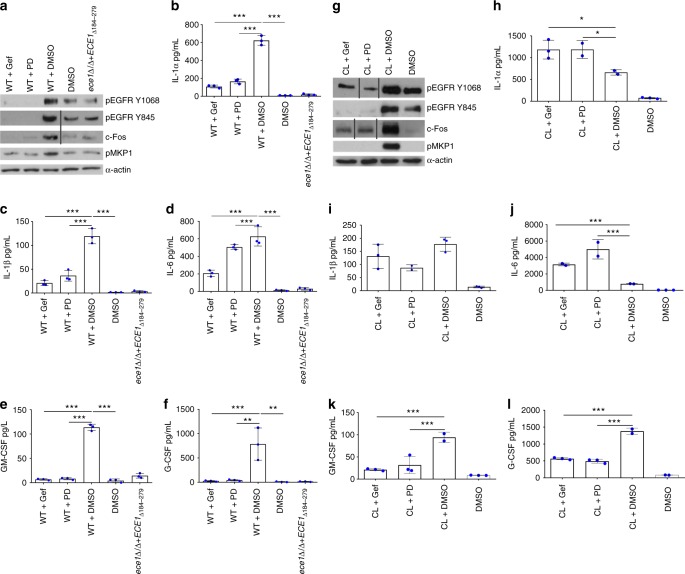
Inhibition of EGFR activity suppresses *C. albicans-* and Candidalysin (CL)-induced protein activation and expression. Use of either Gefitinib or PD153035 EGFR inhibitor suppresses WT *C. albicans*-induced (**a**–**f**) and CL-induced (**g**–**l**) pEGFR (Y1068 and Y845), c-Fos and pMKP1 (**a**, **g**), as well as secretion of GM-CSF (**e**, **k**) and G-CSF (**f**, **l**). Both EGFR inhibitors suppressed *C. albicans*-induced IL-1α, IL-1β, IL-6 (**b**–**d**) but not CL-induced IL-1α, IL-1β, IL-6 (**h**–**j**). The CL-deficient *ece1*Δ/Δ*+ECE1*_*Δ184–279*_ strain poorly induced all proteins investigated. The data are representative images (**a**, **g**) or combined averages of three independent experiments (**b**–**f**, **h**–**l**). Protein lysates taken at 2 h post infection for western blot analysis, cytokines assessed at 24 h post infection via luminex. Solid vertical lines indicate omitted, extraneous portions of blot images. Unmatched, one-way ANOVA with Bonferroni multiple comparison’s test was used to assess statistical significance. Error bars represent SD, *p < 0.05, **p < 0.01, ***p < 0.001

To establish whether EGFR drives candidalysin-induced intracellular signalling and epithelial cell immune responses, we treated TR146 epithelial cells with the EGFR inhibitors Gefitinib or PD153035, prior to *C. albicans* infection or candidalysin treatment. Both inhibitors significantly suppressed WT *C. albicans*-induced pEGFR (Y1068 and Y845), c-Fos, pMKP1 (Fig. 2a) and all cytokines investigated (IL-1α, IL-1β, IL-6, GM-CSF and G-CSF) (Fig. 2b–f). As a control, the *ece1*Δ/Δ+*ECE1*_*Δ184-279*_ candidalysin-deficient strain was used to infect non-EGFR-inhibited cells and was unable or poorly able to induce pEGFR, c-Fos, pMKP1 or cytokine release. Additionally, Gefitinib and PD153035 significantly suppressed candidalysin-induced pEGFR, c-Fos, pMKP1, GM-CSF and G-CSF (Fig. 2g, k, l), though IL-1α and IL-6 release were elevated while IL-1β release remained unchanged (Fig. 2h–j). Importantly, EGFR inhibitors did not affect candidalysin-induced LDH release (Supplementary Fig. 5a) suggesting that EGFR is not involved in the membrane-permeabilising actions of candidalysin^2^; nor does either inhibitor affect *C. albicans* hyphal growth (Supplementary Fig. 5b). The data indicate that EGFR activation is required for candidalysin-induced MAPK signalling and release of GM-CSF and G-CSF during *C. albicans* infection.

### EGFR protects against *C. albicans* infection in zebrafish

**Fig. 3:**
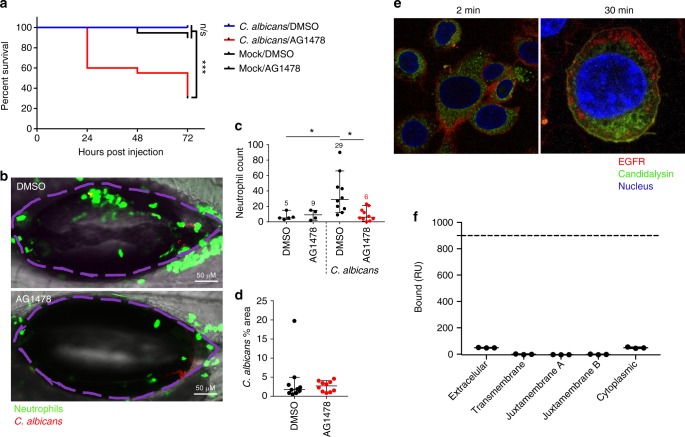
Inhibition of EGFR suppresses neutrophil recruitment and enhances mortality in a zebrafish swimbladder model of candidiasis. Candidalysin does not bind EGFR. **a** Mortality: In the presence of AG1478 EGFR inhibitor, *C.* albicans-infected fish exhibited enhanced mortality with 70% death by day 3 p.i. (red), whereas all the infected vehicle treated fish survived (blue). **b**, **c** Neutrophil recruitment: EGFR inhibition resulted in significantly fewer neutrophils (green) recruited into the infected swimbladder (outlined in purple). Numbers in graph **c** provide the median neutrophil count per group. **d** Fungal load: No significant change in fungal burden was observed in the presence of AG1478 inhibitor, as quantified by the number of red pixels, normalised to the area of the swimbladder. Log-rank with a Bonferroni correction was used for (**a**), Kruskal-Wallis with Dunn’s post-test correction for (**c**) and Mann-Whitney for (**d**). Median and interquartile range are plotted in (**c**, **d**). *p < 0.05, ***p < 0.001, n.s. *p* > 0.05. **e** Candidalysin does not co-localise with EGFR**:** Confocal images of TR146 cells show that at 2 min post exposure, fluorescently labelled candidalysin-488 (green) can be seen within the cell cytoplasm whilst EGFR (red) remains at the cell surface. At 30 min post toxin exposure, although both EGFR and candidalysin are found within cells, no co-localisation was observed, and staining patterns are distinct for each. Confocal point images have a 91.36 µm x 90.79 µm (left image) and 35.9 µm x 35.9 µm (right image) field of view. Images are representative of three individual experiments. **f** Surface plasmon resonance response of candidalysin and EGFR domains : SPR signal response for the different domains of EGFR did not reach above 50 RU when interacting with biotinylated-candidalysin (180 s injection of 500 nM of titrant at 10 µL/min flow, 25 °C). Approximate response expected for binding of extracellular or cytoplasmic domains is 900 RU (dotted line) at a Kd of 20 mM

To assess EGFR function in vivo, we used an established and tractable zebrafish swimbladder model of mucosal *C. albicans* infection that has been shown to faithfully recapitulate events in complex mammalian systems^23–26^. Immune-competent zebrafish larvae swimbladders were inoculated with *C. albicans* prior to incubation in E3-water containing AG1478 EGFR inhibitor. 70% of infected fish treated with AG1478 inhibitor died within 72 h of fungal inoculation, whereas all vehicle-treated (DMSO) controls survived infection (Fig. 3a). Additionally, AG1478-treated fish exhibited a four-fold reduction in neutrophil recruitment at sites of infection, when compared to vehicle-treated animals (Fig. 3b, c), though fungal burdens between the groups were similar (Fig. 3d). We next investigated the effect of EGFR inhibition in a murine OPC model. In immune-competent mice, however, oral administration of EGFR inhibitor (Gefitinib, PD153035, AG1478 or GW2974), induced a significant decrease in fungal burden within tongue tissues at day 1 p.i. when compared with control animals (Supplementary Fig 6). Thus, EGFR appears to promote murine oral infection but, contrastingly, offers immune protection against *C. albicans* infection in the zebrafish swimbladder model.

### Candidalysin induces EGFR ligand release

To understand how candidalysin activates EGFR, we investigated potential physical interactions between these molecules. Confocal imaging of TR146 cells after treatment with an AlexaFluor-488-labelled candidalysin and staining with Dy549-labelled EGFR affibody, revealed no co-localisation (Fig. 3e). AlexaFluor-candidalysin was observed in the cytoplasm at 2 min post-treatment while EGFR remained at the cell surface. At 30 min, both candidalysin and EGFR were found in the cytoplasm but with distinct, non-overlapping staining patterns. Additionally, surface plasmon resonance (SPR) analysis confirmed that candidalysin does not directly interact with extracellular, transmembrane or cytoplasmic EGFR receptor portions (Fig. 3f).

**Fig. 4:**
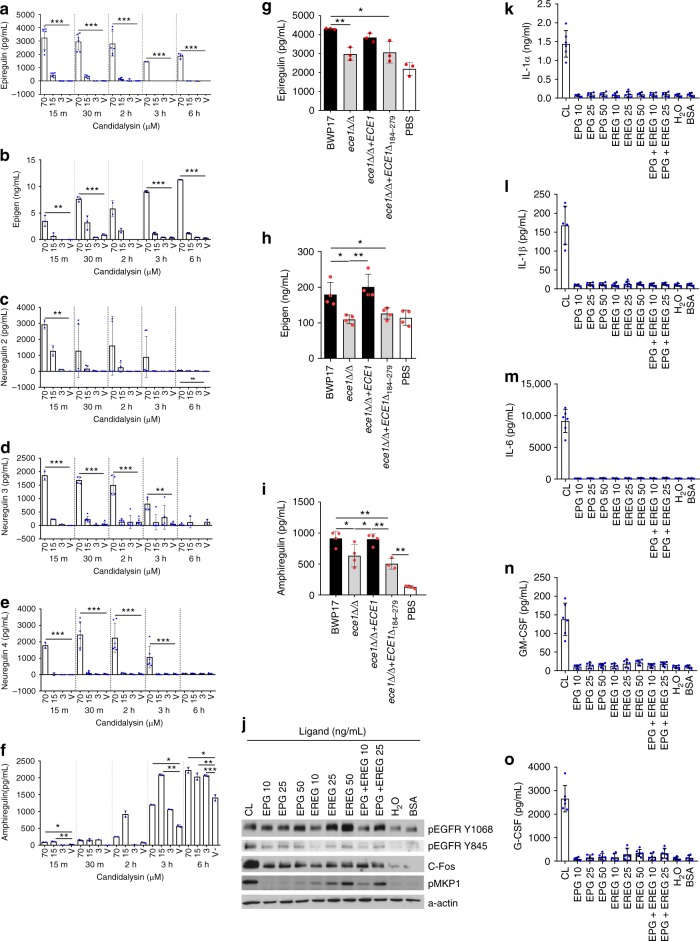
Candidalysin-induced release of EREG and EPG contribute to subsequent EGFR-mediated signalling. Release of EREG (**a**), EPG (**b**) and NRGs 2, 3 and 4 (**c**–**e**) was observed following treatment of candidalysin to TR146 cells, in a dose-dependent manner with rapid onset (within 15 min). AREG was also detected in candidalysin-treated cell supernatants, but the release was gradual and accumulated over 6 h (**f**). At 24 h p.i. epiregulin (EREG) (**g**) and epigen (EPG) (**h**) were induced by candidalysin-expressing (WT and *ece1*Δ/Δ*+ECE1* (black bars)) but not candidalysin-deficient (*ece1*Δ/Δ and *ece1*Δ/Δ*+ECE1*_*Δ184–279*_ (grey bars)) *C. albicans* strains. While amphiregulin (AREG) was induced by all fungal strains tested, diminished potency was observed by those unable to express candidalysin (**i**). Increasing concentrations of EREG, EPG or EREG + EPG, were used to stimulate TR146 oral epithelial cells. **j** Phosphorylation of EGFR (at Y1068 and Y845 sites) and MKP1 proteins occurred in a dose-dependent manner in response to EREG and EREG + EPG, but not EPG alone. Induction of c-Fos by ligand stimulation was not dose-dependent (**j**). The effects of ligand exposure are not comparable to that of 70 µM candidalysin. While a dose-dependent trend of IL-6 (**m**), GM-CSF (**n**) and G-CSF (**o**) induction is observed in response to increasing concentrations of all ligand combinations, the changes are not statistically significant. IL-1α (**k**) and IL-1β (**l**) are not induced by ligand exposure. One-way ANOVA followed by a Bonferroni multiple comparison’s test was used to calculate statistical significance between groups. Graphs are an average of 3 (**a**–**g**, **i**–**o**) or 2 (**h**) independent experiments; one-way ANOVA with Bonferroni multiple comparison’s test used to assess statistical significance between samples from the same timepoint. Error bars represent SD. *p < 0.05, **p < 0.01, ***p < 0.001

We next explored indirect mechanisms of EGFR activation by candidalysin. The ErbB family of receptors is activated by binding to one or more of their 11 endogenous ligands: epidermal growth factor (EGF), heparin-binding EGF (HB-EGF), TGF-α, amphiregulin (AREG), betacellulin (BTC), neuregulins (NRGs) 1-4, epiregulin (EREG) and epigen (EPG). ErbB family ligands are bound to the cell surface as inactive pro-ligand precursors which can undergo cleavage to release functional ectodomains that induce autocrine or paracrine signalling following ligation^27^. We found that candidalysin potently induced EREG, EPG and NRG2-4 shedding in a dose-dependent manner within 15 min post treatment (Fig. 4a–e). AREG was also released but shedding was gradual and accumulated over 6 h (Fig. 4f). Importantly, candidalysin-deficient *C. albicans* strains (*ece1*Δ/Δ and *ece1*Δ/Δ+*ECE1*_*Δ184–279*_ (grey bars)) were unable to induce EREG or EPG shedding (Fig. 4g, h), and less able to induce AREG shedding (Fig. 4i), when compared with candidalysin-expressing strains (WT and *ece1*Δ/Δ+*ECE1* (black bars)); NRG2-4 were not detected following infection with any strain. Notably, EGF, HB-EGF, TGF-α and BTC were not detectable following candidalysin treatment or *C. albicans* infection at any time point. Together, the data indicate that candidalysin induces shedding of EREG, EPG and AREG from epithelial cells during *C. albicans* infection.

### EGFR ligands partially mimic candidalysin-induced responses

We next investigated whether EGFR ligands directly mediated the candidalysin-triggered response. EREG and EPG were selected for use as exogenous stimuli based on their rapid induction by candidalysin (Fig. 4a, b) and also by their lack of induction by candidalysin-deficient *C. albicans* strains (Fig. 4g, h). We focused our investigations on immediate early responses (within 2 h), eliminating both AREG, which is not significantly released until later time points, and NRGs, which are not released at all during *C. albicans* infection, from further assessment. Dose-dependent phosphorylation of EGFR (Y1068 and Y845) and MKP1 was observed following addition of EREG or a combination of EREG and EPG (Fig. 4j), whereas EPG induced dose-dependent phosphorylation of EGFR Y1068 and MKP1 only. Induction of pEGFR Y845 or c-Fos with either ligand was not as potent as that observed with lytic doses of candidalysin (70 µM). Notably, neither EREG nor EPG were able to stimulate cytokine release (Fig. 4k–o), though a small dose-dependent trend was observed for EREG-induced IL-6, GM-CSF and G-CSF. Together, these data indicate that EREG and EPG partially mimic candidalysin-induced activation of EGFR signalling but not cytokine responses.

### MMPs are required for candidalysin-induced immune responses

**Fig. 5:**
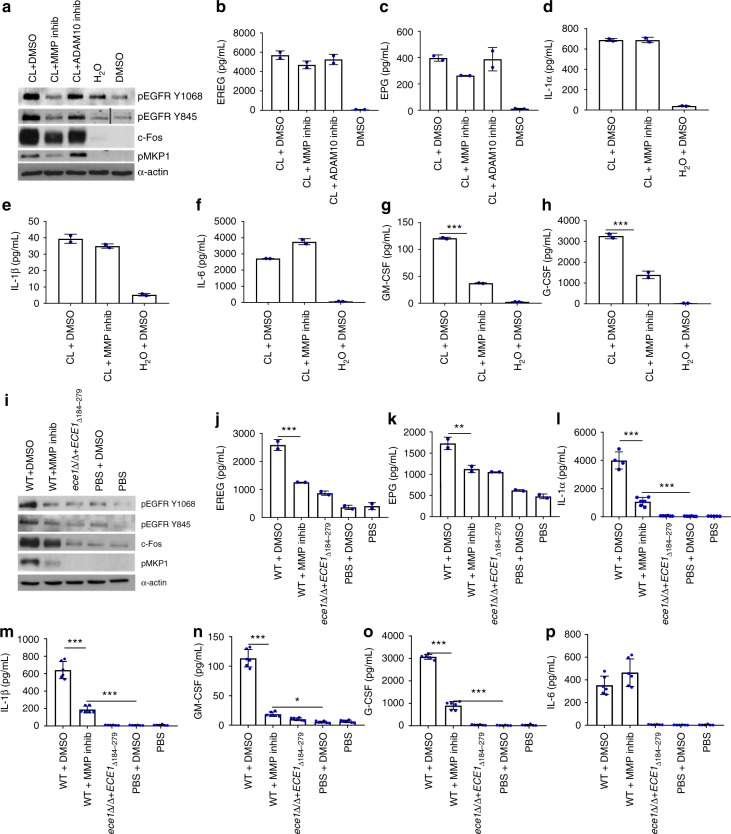
MMP inhibition suppresses the candidalysin and *C.* albicans-induced response pathway. Pre-treatment of TR146 cells with Marimastat (MMP inhibitor, 10 µM) but not GI-253023X (ADAM10 inhibitor, 5 µM), significantly suppressed candidalysin-induced expression of pEGFR (Y1068 and Y845), c-Fos and pMKP1 (**a**). MMP inhibition had no suppressive effects on candidalysin-induced EREG (**b**) EPG (**c**), IL-1α (**d**), IL-1β (**e**) or IL-6 (**f**) but did suppress GM-CSF (**g**) and G-CSF (**h**) cytokine release. In Marimastat-inhibited, WT *C.* albicans-infected cells, significant suppression of all investigated proteins (**i–o**) except IL-6 (**p**), was observed. The candidalysin-deficient *ece1*Δ/Δ*+ECE1*_*Δ184–279*_ strain also failed to induce phosphorylation of EGFR or MKP1, c-Fos expression (**i**) or our panel of cytokines (**l**–**p**). Protein lysates were taken at 2 h post infection for western blot analysis, cytokines assessed at 24 h post infection via luminex. Solid vertical lines indicate omitted, extraneous portions of blot images. Unmatched, one-way ANOVA with Bonferroni multiple comparison’s test used to assess statistical significance. All images and graphs are representative of three independent experiments. One-way ANOVA with Bonferroni multiple comparison’s test was used to assess statistical significance. Error bars represent SD. *p < 0.05, **p < 0.01, ***p < 0.001

Matrix metalloproteinases (MMPs) and A Disintegrin and Metalloproteinase domain-containing proteins (ADAMs) are two families of enzymes that cleave EGFR pro-ligands. We thus investigated their involvement in activation of EGFR-MAPK signalling in response to candidalysin. Pre-incubation of TR146 cells with the pan-MMP inhibitor Marimastat, but not the ADAM10 inhibitor GI-253023X, suppressed pEGFR (Y1068 and Y845), c-Fos and pMKP1 in candidalysin-treated cells (Fig. 5a). However, neither Marimastat nor GI-253023X had a significant effect on candidalysin-induced EREG or EPG shedding. (Fig. 5b, c). Given the lack of effect when using the ADAM10 inhibitor, further experimentation was continued with Marimastat only. Marimastat had no suppressive effects on candidalysin-induced IL-1α, IL-1β or IL-6 (Fig. 5d–f), but significantly reduced GM-CSF and G-CSF secretion (Fig. 5g, h). In response to WT *C. albicans* infection, Marimastat significantly suppressed pEGFR (Y1068 and Y845), c-Fos and pMKP1 (Fig. 5i) as well as EREG, EPG, IL-1α, IL-1β, GM-CSF and G-CSF, but not IL-6 (Fig. 5j–p). The candidalysin-deficient *C. albicans* strain, *ece1*Δ/Δ+*ECE1*_*Δ184–279*_, stimulated no or minimal responses (Fig. 5i–p), again demonstrating the requirement of candidalysin for epithelial cell activation during infection. *C. albicans* hyphal growth and candidalysin-induced LDH release were unaffected by Marimastat (Supplementary Fig. 7a, b). The data indicate that MMPs are involved in candidalysin-triggered activation of epithelial cells during *C. albicans* infection.

### Calcium chelation inhibits candidalysin-induced responses

**Fig. 6:**
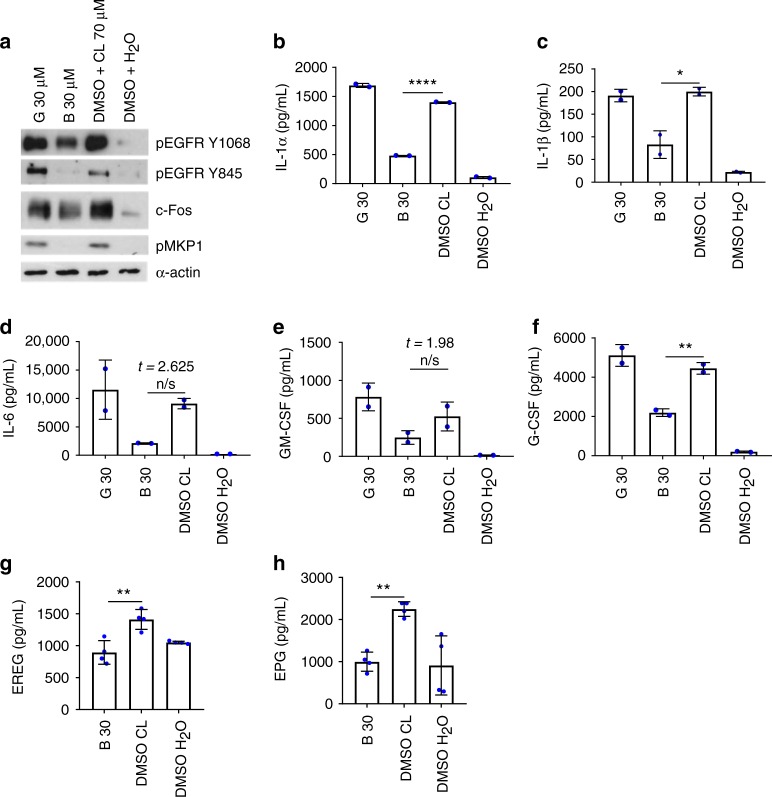
Calcium is required for candidalysin-triggered immune responses and lies upstream of EGFR. Pre-treatment of TR146 cells with a calcium chelator (Bapta-AM 30 µM) reduced levels of pEGFR Y1068 and Y845, c-Fos, pMKP1 (**a**), IL-1α (**b**), IL-1β (**c**), G-CSF (**f**), EREG (**g**) and EPG (**h**), following candidalysin exposure. This is not observed when using the potassium inhibitor glibenclamide (30 µM). IL-6 (**d**) and GM-CSF (**e**) are not significantly suppressed by either inhibitor, though a reduction is observed. Protein lysates were taken at 2 h post infection for western blot analysis, cytokines assessed at 24 h post infection via luminex. Unmatched, one-way ANOVA with Bonferroni multiple comparison’s test was used to assess statistical significance. Images and graphs are representative of 2 (**a**, **g**, **h**) or 3 (**b**–**f**) independent experiments, respectively. Error bars represent SD. *p < 0.05, **p < 0.01, ***p < 0.001, n.s. *p* > 0.05

Given that EREG and EPG did not account for candidalysin-induced cytokine release, we investigated additional mechanisms of EGFR-dependent immune activation. We have previously shown that candidalysin induces calcium influx in epithelial cells^2^ and potassium efflux in macrophages^28^. We assessed the involvement of these ions in candidalysin-triggered EGFR activation, using the potassium channel blocker glibenclamide and the intracellular calcium chelator Bapta-AM. Pre-treatment of TR146 cells with Bapta-AM, but not glibenclamide, significantly suppressed candidalysin-induced (70 µM) pEGFR (Y1068 and Y845), c-Fos and pMKP1 (Fig. 6a), IL-1α, IL-1β and G-CSF (Fig. 6b, c, f), but not IL-6 or GM-CSF secretion, though a clear trend in reduction was observed (Fig. 6d, e). Additionally, Bapta-AM was able to suppress candidalysin-induced EREG and EPG shedding (Fig. 6g, h). These observations were not made with glibenclamide. Additionally, neither inhibitor affected damage induction (Supplementary Fig. 8). These data indicate that calcium influx may contribute to the EGFR-mediated epithelial response to candidalysin.

## Discussion

Candidalysin, discovered in 2016, is the first cytolytic peptide toxin identified in any human fungal pathogen^2^. Candidalysin is immunostimulatory and critical for cytokine induction at the epithelium during *C. albicans* infection, which occurs via MAPK signalling^2,4,10,21^. However, the receptor-mediated mechanism by which activation occurs was hitherto unknown. Here, we demonstrate that EGFR activation is critical for candidalysin-induced MAPK signalling and neutrophil recruitment, which involves release of EGFR ligands and calcium influx. Thus, we identify the first PAMP-independent sensor circuit that activates immune responses against any human fungal pathogen.

EGFR is targeted by bacterial, viral and fungal species that exploit this receptor and its wide array of functions for replicative benefit^29–35^. However, beneficial host functions can also be promoted through EGFR activation^16–19^. Interestingly, there is also evidence of both mechanisms being present during infection, likely reflecting pathogen exploitation and host protective functions occurring in parallel. One example is EGFR activation by *Staphylococcus aureus*, which drives cleavage of epithelial junction proteins to facilitate barrier transmigration, but also induces protection against *S. aureus* infection^36–38^ via secretion of IL-1α and IL-1β^39^. Such opposing EGFR actions may also exist during *C. albicans* infection, where the EGFR/Her2 heterodimer has been reported as a co-receptor, binding fungal agglutinin-like sequence 3 (Als3) to aid internalisation and infection^40^. We now report an additional role for EGFR in protection against *C. albicans* pathogenicity and identify this receptor as a critical component of candidalysin-induced immune responses. This dual function of EGFR is supported by three lines of evidence from previous work showing that (i) Als3p strongly promotes fungal adhesion but does not induce MAPK (c-Fos/pMKP1) signalling or cytokine induction in oral epithelial cells^41^, (ii) an *ece1*Δ/Δ mutant is internalised but unable to induce MAPK (c-Fos/pMKP1) signalling or cytokine induction^2^, and (iii) *C. albicans* induces a strong pro-inflammatory response with accompanying protective neutrophil recruitment in a candidalysin-dependent manner in immune competent mice^4,5,10^. With these observations in mind, the contrasting data obtained from our in vivo models appear to demonstrate the two distinct EGFR functions. EGFR inhibition in mice results in reduced fungal load and no adverse effects following infection, which likely reflect the documented co-receptor functions of EGFR^40^. In zebrafish, however, EGFR activity prevents mortality and promotes neutrophil recruitment, supporting a protective and immunostimulatory role for EGFR. A suggested explanation for these distinct observations may be provided by considering the differing environments within either model. The murine oral cavity is subject to constant disruption during eating, drinking and grooming, rendering *C. albicans* heavily reliant on efficient and functional host receptors for successful infection. The swimbladder air-sac, however, provides an enclosed, undisturbed space and thus comparatively greater opportunity for fungal attachment, such that a reduction in EGFR activity may not abrogate internalisation. In this model, other receptors known to mediate *C. albicans* endocytosis, such as E-cadherin^19,20^, N-cadherin^21^ and EphA2^22^, may be sufficient in compensating in the absence of EGFR function, thus allowing for subsequent infection of tissues to provide a functional model for investigation of *C. albicans*-induced immune responses.

Interestingly, the observed reduction in neutrophil recruitment and subsequent mortality within EGFR-inhibited fish is not associated with an increase in fungal burden. Nor is it due to any toxic effects of the inhibitor, as we show that there is no mortality upon treatment of AG1478 alone. The literature surrounding neutrophil functions has historically focused on direct pathogen killing mechanisms. However, an emerging role for neutrophils in resolving the effects of infection, particularly inflammation, is being uncovered. Reports include stimulation of anti-inflammatory cytokines as well as wound healing and repair functions^42–45^. Notably, the IL-17 receptor (IL-17R), a potent regulator of neutrophil activation and antifungal immunity, has been shown to promote wound healing via EGFR recruitment^46^. Our data may thus be supportive of such functions, where a lack of inflammatory policing by neutrophils rather than overwhelming fungal burden, may explain the mortality observed in EGFR-inhibited zebrafish.

Interestingly, we show that candidalysin is able to induce both cellular damage and protective functions within the infected host. The factors that may tip the balance between disease and restoration of health in the context of *C. albicans* infection is intriguing and likely complex and multifactorial in nature. Greater understanding in this area will undoubtedly provide new avenues to improve current therapies against this pathogenic fungus.

The importance of EGFR in mediating candidalysin-induced immune responses was observed through several approaches using EGFR-inhibition, which resulted in significant loss of MAPK signalling, neutrophil activating cytokines and subsequent neutrophil recruitment into infected tissues. Additionally, we found candidalysin to be largely responsible for the shedding of several ErbB ligands during infection, all of which ligate EGFR. Interestingly, while the classical ErbB ligands such as EGF and TGF-α were not shed, candidalysin did induce shedding of EREG, EPG, AREG and NRG2,3 and 4, relatively recent ligand-family members to be identified and consequently less understood^47–49^. This is the first reported association between EGFR ligands and fungal infection, with epigen not previously being associated with any microbial infection.

While EREG and EPG contribute to candidalysin-triggered signalling, they were weak inducers of c-Fos and cytokines. Calcium flux, however, was important for initiating EGFR-mediated immune responses, with calcium chelation resulting in impaired EREG/EPG shedding, MAPK signalling and cytokine release in response to candidalysin. While calcium is not known to directly ligate EGFR, its ability to activate the EGFR has been documented, resulting in MAPK signalling and EGFR internalisation^50,51^. In light of our findings, we propose that pore-formation and calcium flux occur upstream of EGFR and are the critical first steps in epithelial activation by candidalysin.

The dependence of GM-CSF and G-CSF release on EGFR signalling was consistently observed, with significant impairment of release in the presence of EGFR or MMP inhibition. GM-CSF and G-CSF possess potent functions in neutrophil activation and chemotaxis which are necessary for successful resolution of *C. albicans* infections^52–57^. These data support our previous studies highlighting the requirement of candidalysin in neutrophil recruitment during *C. albicans* infection^2,4,10^.

In light of our findings, we propose that there exists a candidalysin-triggered pathway of EGFR activation and epithelial immune induction during invasive *C. albicans* infection. Calcium influx is induced by candidalysin-mediated membrane permeabilisation^2^ and is also a process known to induce MMP activation and expression^58–60^. We propose that cleavage of EGFR ligands by MMPs, together with increased intracellular calcium levels may function to activate EGFR, resulting in EGFR phosphorylation/internalisation and activation of MAPK signalling, driving release of neutrophil chemokines. This scenario will likely take place during hyphal growth in the invasion pocket^2,61^, where high concentrations of candidalysin may accumulate (and synergise with other fungal factors^10^), thereby reaching the threshold level required for full epithelial activation. Subsequent release of cytokines induce downstream innate immune responses, including neutrophil recruitment, critical for protection against mucosal candidiasis^2,4,10,11^.

In summary, we identify EGFR as a promoter of protective responses during *C. albicans* infection and highlight candidalysin and EGFR signalling components as potential targets for therapeutic intervention against mucosal candidiasis.

## Methods

### Cell culture

In vitro experiments were carried out using the TR146 human buccal epithelial squamous cell carcinoma cell line^62^, obtained from European Collection of Authenticated Cell Cultures (ECACC) and cultured in Dulbecco’s Modified Eagle’s Medium (DMEM, Sigma-Aldrich), supplemented with 10% foetal bovine serum (FBS) and 1% penicillin-streptomycin. Serum-free DMEM was used to replace normal growth medium 24 h before and during the experimentation process.

### Reagents

The EGFR kinase domain inhibitors were purchased from Santa Cruz (Gefitinib), Selleckchem (PD153035), Tocris Bioscience (AG1478) and Sigma (GW2974). Marimastat, GI-253023X, glibenclamide and Bapta-AM were purchased from Tocris Bioscience. Inhibitors were reconstituted in DMSO and aliquoted for appropriate freezer temperature storage. TR146 cells were incubated with inhibitors for 1 h prior to *C. albicans* infection, candidalysin exposure or mock treatment. Phospho-EGFR Tyr1068 (#3777), phospho-EGFR Tyr845 (#6963S), c-Fos (#2250S) and phospho-MKP1 (#2857S) antibodies were purchased from Cell Signalling Technology. Mouse anti-human α-actin antibody was purchased from Millipore (UK) (#MAB1501), goat anti-mouse (#115-035-062) and anti-rabbit (#111-035-144) horseradish peroxidase (HRP)-conjugated antibodies were purchased from Jackson Immunologicals (Stratech Scientific, UK). Fluorescent ErbB receptor affibodies were a kind gift from the Science and Technologies Facilities Council for use with confocal imaging work. APC anti-human EGFR antibody was purchased from BioLegend (# 352905) for use with Imagestream analyses. Biologically active EREG and EPG were purchased from Peprotech and used at 10, 25 and 50 ng/mL either individually or together at the same concentration.

### Candidalysin

Candidalysin (SIIGIIMGILGNIPQVIQIIMSIVKAFKGNK) was synthesised by Proteogenix (France) or Peptide Protein Research Ltd (UK), and reconstituted in sterile purified water to 10 mg/mL for storage, prior to further dilution for individual experiments. Alexa-488 conjugated candidalysin (C(AF488)SIIGIIMGILGNIPQVIQIIMSIVKAFKGNK), was used for confocal imaging.

### *Candida* strains

The auxotrophic BWP17+CIp30^63^ wild type (WT) *C. albicans* strain, *ece1*Δ/Δ (*ece1* null), *ece1*Δ/Δ*+ECE1* and *ece1*Δ/Δ*+ECE1*_*∆184–279*_, *C. albicans* strains were used in this study (see ref. ^2^ for full descriptions). CAF2-dTomato *C. albicans* strain was used for zebrafish experiments^24^. YPD medium (1% yeast extract, 2% peptone, 2% dextrose in water) was used to culture *C. albicans* fungi overnight in a non-airtight container, shaking at 200 rpm at 30 °C. Cultures were washed in PBS prior to dilution in the appropriate media for experimentation at the correct fungal cell density. A multiplicity of infection (MOI) of 10 was used for 2 h in vitro experiments, and MOI of 0.01 for 24 h experiments, while 1 × 10^7^ fungal cells/mL of *C. albicans* was used for murine inoculation and 15–25 fungal cells inoculated per fish swimbladder.

### ELISA

Following experimentation, culture supernatants were collected and aliquoted before storage at −80 °C for subsequent use. ELISA kits were purchased from R&D Systems (Amphiregulin, Duoset kit), ELabscience (Epiregulin, Neuregulin 2, 3, 4) and CusaBio (Epigen) and performed following the manufacturer’s instructions. Briefly, target proteins were bound to 96-well plates pre-coated with capture antibody. Biotinylated detection antibody was then applied to wells, followed by addition of a streptavidin-HRP complex. A substrate solution was then added and the absorbance of samples measured using a spectrophotometer at a wavelength of ~450 nm. Wash steps were performed three times between each stage of reagent addition.

### Western blotting

RIPA lysis buffer (50 mM Tris-HCl pH 7.4, 150 mM NaCl, 1 mM EDTA, 1% Triton X-100, 1% sodium deoxycholate, 0.1% SDS) containing protease (Sigma-Aldrich) and phosphatase (Perbio Science) inhibitors was used to lyse cells following experimentation. Lysates were left on ice for 30 min then centrifuged at 4 °C to remove debris before −80 °C storage. The concentration of protein was determined using a BCA protein quantitation kit (Perbio Science) and 10 µg of whole protein extract was separated on 12% acrylamide SDS-PAGE gels before transferring to nitrocellulose membranes (GE Healthcare). Membranes were probed for target proteins with primary (1:1000) for all proteins except c-Fos (1:3000) and secondary (1:10,000) antibodies before being developed using Immobilon chemiluminescent substrate (Millipore) and exposure to X-ray film (Fiji film). Human α-actin was used as a loading control.

### Cytokine secretion

A performance magnetic fluorokine MAP cytokine multiplex kit (Bio-techne) and a Bioplex-200 machine (Bio-Rad) were used to quantify the level of cytokine secretion in culture supernatants. Anti-IL-1α, IL-1β, IL-6, GM-CSF and G-CSF antibody beads were purchased from Bio-techne. Bioplex manager 6.1 software was used to determine analyte concentrations.

### Cell damage (LDH) assay

A Cytox 96 Non-Radioactive Cytotoxicity Assay kit (Promega) was used to measure the activity of lactate dehydrogenase (LDH) in collected culture supernatant samples. The manufacturer’s instructions were followed and recombinant porcine LDH (Sigma-Aldrich) was used to generate a standard curve. Samples were measured for LDH immediately after collection from culture plates.

### IHC staining and quantification

IHC was performed using the avidin/biotin staining method with the Vector stain elite kit (Vector labs). Formalin fixed paraffin-embedded tissue sections (5 µm) were sectioned and mounted on superfrost plus slides. Antigen retrieval was carried out with a microwave pressure cooker and citric acid buffer (pH 6.4) was pre heated to boiling. Slides were microwaved for 5 min under pressure and stained for pEGFR Y1068 with Rabbit monoclonal antibody ab40815 (Abcam) at a 1:10,000 dilution. Staining with buffer solution instead of primary antibody was used as a negative control.

Images at ×20 magnification were taken to cover the entire length of the epithelium for a given tissue section, resulting in multiple images per animal tongue. Image J software was then used to calculate the percentage staining in each image. The pixel area was first determined by drawing around the epithelium from the surface to basement membrane. Calculation of staining intensity above a given threshold was then determined using the colour-threshold tool, using set boundaries for hue (0–127) and saturation (100–255). The total area covered by pixels within this range of colour intensity was then determined and displayed as a percentage of total epithelium area. An average percentage for all images of each tongue was then used for graphical representation.

### Confocal imaging

TR146 cells were stained with DAPI and 100 nM anti-EGFR affibody-Dy549 prior to candidalysin-Alexa 488 stimulation (15 µM), and fixed with 4% formaldehyde before imaging with a Leica SP8 confocal microscope equipped with a 1.4 NA oil immersion objective. PBS was used as the imaging buffer. Leica HyD hybrid detectors operating in standard mode with a gain factor of 100 were used to acquire 1024 × 1024 images of a 116.74 × 116.74 µm field of view. DAPI fluorescence was excited at 405 nm and detected between 420 and 520 nm. Alexa 488 was excited at 488 nm and detected between 505 and 560 nm. Dy549 was excited at 561 nm and detected between 570 and 625 nm. The confocal images for each fluorescent probe were acquired sequentially. Leica Application Suite X software was used to view images.

For imaging infected zebrafish swimbladders, fish were anaesthetized with Tricaine then immobilized in 0.5% low-melting-point agarose (Lonza) in E3 containing Tricaine and arranged in a 96-well glass-bottom imaging plate (Mattek, Ashland, MA). Images were acquired on an Olympus IX-81 inverted microscope with an FV-1000 laser scanning confocal system (Olympus, Waltham, MA), using a ×20/0.7 NA or ×10/0.4 NA objective lens. EGFR and dTomato fluorescent proteins were detected by laser/optical filters for excitation/emission at 488 nm/505–525 nm and 543 nm/560–620 nm, respectively. Images were collected with Fluoview (Olympus) software.

### Imagestream

TR146 cells were treated with 15 µM candidalysin for 30 min, prior to trypsin detachment from culture plates, fixed, then stained with fluorescently labelled EGFR-APC antibody (BioLegend #352905 used at 5 µg/mL) in the presence of a permeabilising solution (BD fix/perm kit) according to the manufacturers’ instructions. Cells were washed using kit solutions and resuspended to 100 µL. An Imagestream MKII analyser (Merck) and IDEAS 6.2 software were used to measure antibody fluorescence and quantify internalisation respectively. A total of 40,000 cells per sample were acquired at ×40 magnification. Data were analysed with IDEAS 6.2 using the Internalisation feature. The inside of each cell was defined using the default mask on (BF, channel 9) eroded by 4 pixels. The internalisation feature is defined as the ratio of the intensity inside the cell to the intensity of the entire cell. The higher the score, the greater the signal intensity within the cell.

### Surface plasmon resonance (SPR)

All experiments were performed on a Biacore T100 instrument (GE Healthcare) in HBS-EP Buffer (10 mM HEPES pH 7.4, 150 mM NaCl, 3 mM EDTA and 0.005% Surfactant P20, GE Healthcare). A specific binding surface was prepared by coupling neutravidin to flow cells 2 and 3 of a CM5 sensor chip (GE Healthcare) to a level of 800 resonance units (RU) through amine coupling. N-term biotinylated candidalysin was loaded on flow cell 2 (to capture densities up to 1000 RU). A 300 s injection with PEG-biotin was performed to block remaining free neutravidin-binding sites. A biotinylated-candidalysin and PEG-biotin functionalised chip was equilibrated in HBS-EP buffer until baseline stability was observed. The different domains of EGFR (at different concentrations: 250, 500, 1000 and 2000 nM) were injected at 10 µL/min with a 100 s association phase followed by a 600 s dissociation phase. Injections over sensor surfaces with neutravidin only in flow cell 3 were performed to test unspecific binding. Both EGFR extracellular and kinase domains showed unspecific binding at injections >500 nM due to protein aggregation. Three independent runs were used for each condition where changes in the resonance signal at both, the association and dissociation steps, was followed.

### Murine oropharyngeal candidiasis model

Murine infections were performed under UK Home Office Project License PPL 70/7598 in dedicated animal facilities at King's College London. No statistical method was used to pre-determine sample size. No method of randomisation was used to allocate animals to experimental groups. Mice in the same cage were part of the same treatment. The investigators were not blinded during outcome assessment. A previously described murine model of oropharyngeal candidiasis using female Balb/c mice (22–25 g)^36^ was modified for investigating early infection events. Briefly, mice were treated subcutaneously with 3 mg/mouse of cortisone acetate (in 200 µl PBS with 0.5% Tween 80) on days −1 and 1 p. i. On day 0, mice were sedated for 75 min with an intraperitoneal injection of 110 mg/kg ketamine and 8 mg/kg xylazine, and a swab soaked in a 10^7^ cfu/ml *C. albicans* yeast culture in sterile saline was placed sub-lingually for 75 min. After 1 day, mice were sacrificed, the tongue excised and processed for immunohistochemistry. For EGFR inhibition studies, immune competent female Balb/c mice were administered Gefitinib, PD153035, AG1478 or GW2974 inhibitors at 100 mg/kg, 30 mg/kg, 20 mg/kg and 30 mg/kg respectively, each day, via an oral dose of Nutella starting at day −2 with respect to *C. albicans* infection (day 0). Appropriate volumes of inhibitor were mixed with 60 mg Nutella and placed into a feeding well for each individually-housed mouse. Mice were infected with 1 × 10^7^ cfu/mL of *Candida* as above, euthanised and tongues were excised on day 1 p.i. for fungal load assessment.

### Zebrafish swimbladder model

Cohorts of mpx:GFP zebrafish larvae were infected with 15–25 CAF2-dTomato *C. albicans* yeast at 4 days post fertilization (dpf) in the swimbladder, screened for accurate inoculum level, then incubated either with DMSO or AG1478 (3 µM) in E3 water + 0.02 mg/mL of 1-phenyl-2-thiourea (PTU) (Sigma-Aldrich) to prevent pigmentation, until imaging by confocal microscopy at 24 h. For Mock, Mock + AG1478, *C. albicans* and *C. albicans* + AG1478 groups, we used 5, 4, 10 and 10 zebrafish, respectively. The number of neutrophils in the swimbladder lumen was counted from confocal z-stacks and the median and interquartile range was plotted. *Candida* burden was quantified by the number of red fluorescent pixels in the swimbladder, normalized to the area of the swimbladder. Median and interquartile range were plotted. Confocal images were taken at 24 h p.i. Neutrophils beyond the swimbladder boarders (outlined in purple) were not included in analyses. Cohorts of wild-type AB larvae were infected and screened as described above, then monitored for survival without imaging for 4 days p.i.

### Microarray analysis

Reconstituted human oral epithelia (ROE: 5-day) created using the TR146 cell line were purchased from SkinEthic Laboratories (France) and used as previously described^21^. RNA was isolated from three independent ROE infected with *C. albicans* SC5314 or an equal volume of PBS for 6 h using the GenElute total mammalian RNA miniprep kit (Sigma, UK) and trace genomic DNA removed using the Turbo DNase-free kit (Ambion, UK). For microarray analysis, RNA was amplified using the MessageAmp Premier RNA Amplification Kit (Ambion, UK) and hybridized onto U133a 2.0 gene chips (Affymetrix, UK) according to standard protocols. Chips were scanned (Affymetrix GeneChip Scanner 3000) and assessed using the Affymetrix Command Console (AGCC) software suite. These data were statistically analysed using the Partek Genomics Suite. Genes were considered to be differentially up- or downregulated when their expression was changed by at least 2-fold with an FDR-adjusted *P*-value of less than 0.01. Gene ontology and functional annotation clustering were performed on the generated gene list using the web-based DAVID functional annotation tool https://david.ncifcrf.gov^64^.

### EGFR mutation assay

Genomic DNA was isolated from TR146 cells using the GenElute™ Mammalian Genomic DNA Miniprep Kit (Sigma) according to the manufacturer’s instructions. The DNA quantity and quality were evaluated with a NanoDrop 2000 spectrophotometer. 59 different EGFR point mutations were assessed using mutant-specific primers for Real-Time PCR (EGFR-RT52; Entrogen, Inc). DNA samples were diluted to 10 ng/μL using PCR grade RNase/DNase free water and heat-cycles were generated on a RotorGene 6000 (Qiagen). Amplification curves were evaluated according to the manufacturer’s instructions.

### Statistics

One-way analysis of variance (ANOVA) was used for all protein secretion assays (LDH, cytokines, ErbB ligands) to calculate statistical significance and corrected for multiple comparisons using the Bonferroni correction. Zebrafish data were analysed using Kruskal Wallis with Dunn’s post-test correction, Mann-Whitney and Log-rank with a Bonferroni correction. Murine in vivo data was analysed using Mann-Whitney analysis. A *p*-value of less than 0.05 was taken to be significant and represented as *, while < 0.01 = ** and < 0.001 = ***.

Ethical regulations for animal testing have been complied with and approved by either St Thomas’ Hospital and Franklin Wilkins Building Animal Welfare and Review Body (murine studies) or under the NIH Institutional Animal Care and Use Committee, protocol A2015-11-03 (zebrafish studies).

### Reporting summary

Further information on research design is available in the Nature Research Reporting Summary linked to this article.

## Supplementary information

Supplementary Information

Peer Review File

Reporting Summary

## Data Availability

Transcription array datasets of *C. albicans*-infected TR146 cells are available in the ArrayExpress (EBI suite) repository, https://www.ebi.ac.uk/arrayexpress/experiments/E-MTAB-7681. All supplementary data indicated within the manuscript, as well as uncropped and unprocessed blots can be found in Supplementary Files.
