# Supplementary material for: Candidalysin activates innate epithelial immune responses via epidermal growth factor receptor
Source: Nat Commun. 2019 May 24;10:2297. doi: 10.1038/s41467-019-09915-2 (PMC6534540; doi:10.1038/s41467-019-09915-2)
Supplement: Supplementary file 1 — Supplementary Information [file 41467_2019_9915_MOESM1_ESM.pdf]

Candidalysin activates epithelial innate immune responses via epidermal growth factor receptor

Ho et al. March 2019

Supplementary Data + Raw Blots

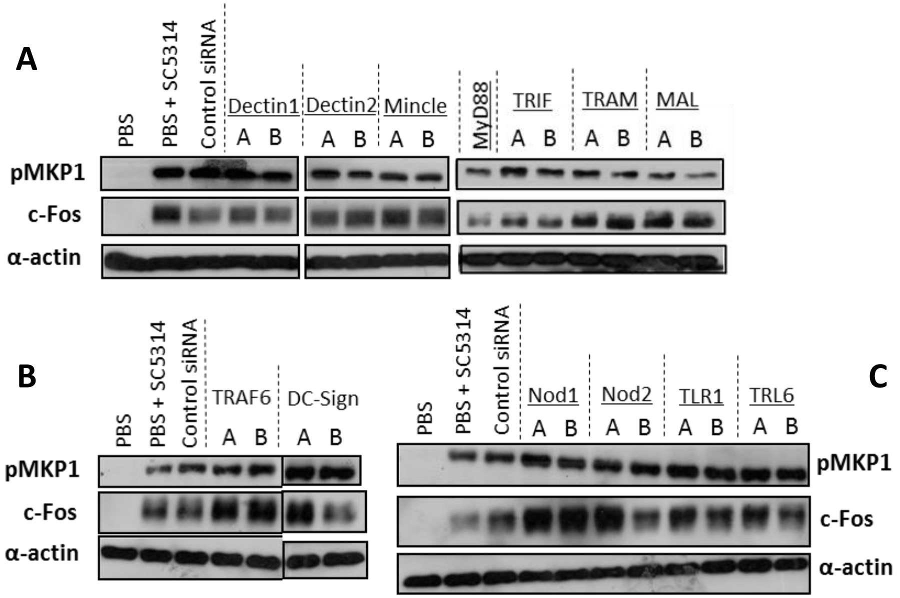

Supplementary Fig. 1: Conventional pattern recognition receptors are not involved *C. albicans*-induction of c-Fos or pMKP1. *C. albicans*-induced c-Fos expression and MKP1 phosphorylation is maintained despite transfection of siRNAs against **(A)** Dectin 1, Dectin 2, Mincle, MyD88, TRIF, TRAM, MAL, **(B)** TRAF6, DC-Sign and **(C)** Nod1, Nod2, TLR1, TLR6. Two distinct siRNAs were used for each target and are labelled A and B. Protein lysates harvested at 2 h post infection for Western blot analysis. Data suggests that these receptors do not contribute to *C. albicans* induction of c-Fos or pMKP1.

A

Category	Term	P Value
KEGG_PATHWAY	hsa04012:ErbB signaling pathway	0.042829
INTERPRO	IPR000742:Epidermal growth factor-like domain	0.02
UP_KEYWORDS	EGF-like domain	0.017028
INTERPRO	IPR013032:EGF-like, conserved site	0.012203
UP_SEQ_FEATURE	domain:EGF-like	0.004768
GOTERM_CC_DIRECT	GO:0009986~cell surface	0.003763
GOTERM_MF_DIRECT	GO:0005154~epidermal growth factor receptor binding	1.62E-05
GOTERM_BP_DIRECT	GO:0007173~epidermal growth factor receptor signaling pathway	8.19E-06
INTERPRO	IPR015497:Epidermal growth factor receptor ligand	1.71E-06
GOTERM_MF_DIRECT	GO:0008083~growth factor activity	1.42E-06
UP_KEYWORDS	Growth factor	1.18E-06
GOTERM_BP_DIRECT	GO:0008284~positive regulation of cell proliferation	4.42E-07

B

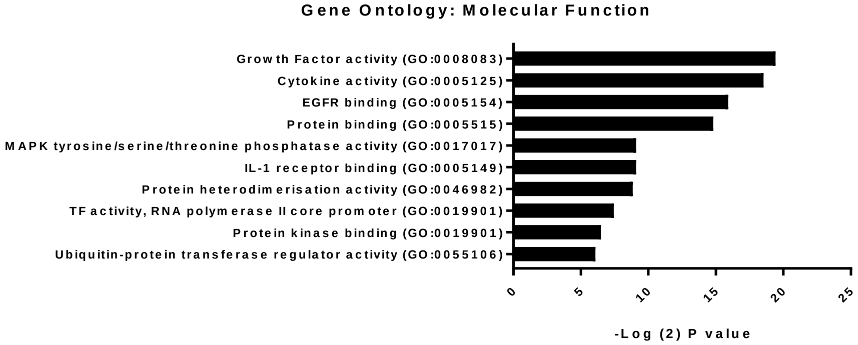

C

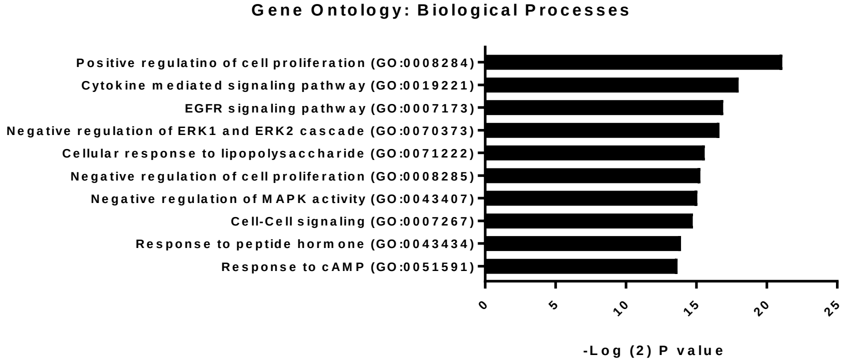

Supplementary Fig. 2: Gene array analyses highlight EGFR and related signalling molecules as one of the most differentially expressed groups. Most significant ontology groups for Biological Process (A) and Molecular Function (B) after 6 h infections of reconstituted oral epithelium with *C. albicans*. (C) Top Functional Annotation cluster of transcriptome data from reconstituted oral epithelium infected for 24 h with *C. albicans.* Data represent 3 independent experiments.

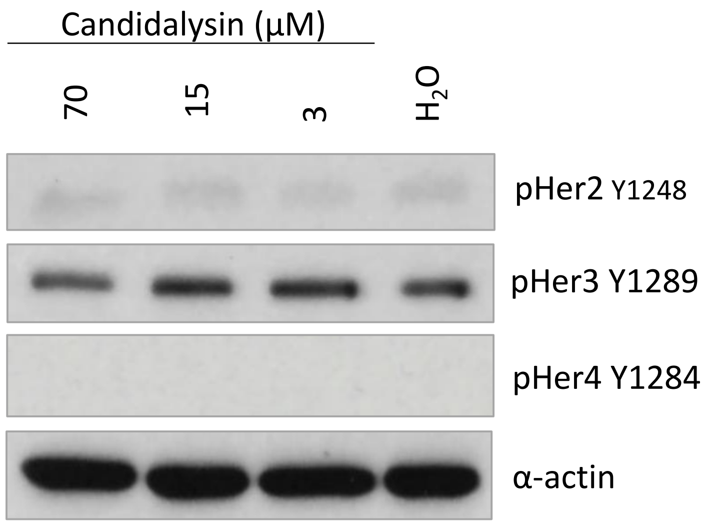

Supplementary Fig. 3: Her2, Her3 and Her4 are not phosphorylated following candidalysin exposure. Lysates from candidalysin treated cells were probed with specific phospho-antibodies to assess activation of Her family proteins. No induction of phosphorylated proteins was observed at tyrosine sites Her2 Y1248, Her3 Y1289 or Her4 Y1284. Her2 and Her4 are minimally expressed in TR146 cells. Data are representative of 3 independent experiments.

EGFR Mutation		TR146
Exon 18	G719A, G719S, G719D or G719C	X
Exon 19	Exon 19 Deletions<br>(48 different deletions)	X
Exon 20	T790M	X
	S768I	X
	Exon 20 insertions<br>(Insertion GGT or CAC)	X
	Exon 20 insertion<br>(Insertion GCCAGCGTG)	X
Exon 21	L858R	X
	L861Q	X

Supplementary Fig. 4: TR146 cells do not contain EGFR mutations. No EGFR mutations were found in the TR146 cell line. Data obtained from 2 independent experiments.

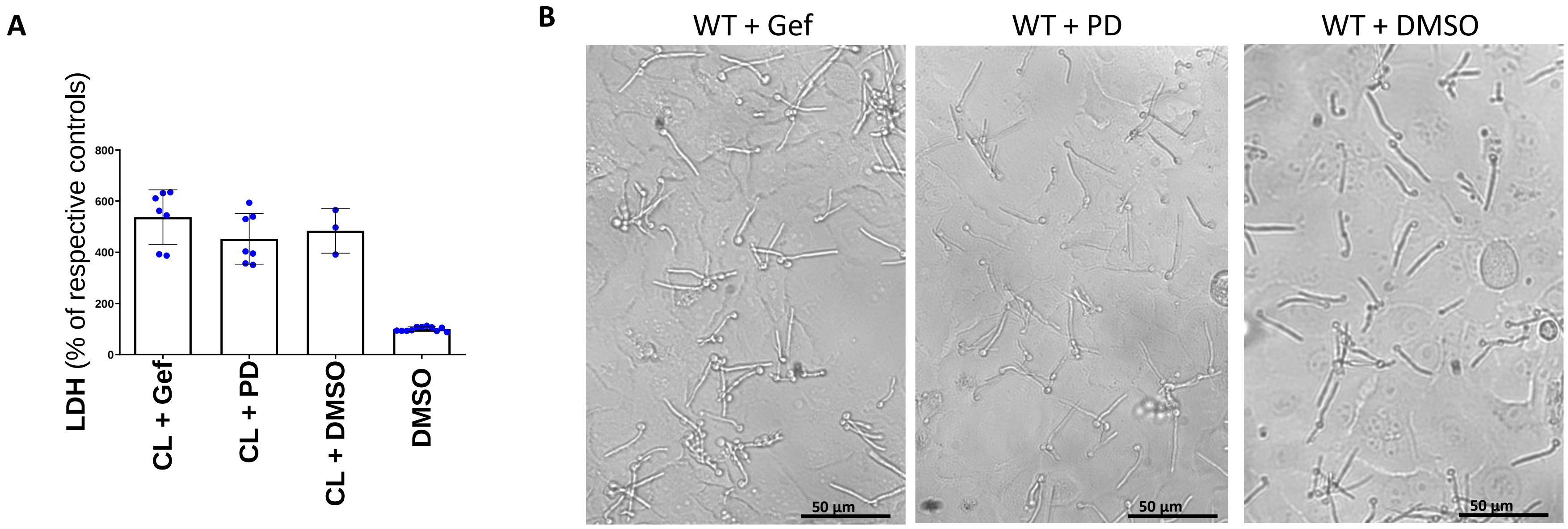

Supplementary Fig 5: EGFR inhibition does not affect candidalysin-induced damage or hypha formation.
(A) No difference in LDH release was observed between Gefitinib or PD153035 treated and controls cells, following candidalysin treatment. **(B)** No difference was observed in the formation of WT *C. albicans* hyphae at 2 h p.i. following pre-treatment with Gefitinib, PD153035 or mock inhibitor. Brightfield images taken at 20x magnification. Data are representative of 3 independent experiments. Unmatched one-way ANOVA with a Bonferroni multiple comparison’s test was used to determine statistical significance. Error bars represent SD.

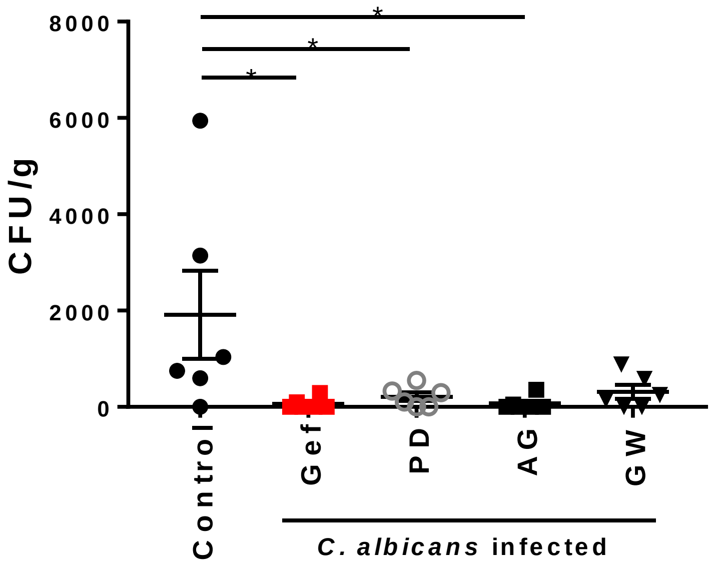

Supplementary Fig. 6: EGFR inhibition reduces fungal burdens in a murine model of OPC. Mice treated with Gefitinib, PD153035, AG1478 or GW2974 EGFR inhibitors prior to and during *C. albicans* infection, exhibited reduced counts of colony forming units (CFU) in harvested tongue tissue day1 p.i. when compared to DMSO-treated controls. Data collected from 1 experiment using 6 mice per group. Mann-Whitney test was used to determine statistical significance. Error bars represent SD. * $p<0.05$.

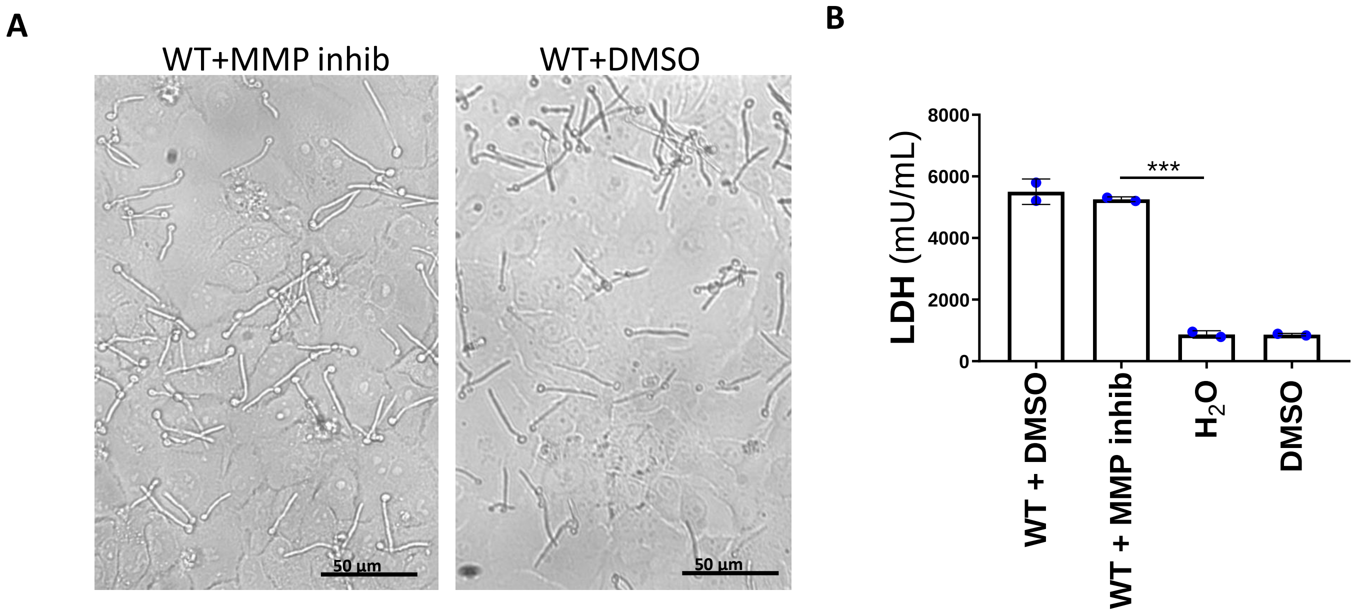

Supplementary Fig. 7: The MMP inhibitor Marimastat does not affect *C. albicans* hypha formation or damage. No difference was observed in the formation of **(A)** WT *C. albicans* hyphae or **(B)** infection-induced LDH release following pre-treatment with Marimastat or DMSO vehicle control.
Brightfield images taken at 20x magnification. Data are representative of 3 independent experiments. Unmatched one-way ANOVA with a Bonferroni multiple comparison's test was used to determine statistical significance. Error bars represent SD. *** $p<0.001$

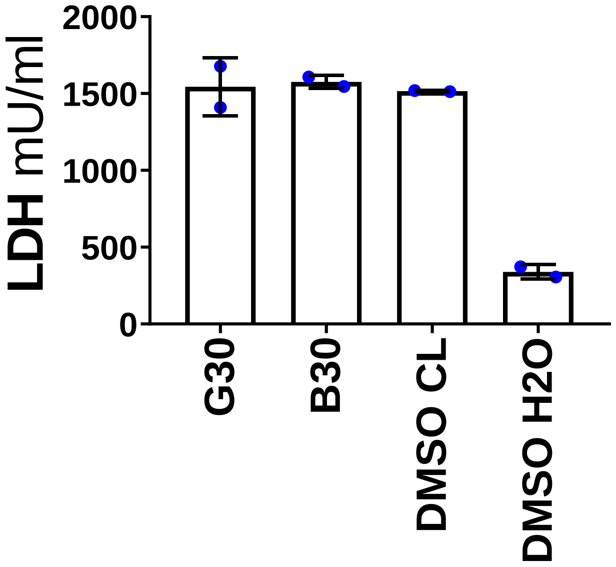

Supplementary Fig. 8: Pretreatment of TR146 cells with Glibenclamide or Bapta-AM does not affect Candidalysin-induced LDH release. Pre-treatment of TR146 epithelial cells with glibenclamide or Bapta-AM did not significantly suppress candidalysin-induced cell damage as measured by LDH release. Data are representative of 3 independent experiments. Unmatched one way ANOVA with a Bonferroni multiple comparison's test was used to determine statistical significance. Error bars represent SD.

Raw Blots

Relevant blots highlighted in Red

Fig 1A:

LHS

pEGFR Y1068

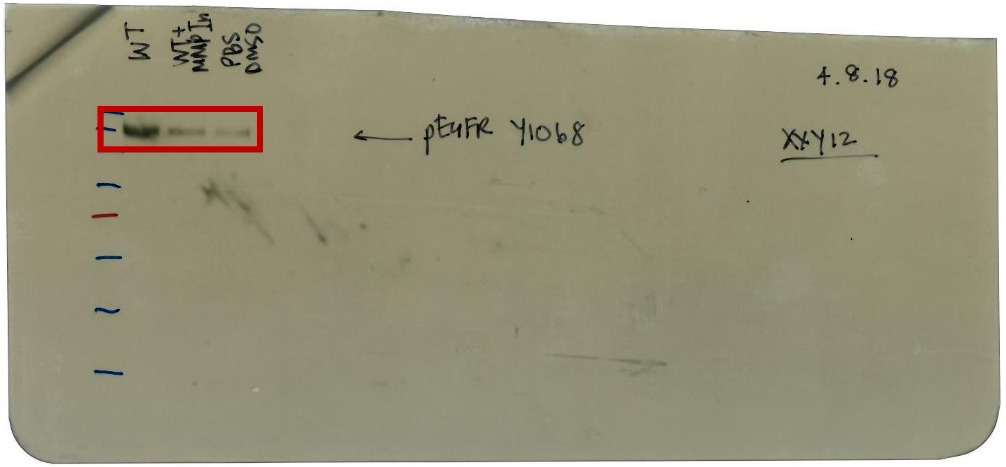

RHS

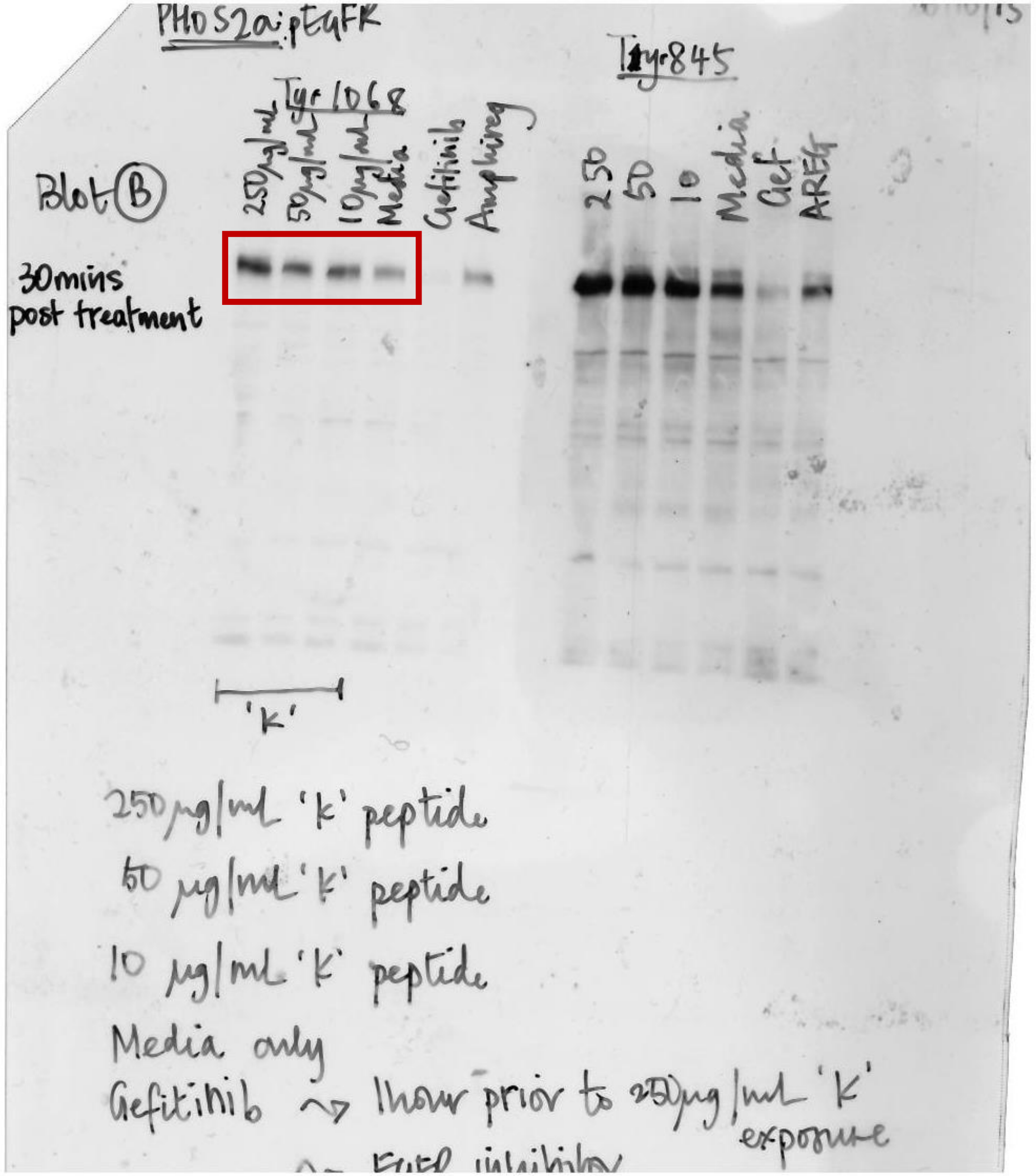

Fig 1A:

LHS

pEGFR Y845

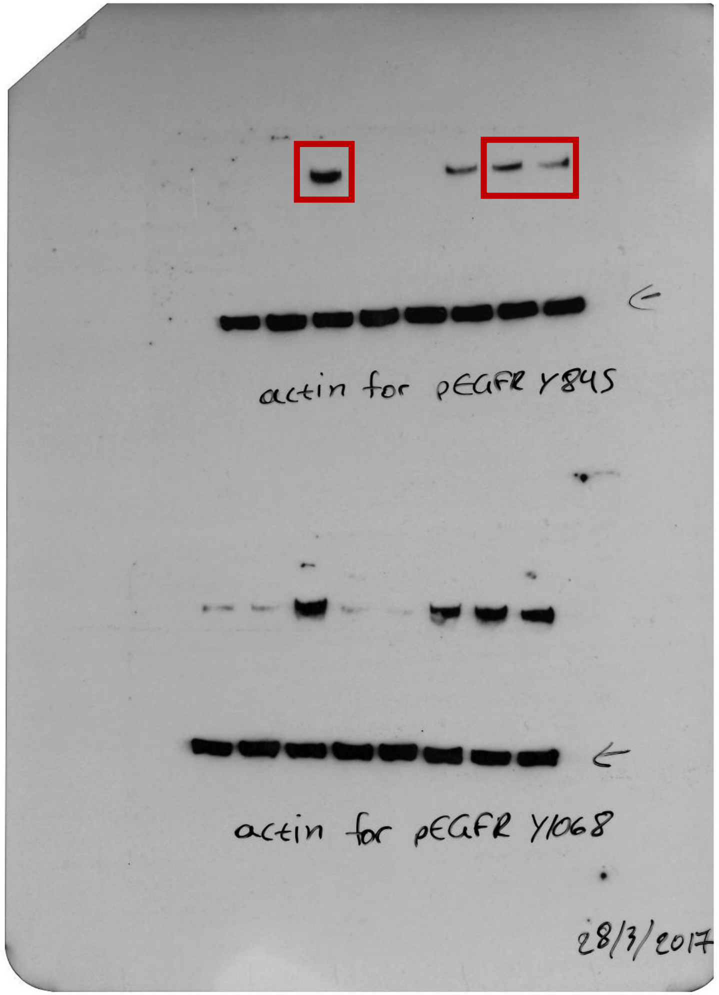

RHS

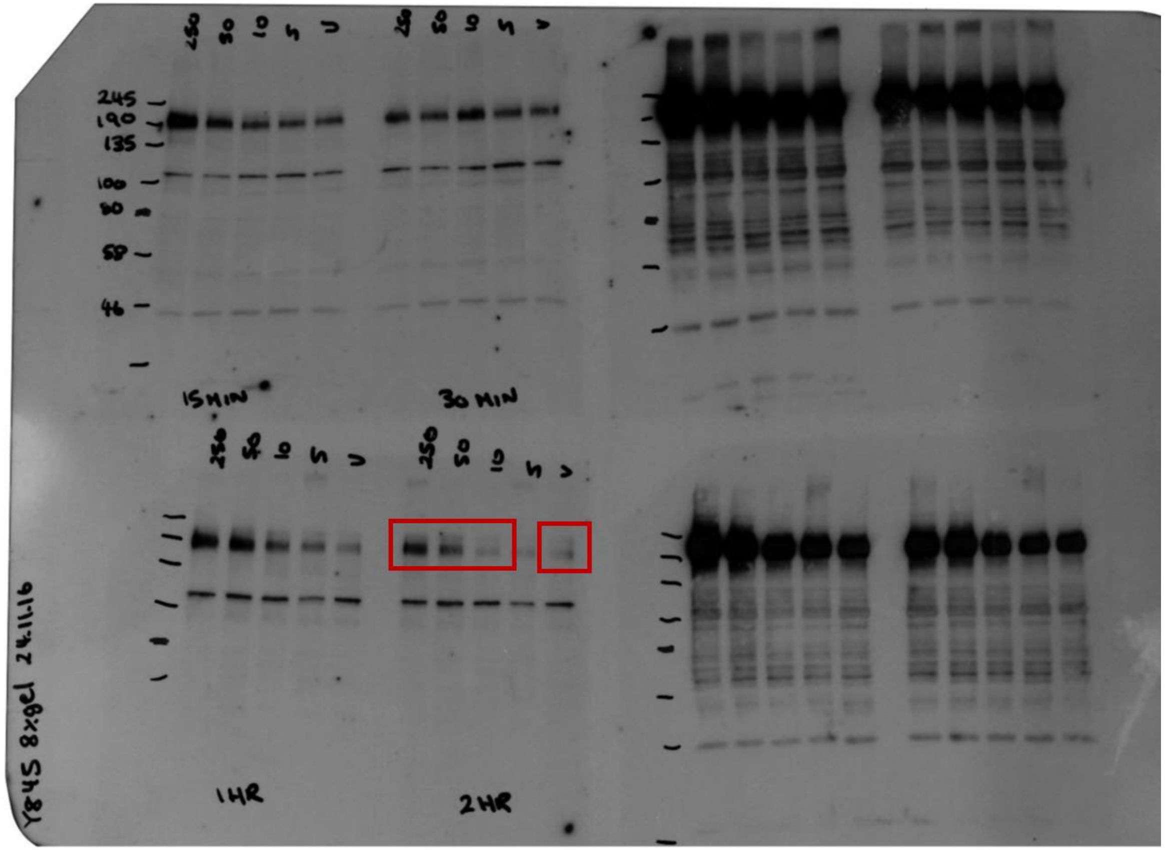

Fig 1A:

LHS

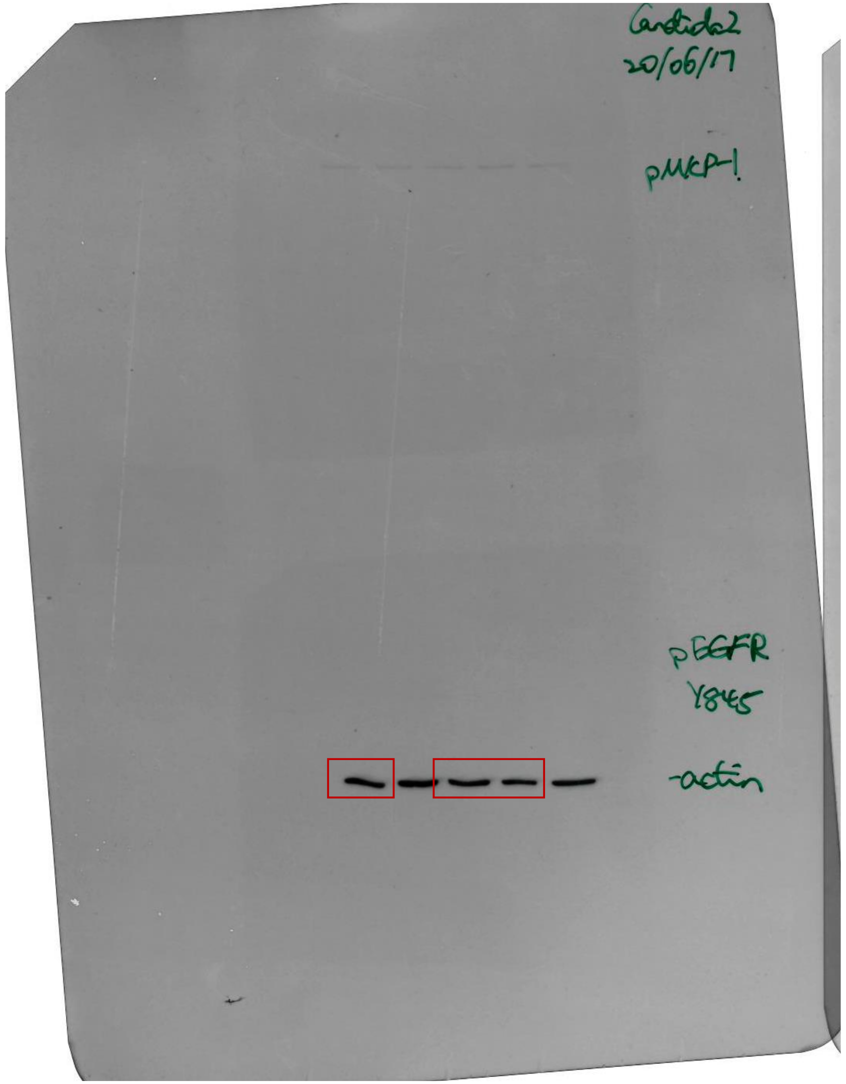

α-actin

RHS

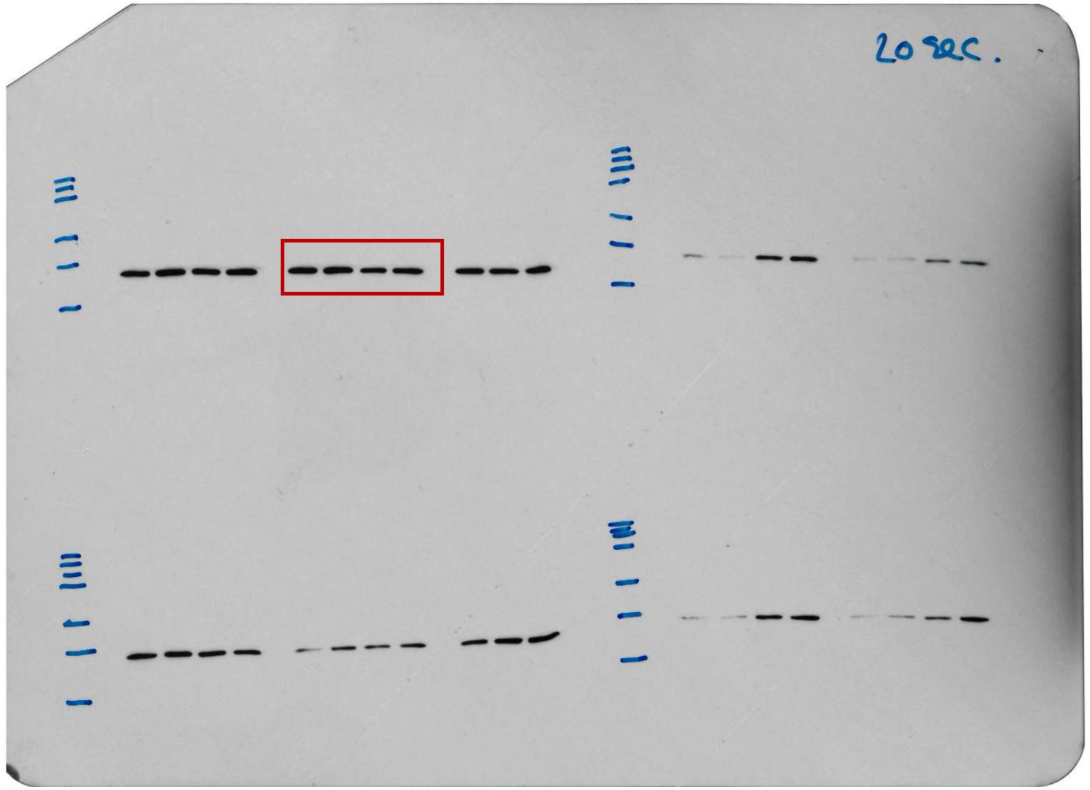

Fig 2A:

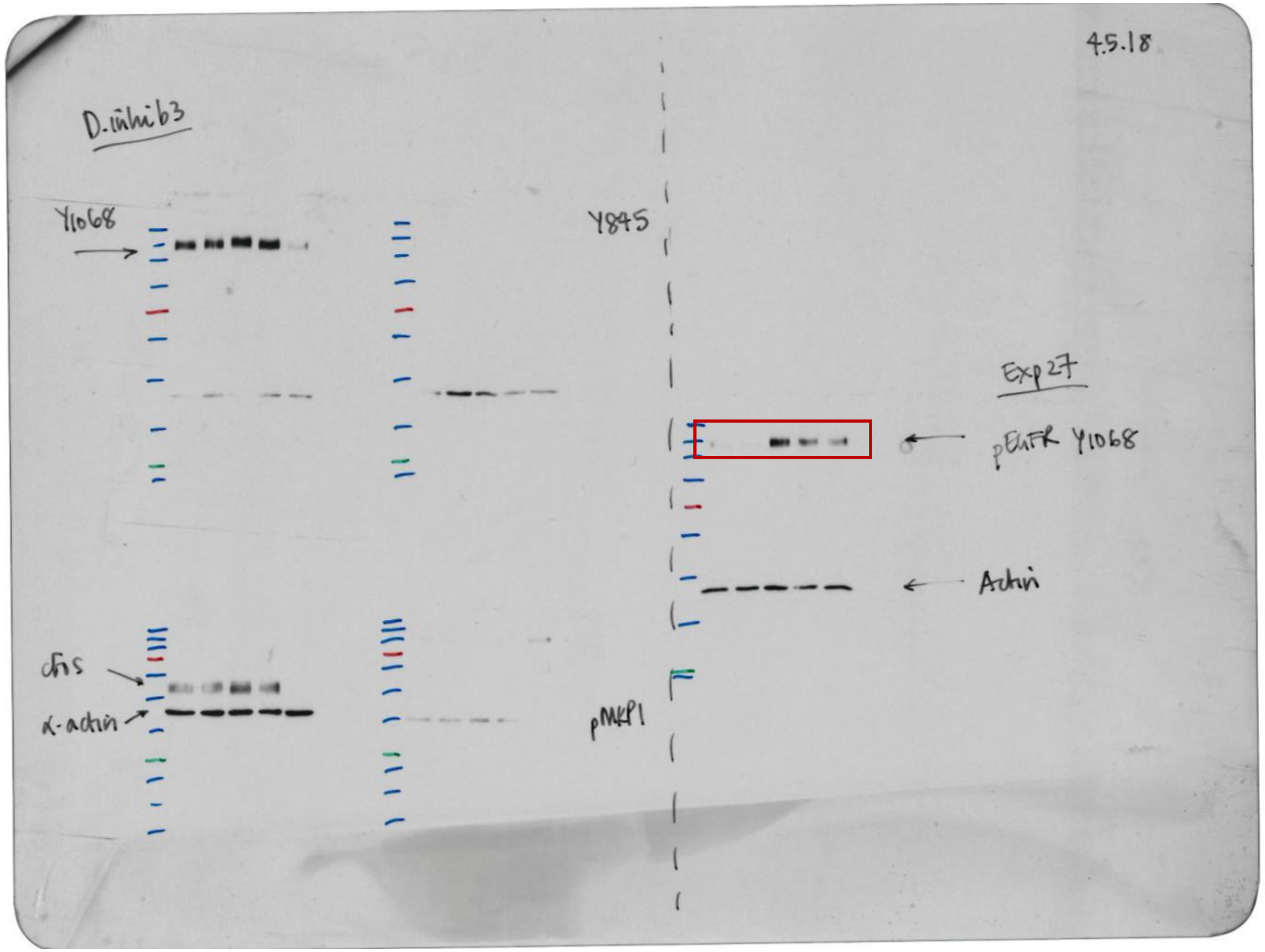

pEGFR Y1068

Fig 2A:

pEGFR Y845

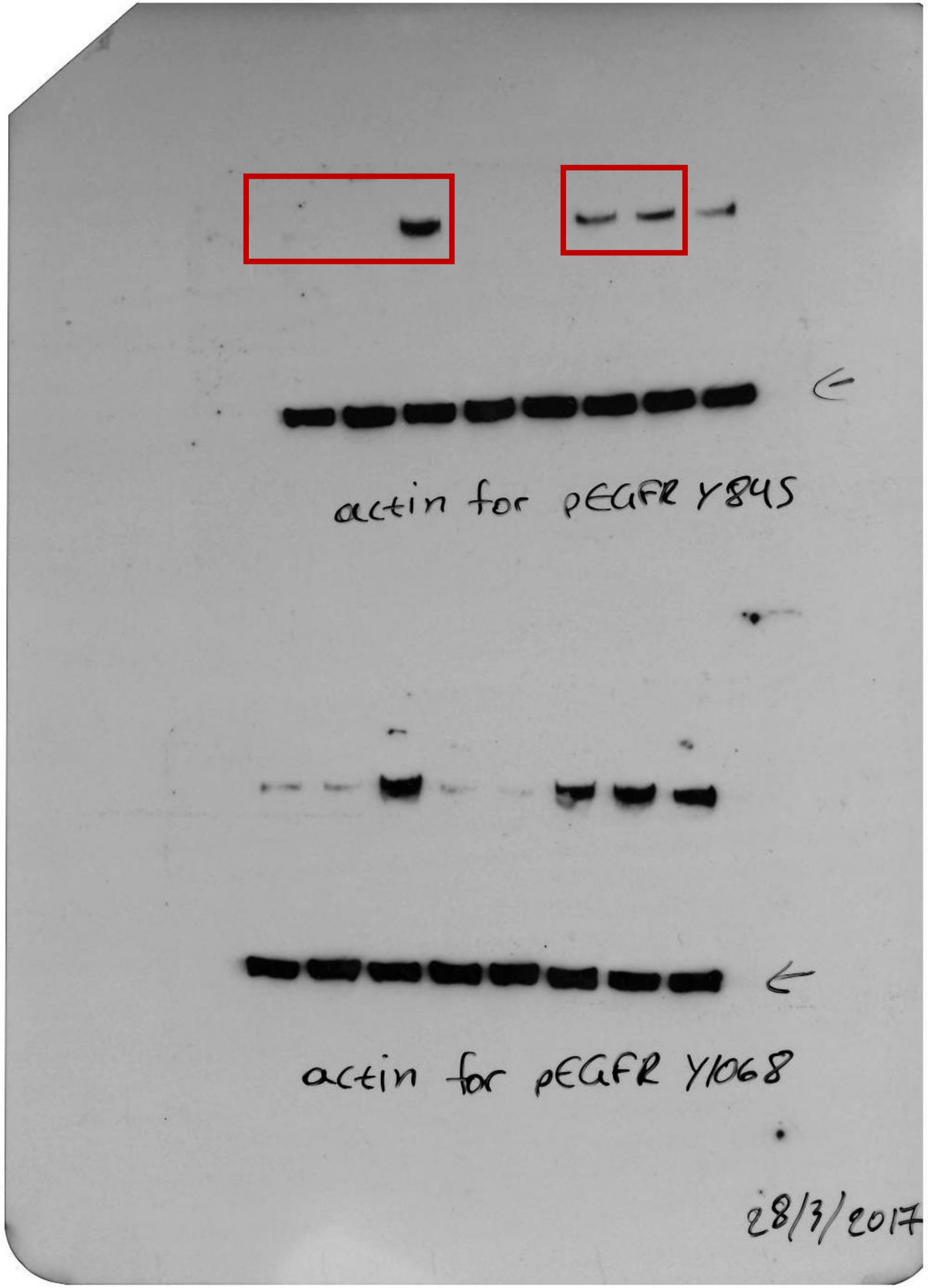

Fig 2A:

cFos

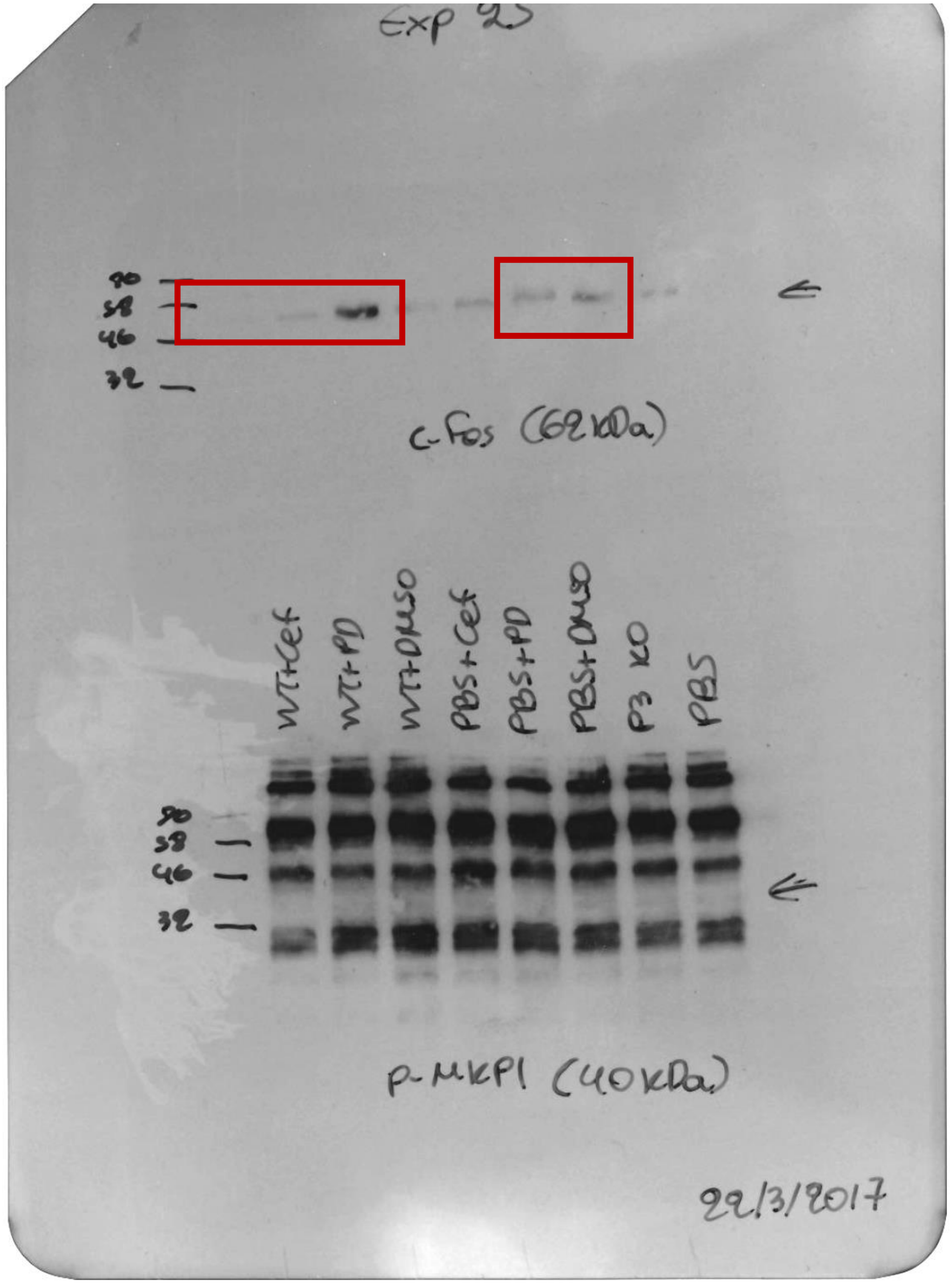

Exp 25
90
58
46
32
c-Fos (62kDa)
WT+Cef
WT+PD
WT+DMSO
PBS+Cef
PBS+PD
PBS+DMSO
P3 KO
PBS
90
58
46
32
p-MKP1 (40kDa)
22/3/2017

Fig 2A:

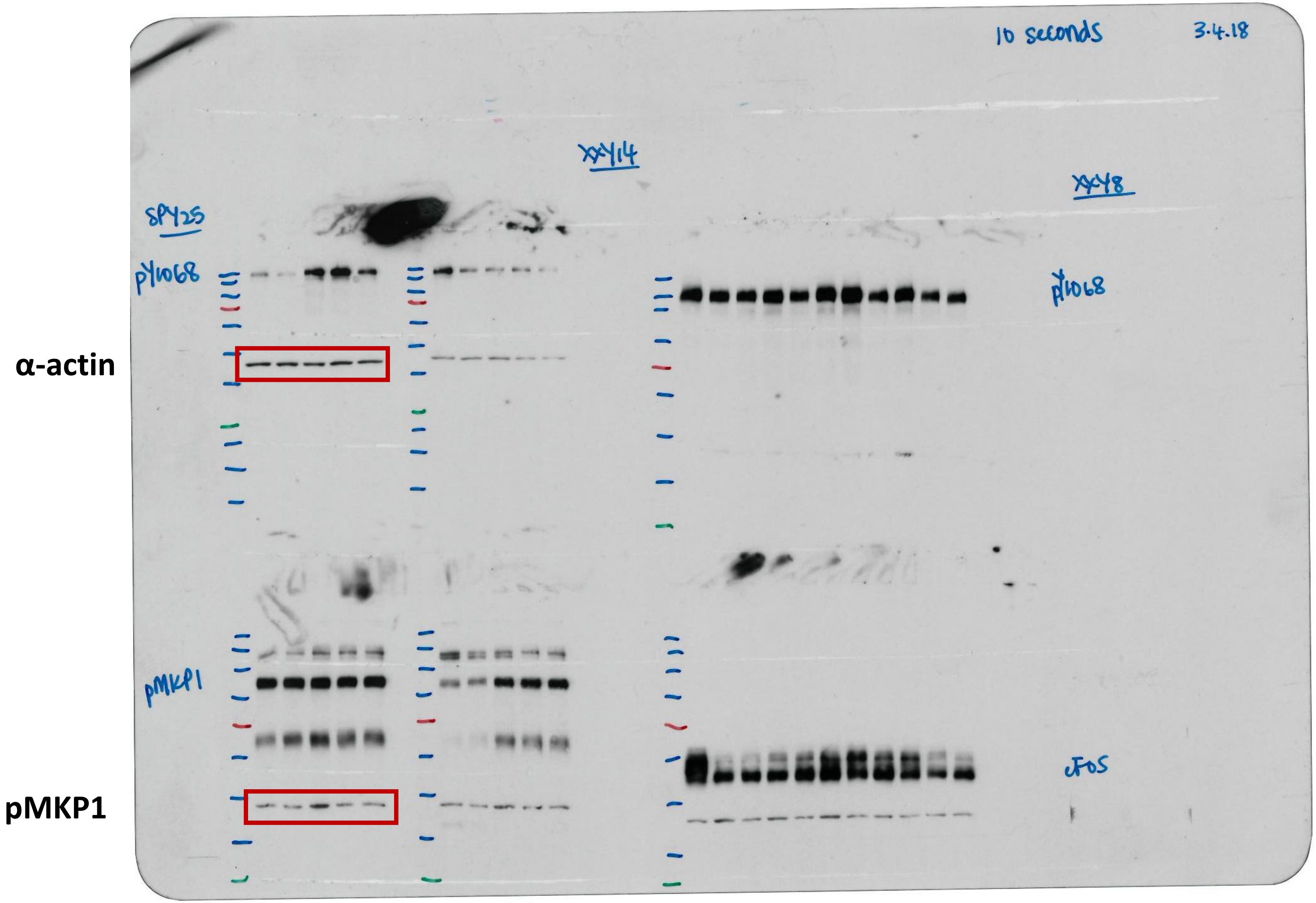
10 seconds
3.4.18
XXY14
XXY8
SPY25
pY1068
α-actin
pMKP1
pMKP1
pY1068
cFos

Fig 2G:

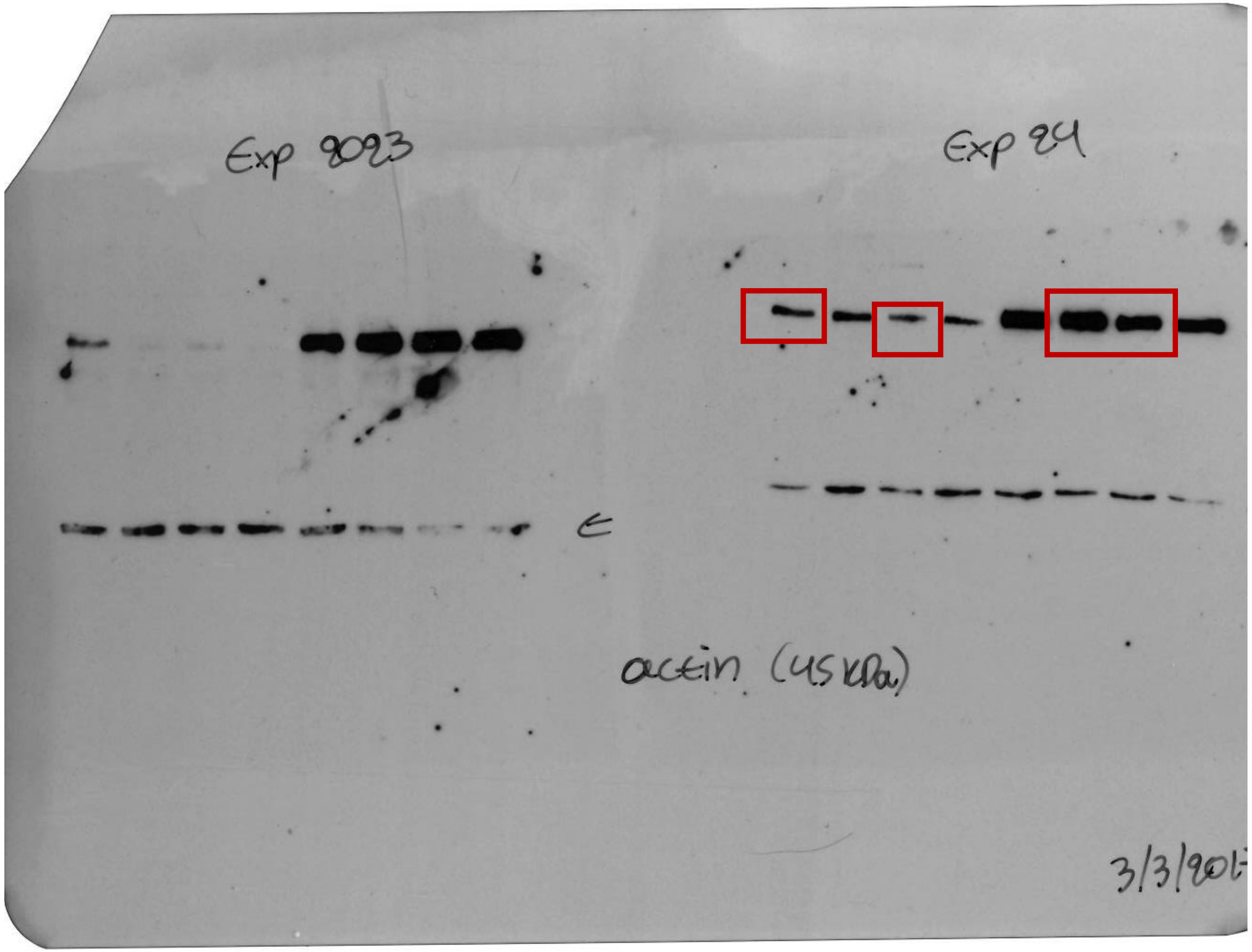

pEGFR Y1068

Fig 2G:

pEGFR Y845

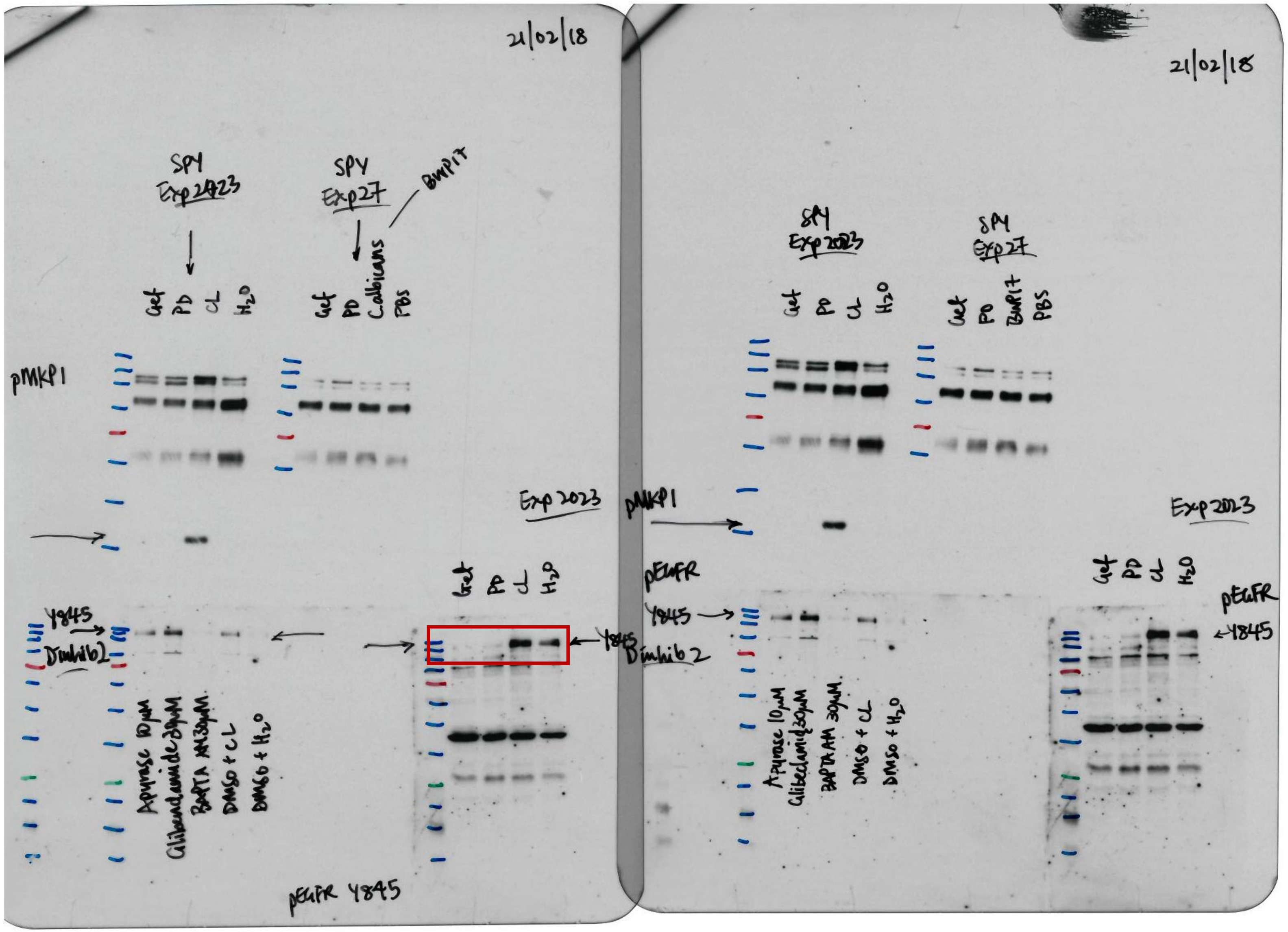

Fig 2G:

c-Fos

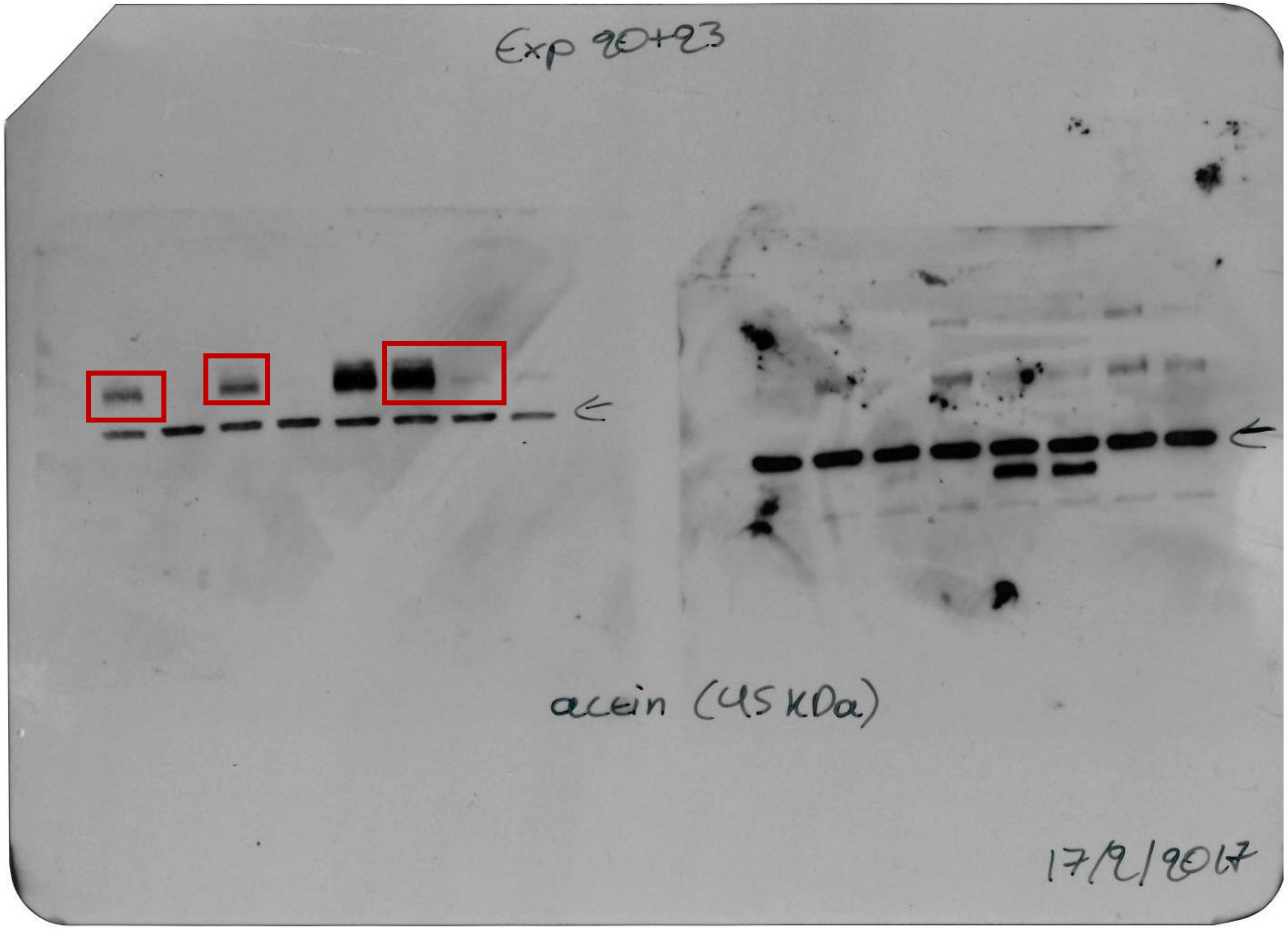

Fig 2G:

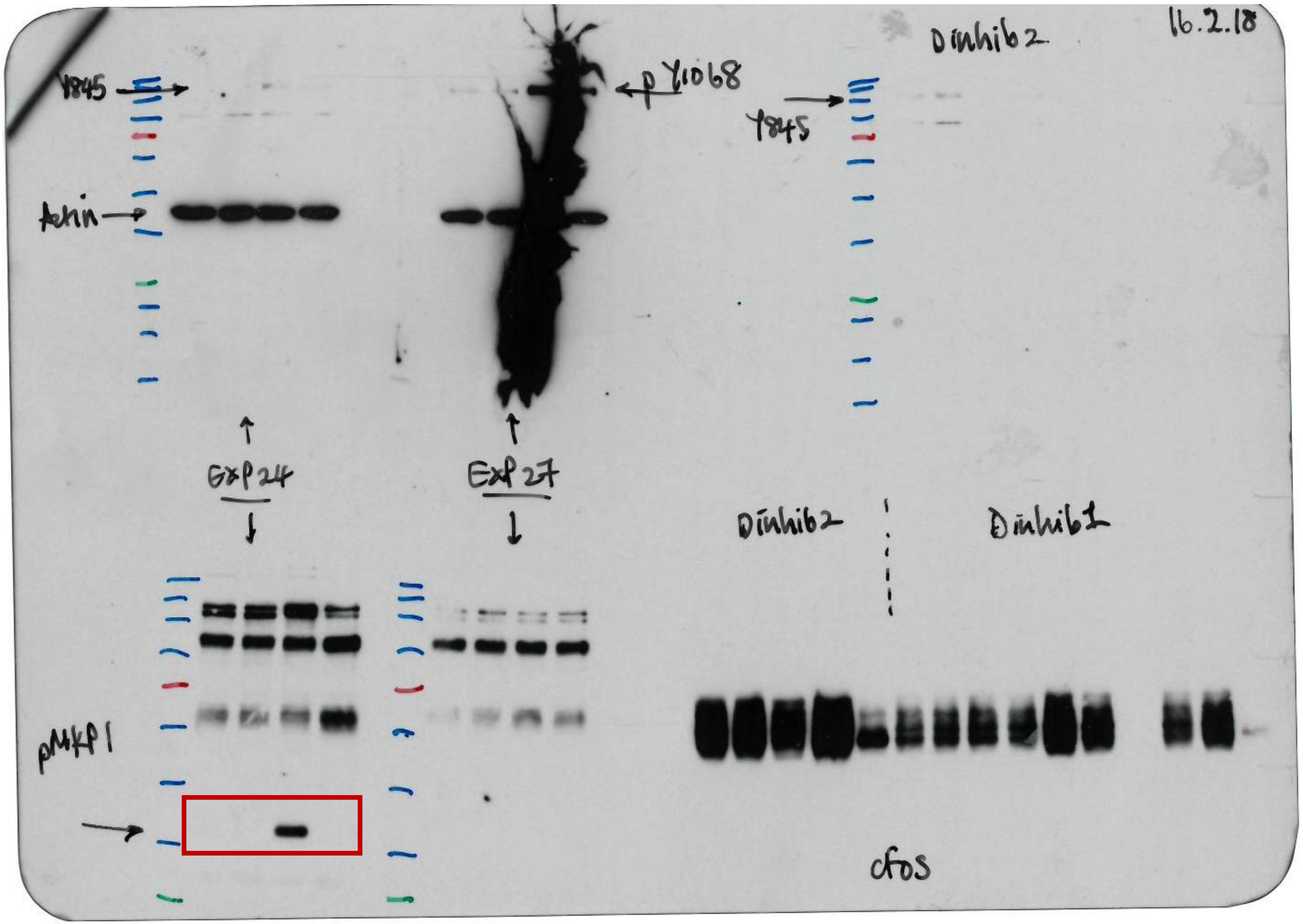
Y845
pY1068
Y845
Actin
Exp 24
Exp 27
Dinhib2
Dinhib1
16.2.18
pMKP1
cfos

pMKP1

Fig 2G:

α-actin

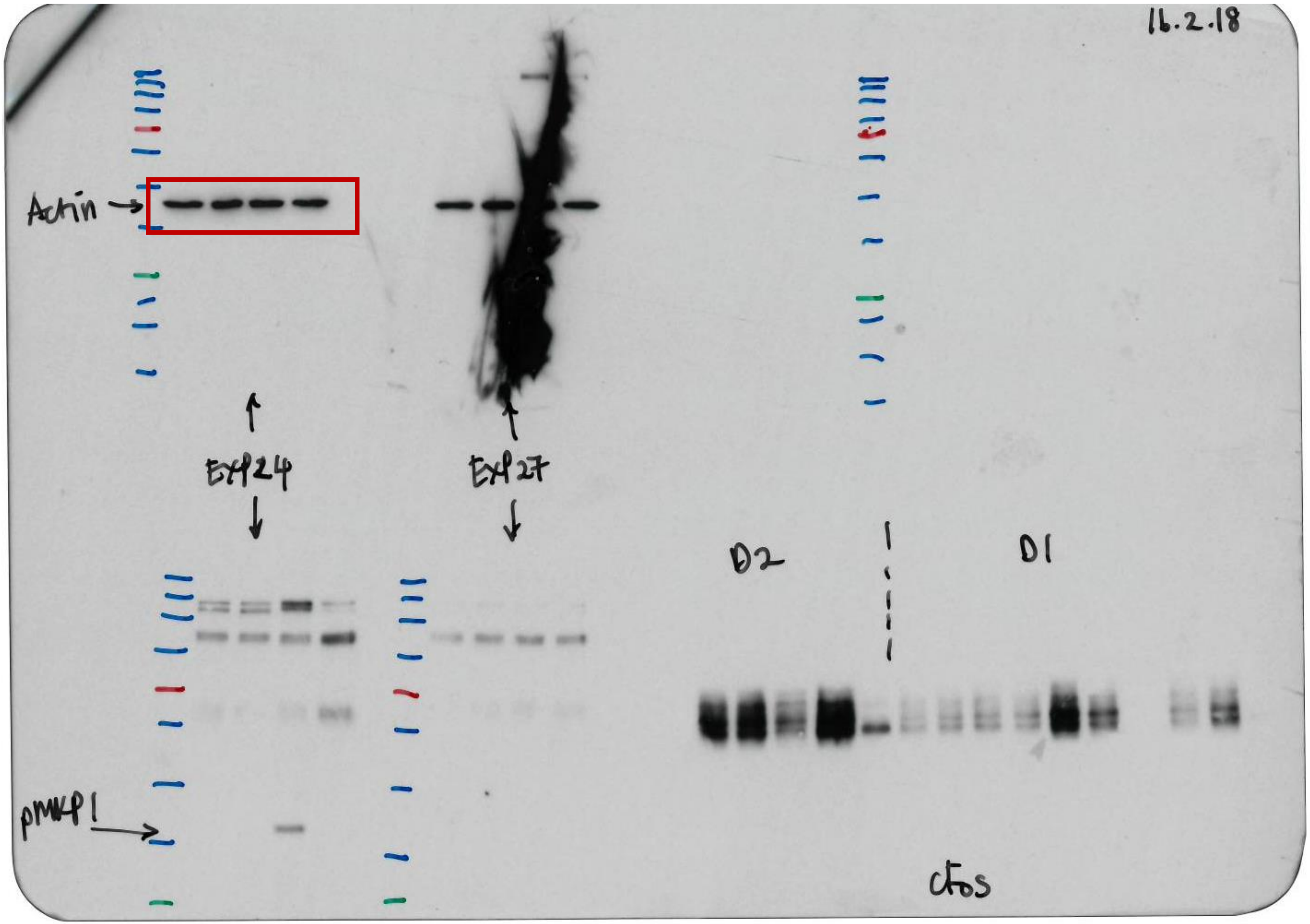

Fig 4J:

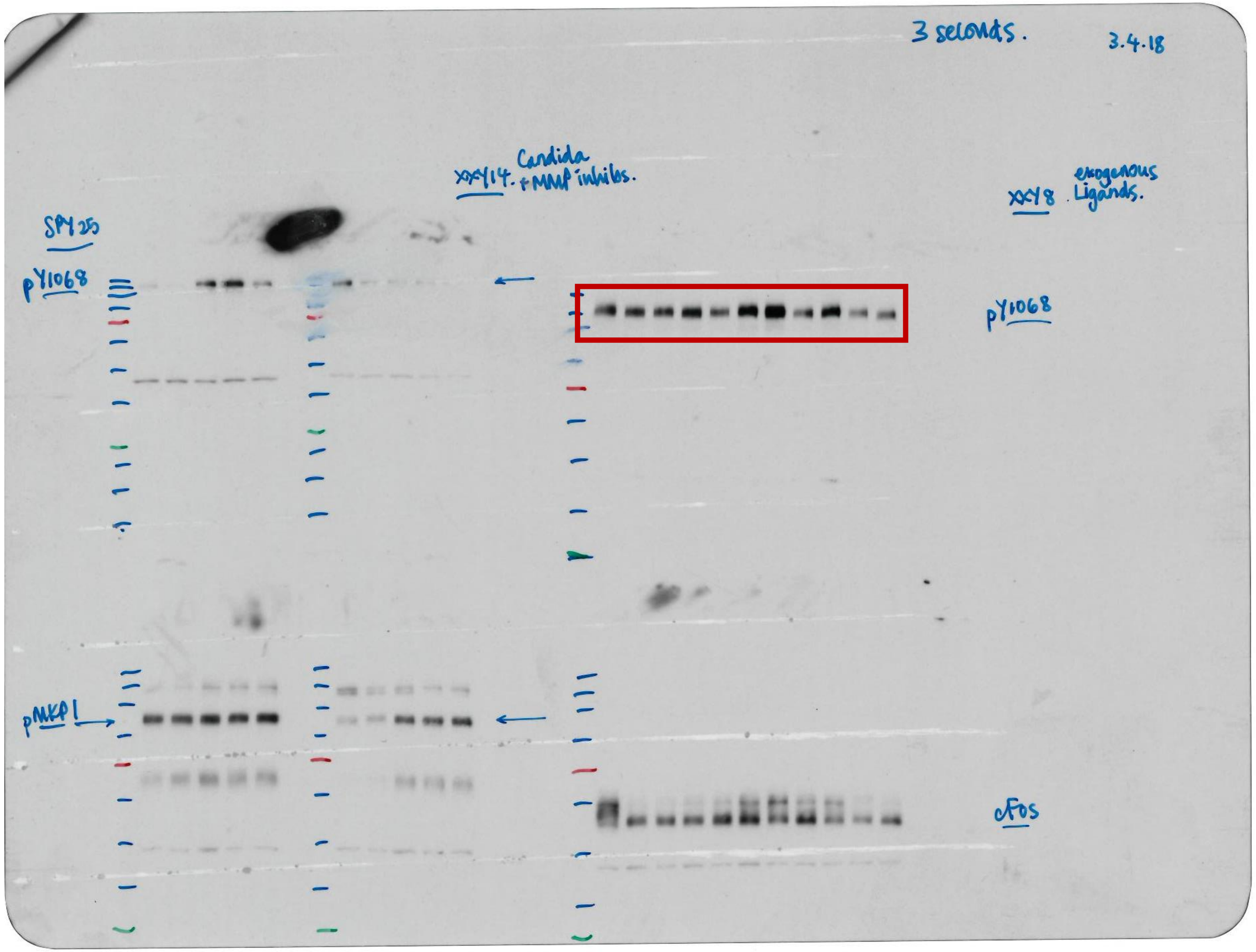

pEGFR Y1068

Fig 4J:

pEGFR Y845

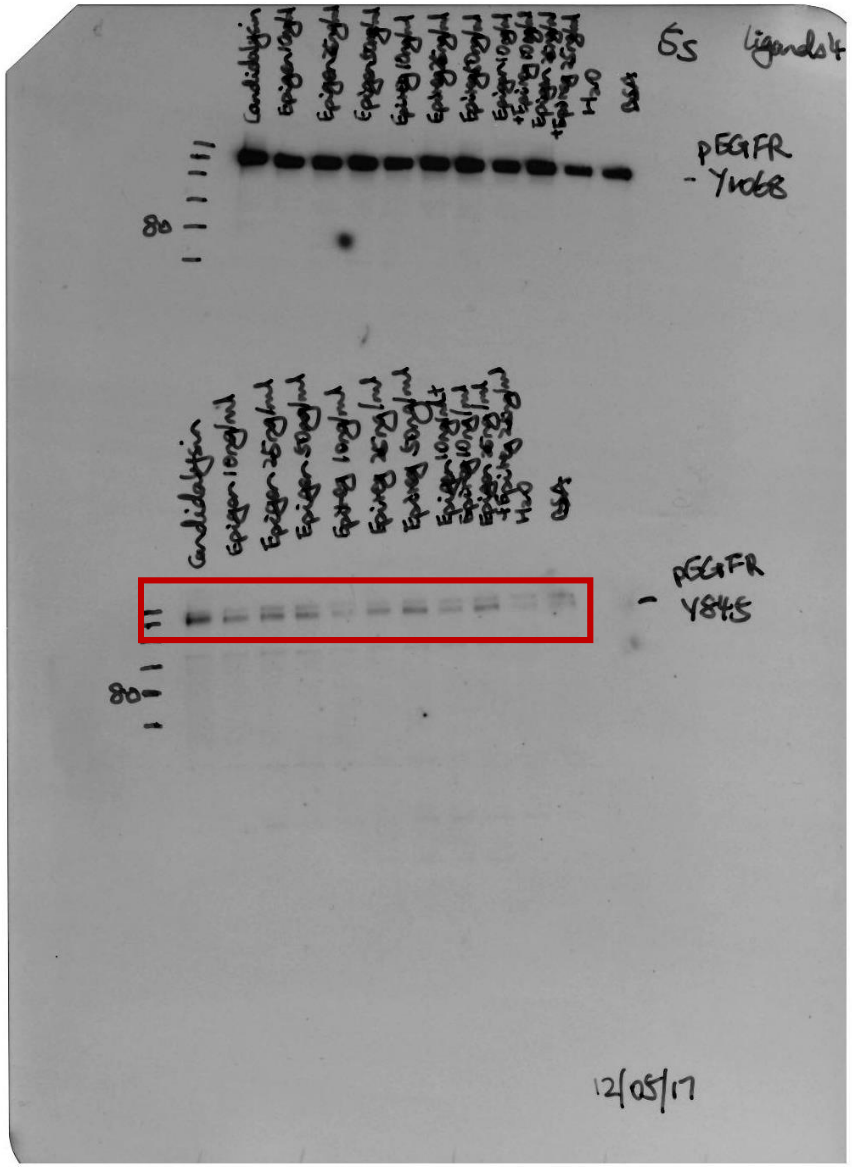

Fig 4J:

c-Fos
α-actin

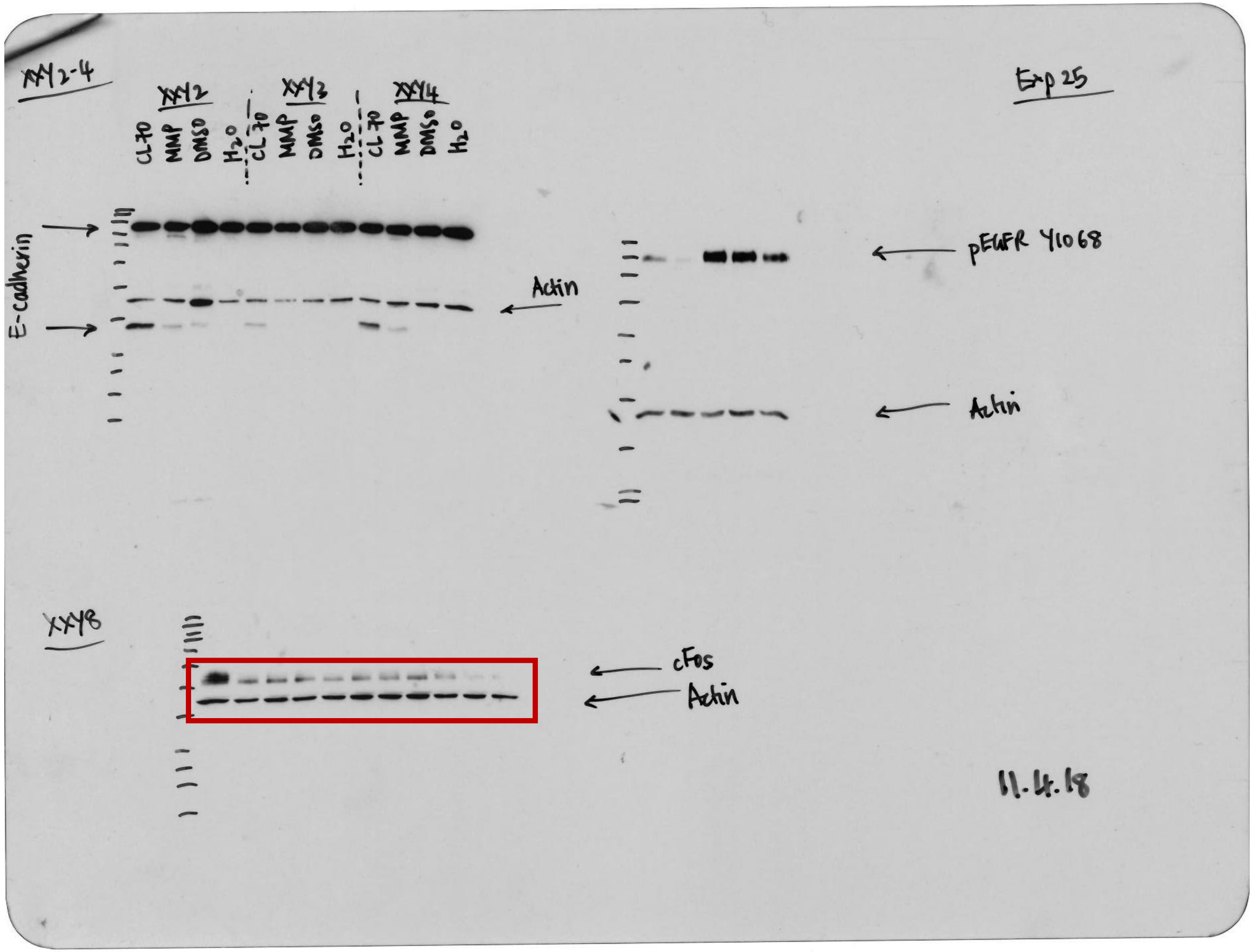

Fig 4J:

p-MKP1

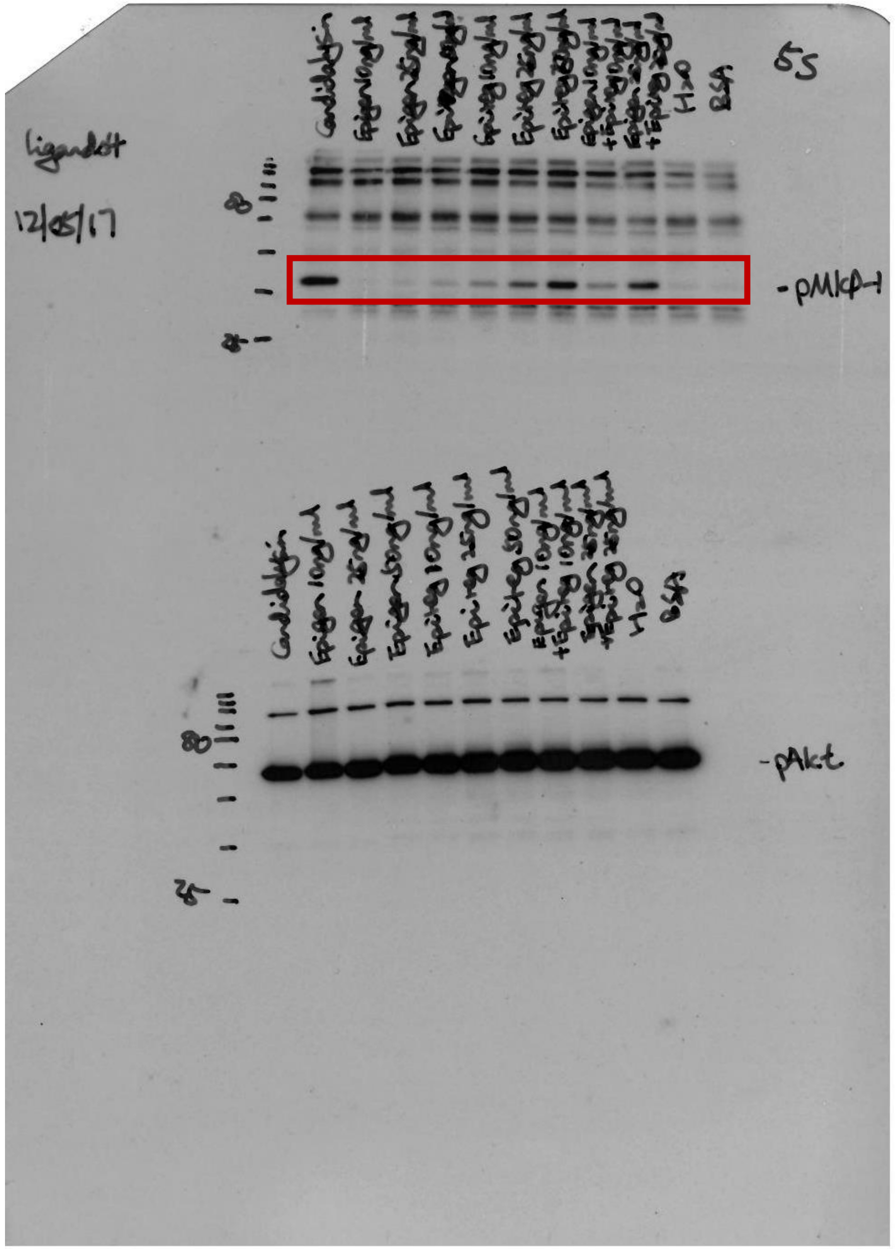

Fig 5A:

pEGFR Y1068

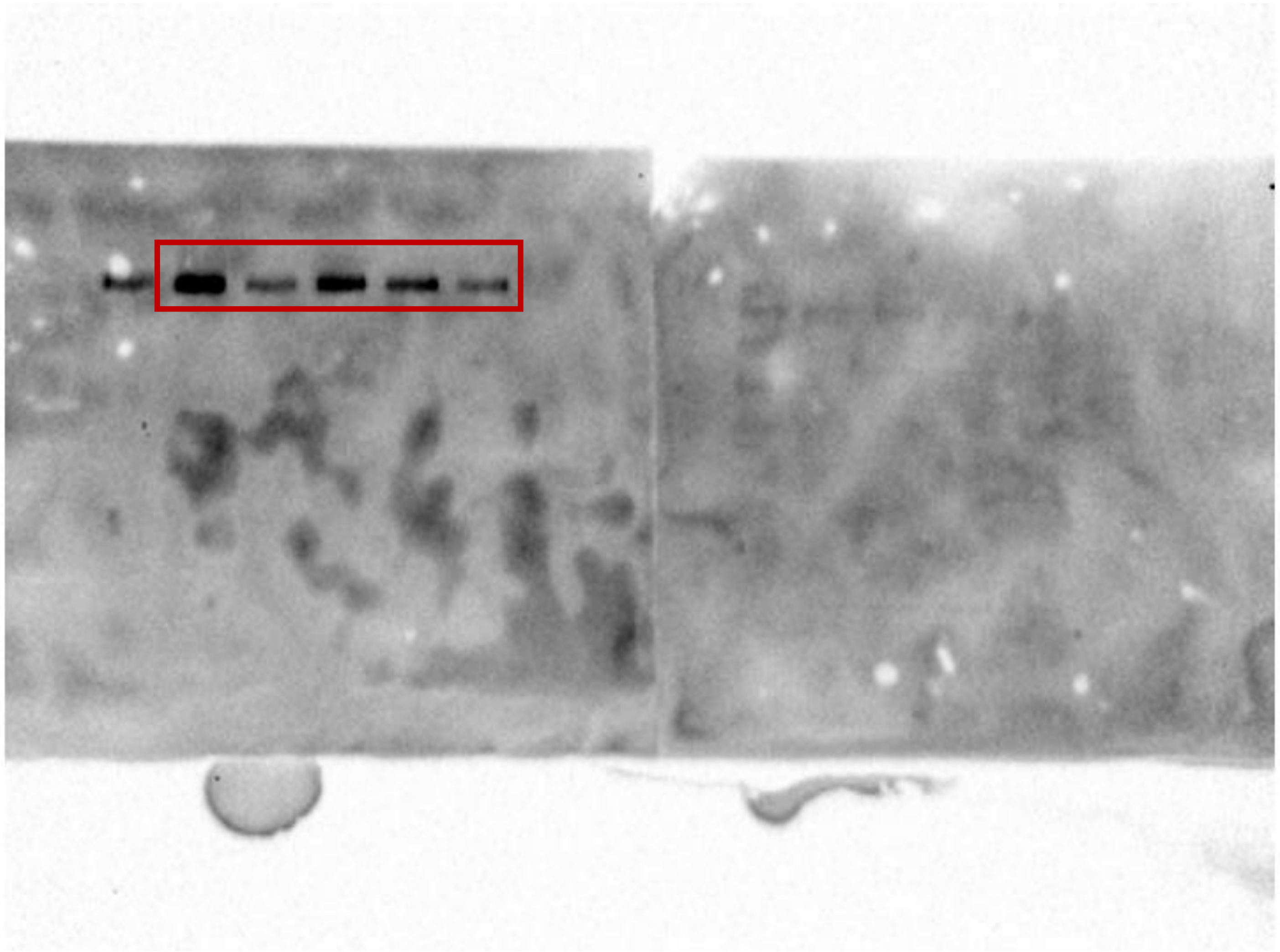

Fig 5A:

pEGFR Y845

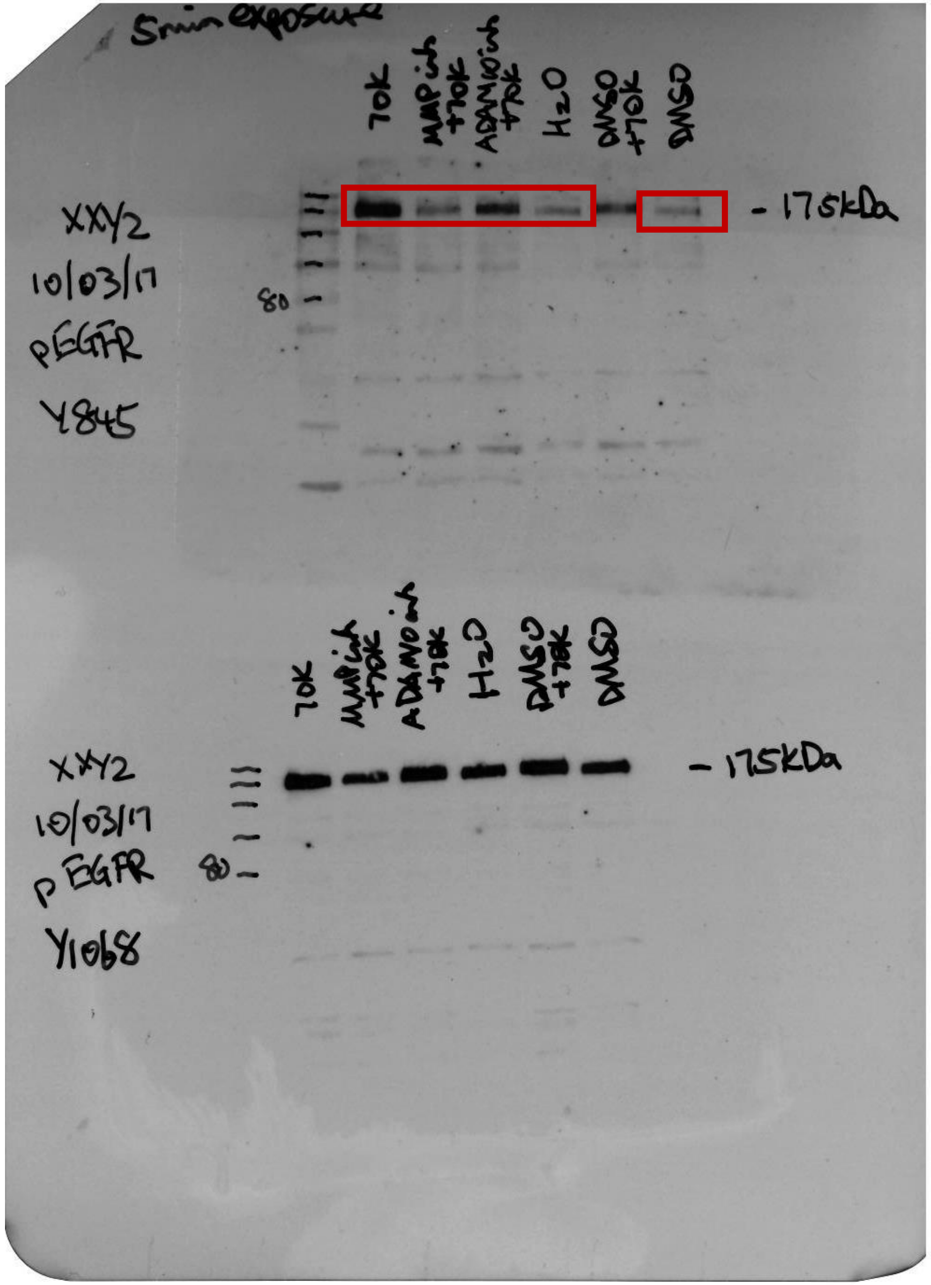

Fig 5A:

c-Fos

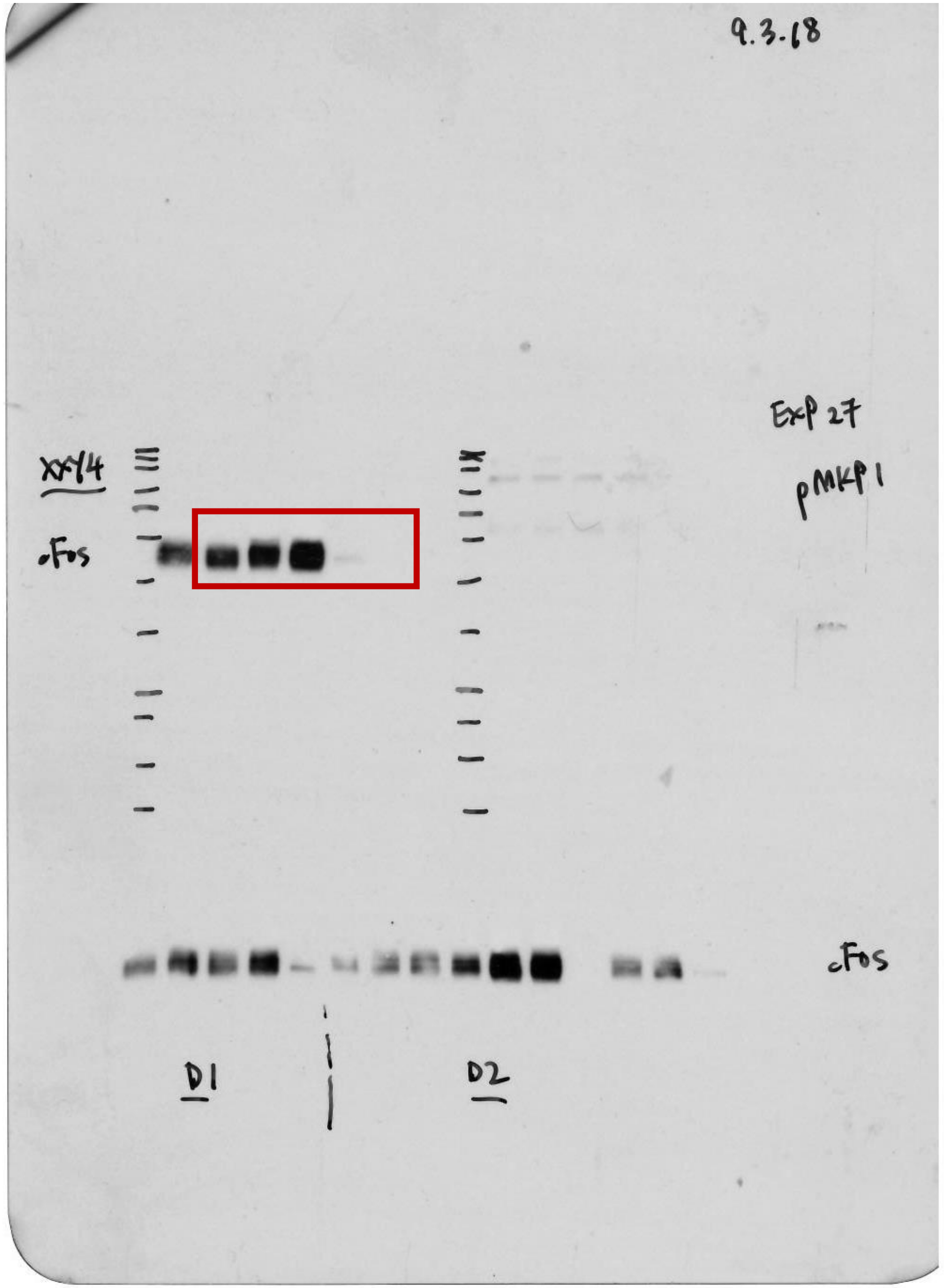

Samples were accidently run in a different order so bands were rearranged to keep in line with other figures.

Fig 5A:

pMKP1

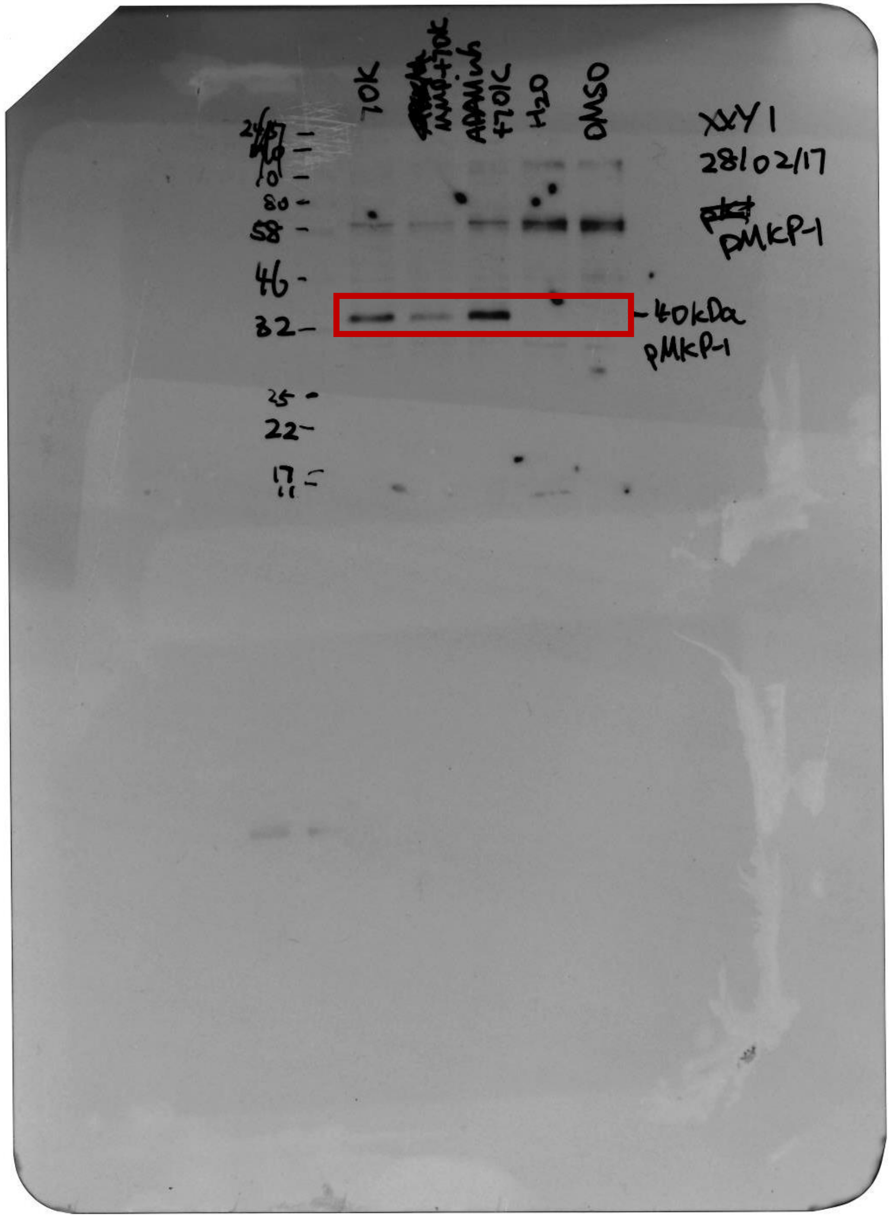

Fig 5A:

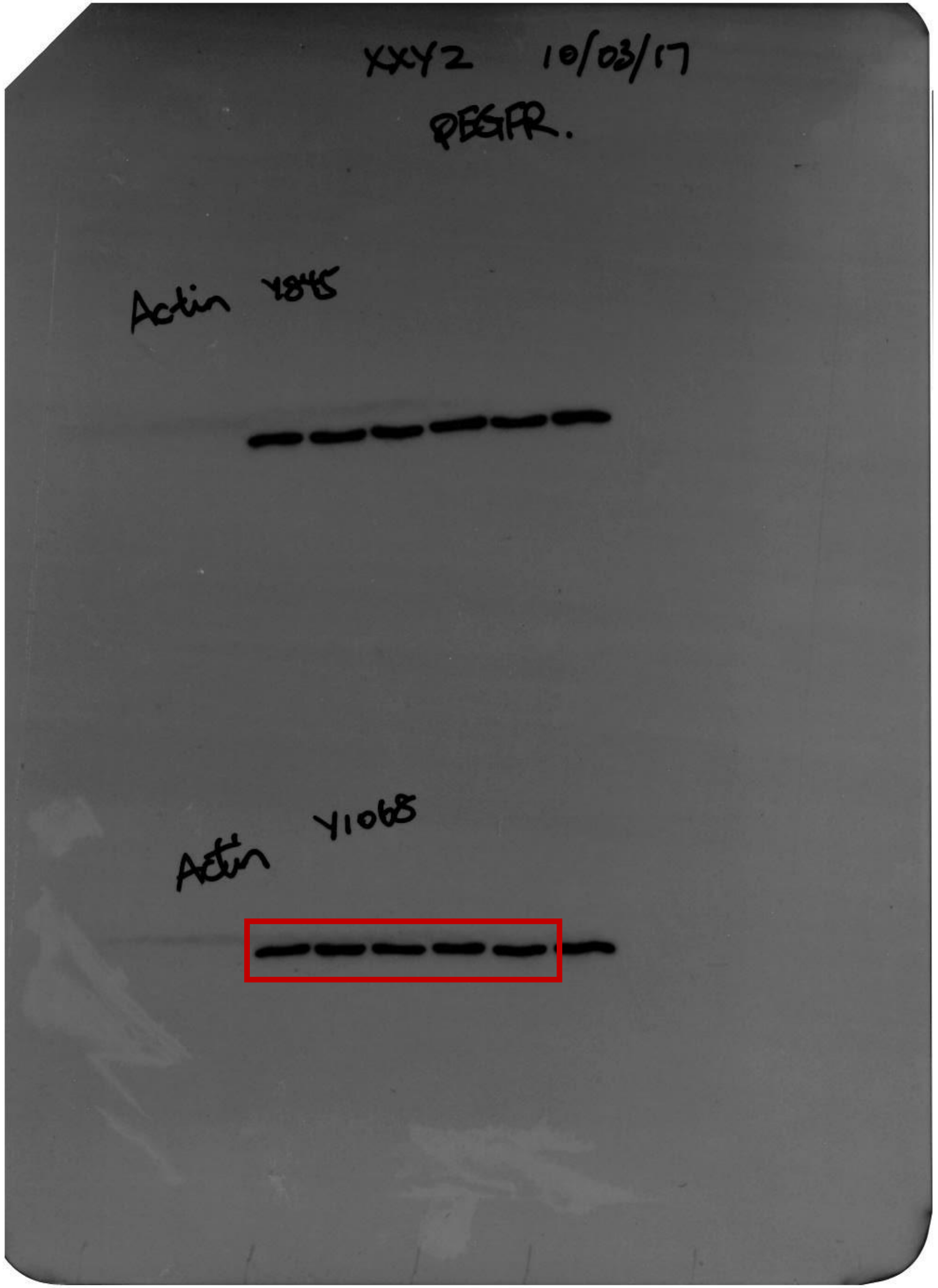

α-actin

Fig 5I:

pEGFR Y1068

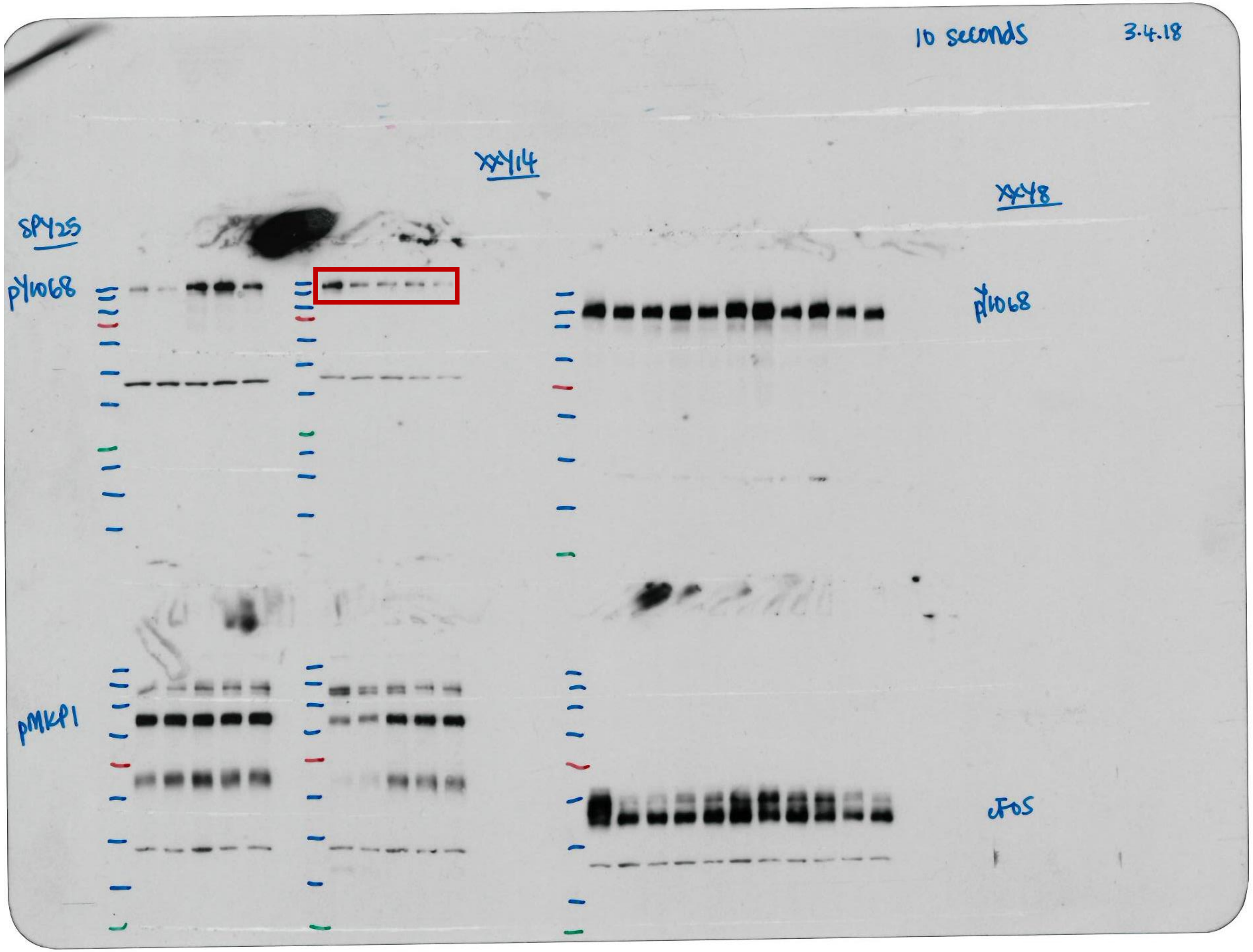

Fig 5I :

pEGFR Y845

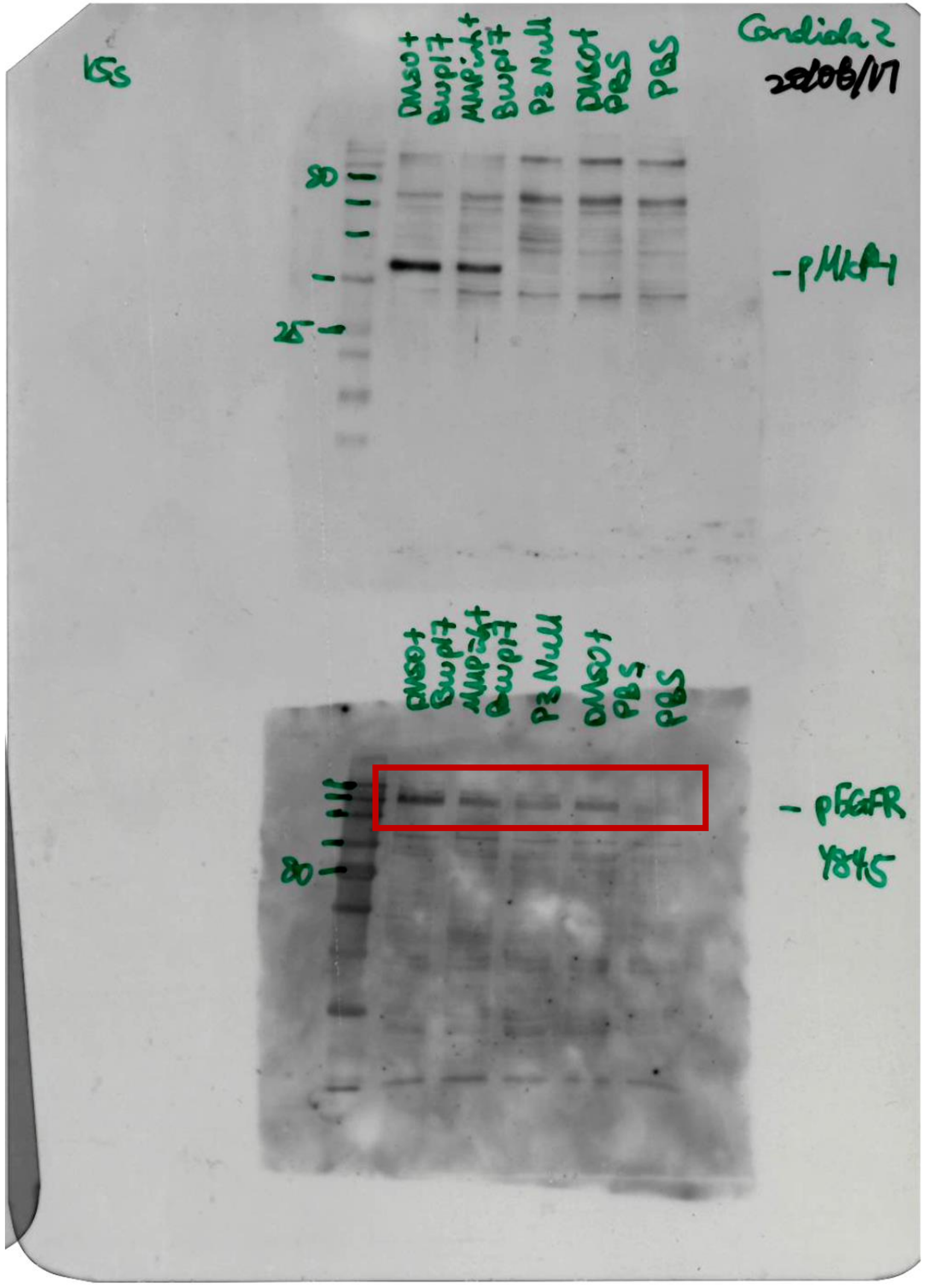

Fig 5I :

c-Fos

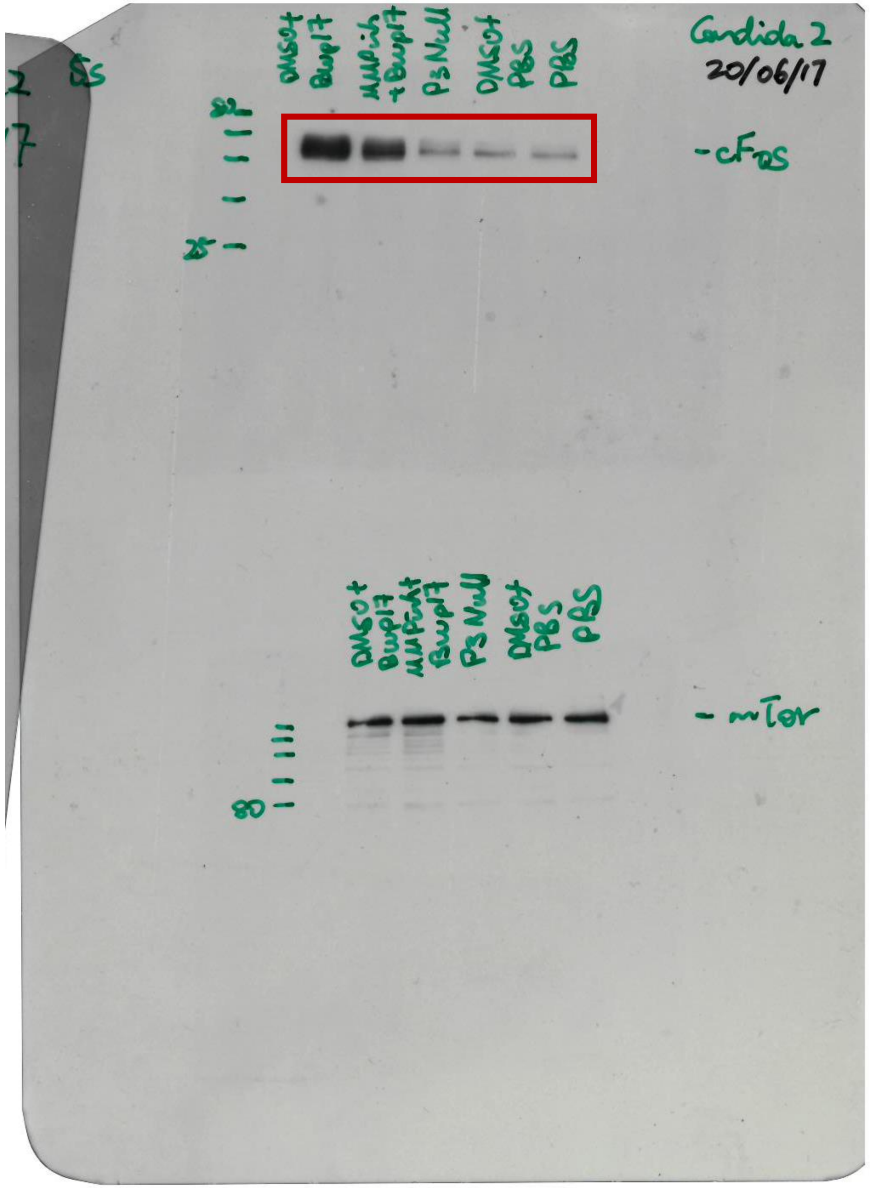

Fig 5I:

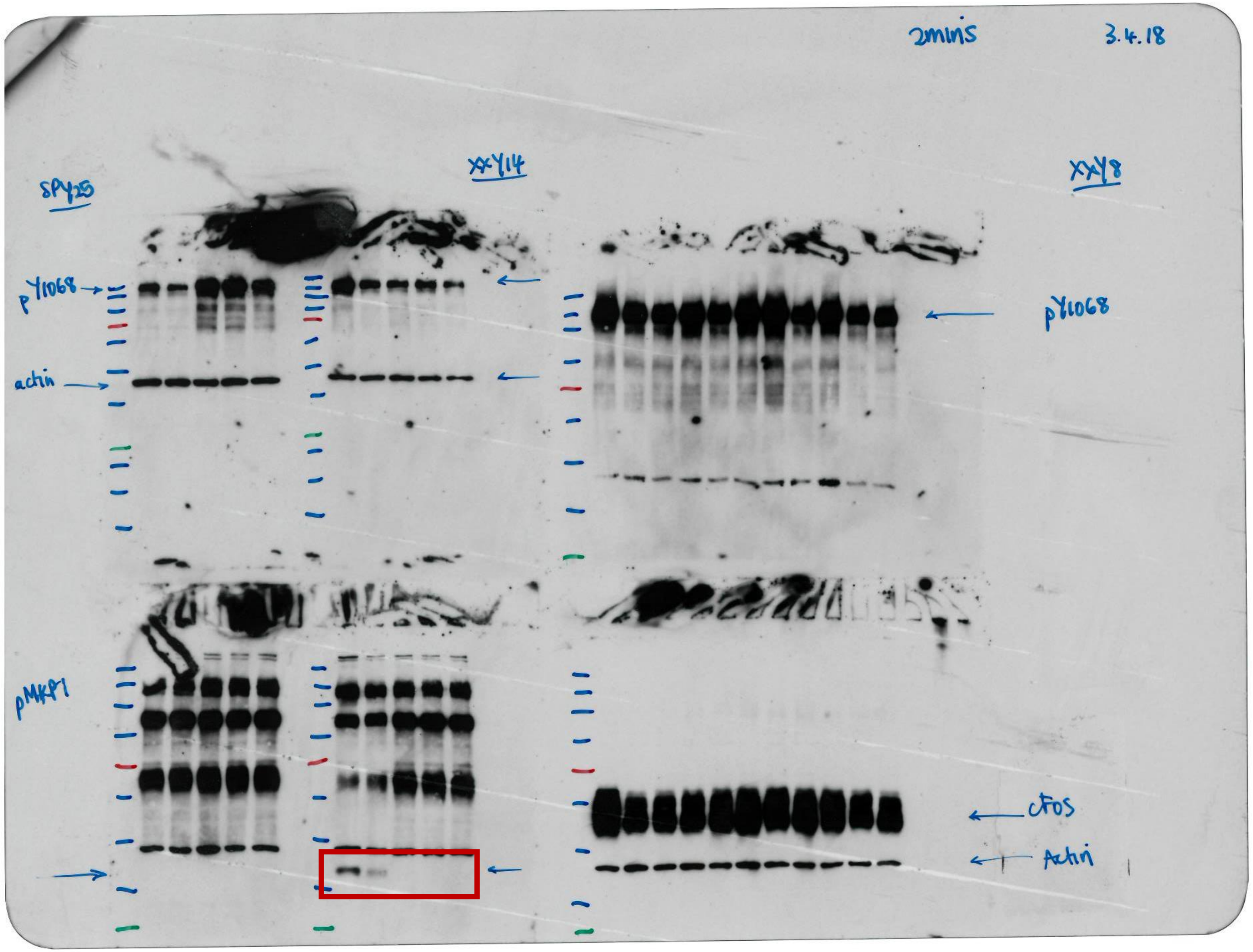
2mins
3.4.18
SPY25
XXY14
XXY8
pY1068
actin
pY1068
pMKP1
cFos
Actin

pMKP1

Fig 5I:

α-actin

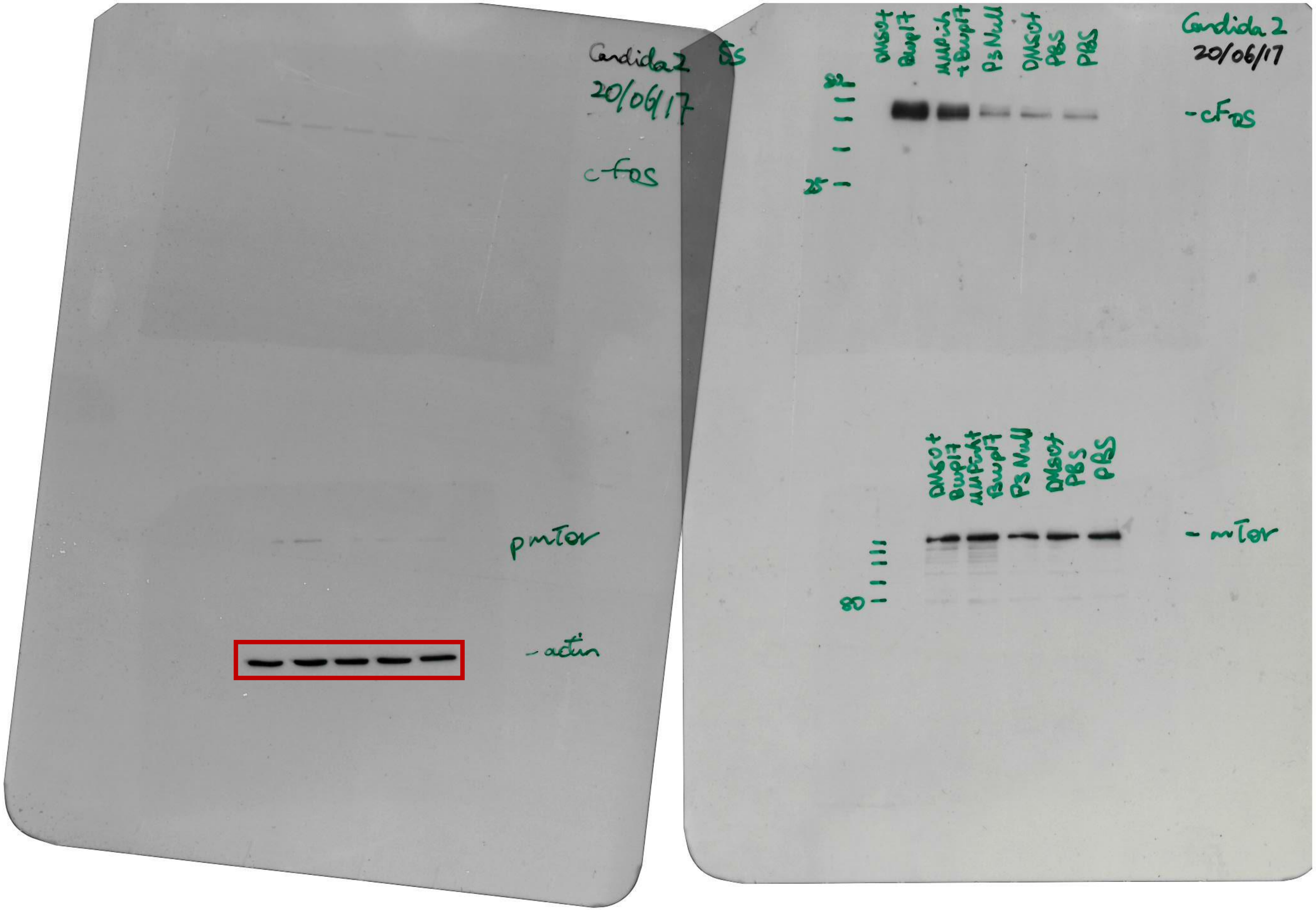

Fig 6A :

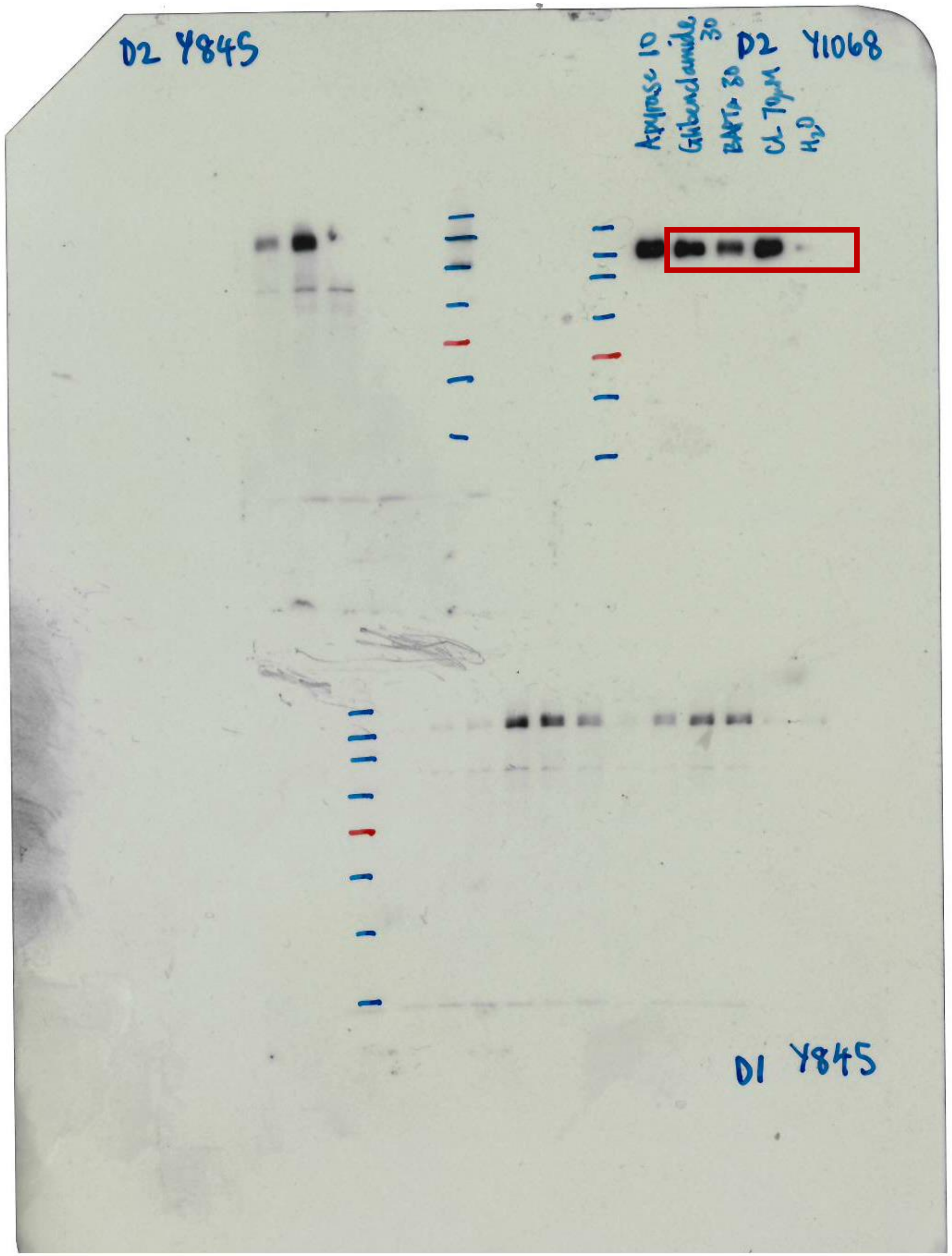

pEGFR Y1068

Fig 6a

pEGFR Y845

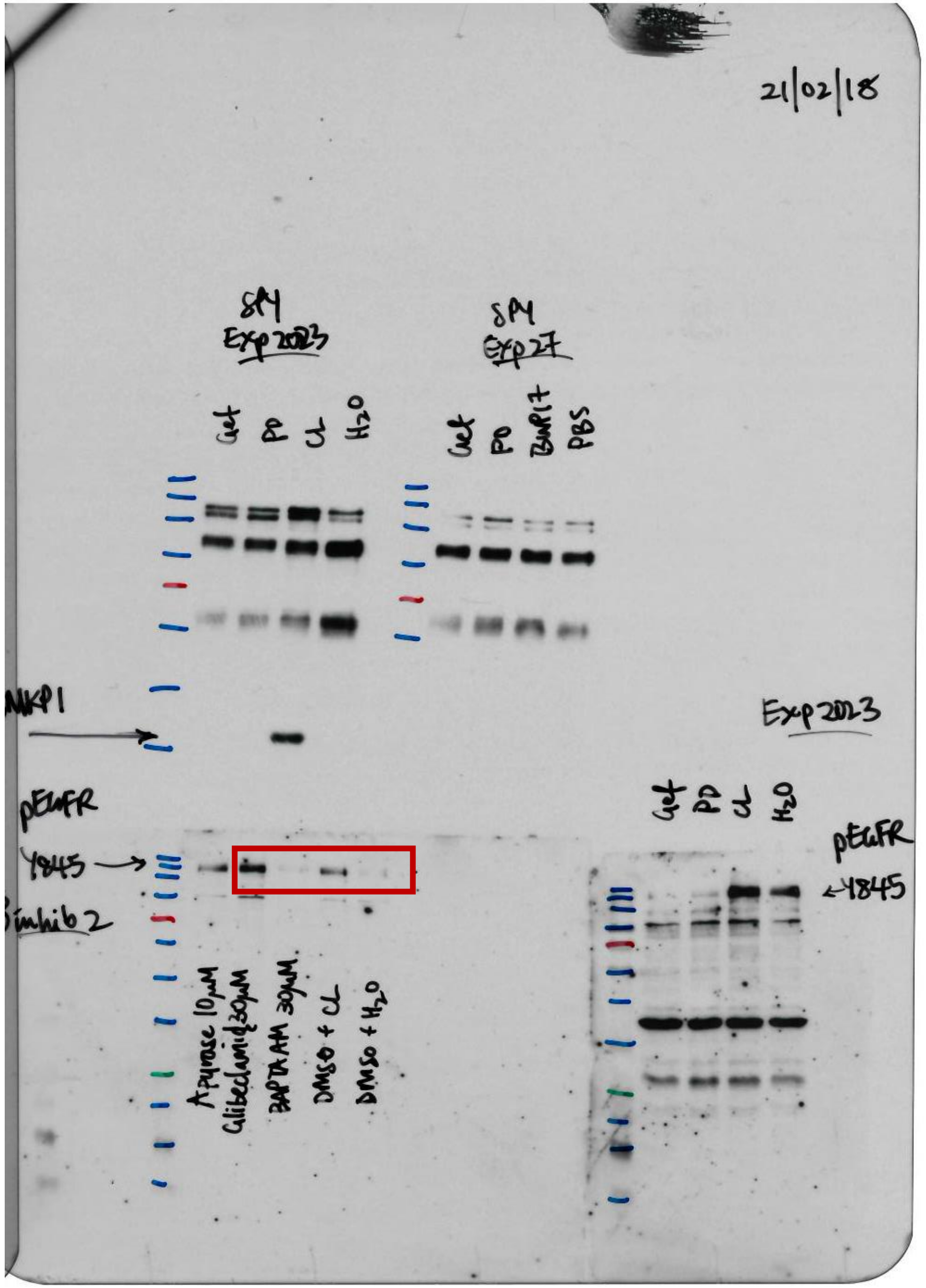

Fig 6a

c-Fos

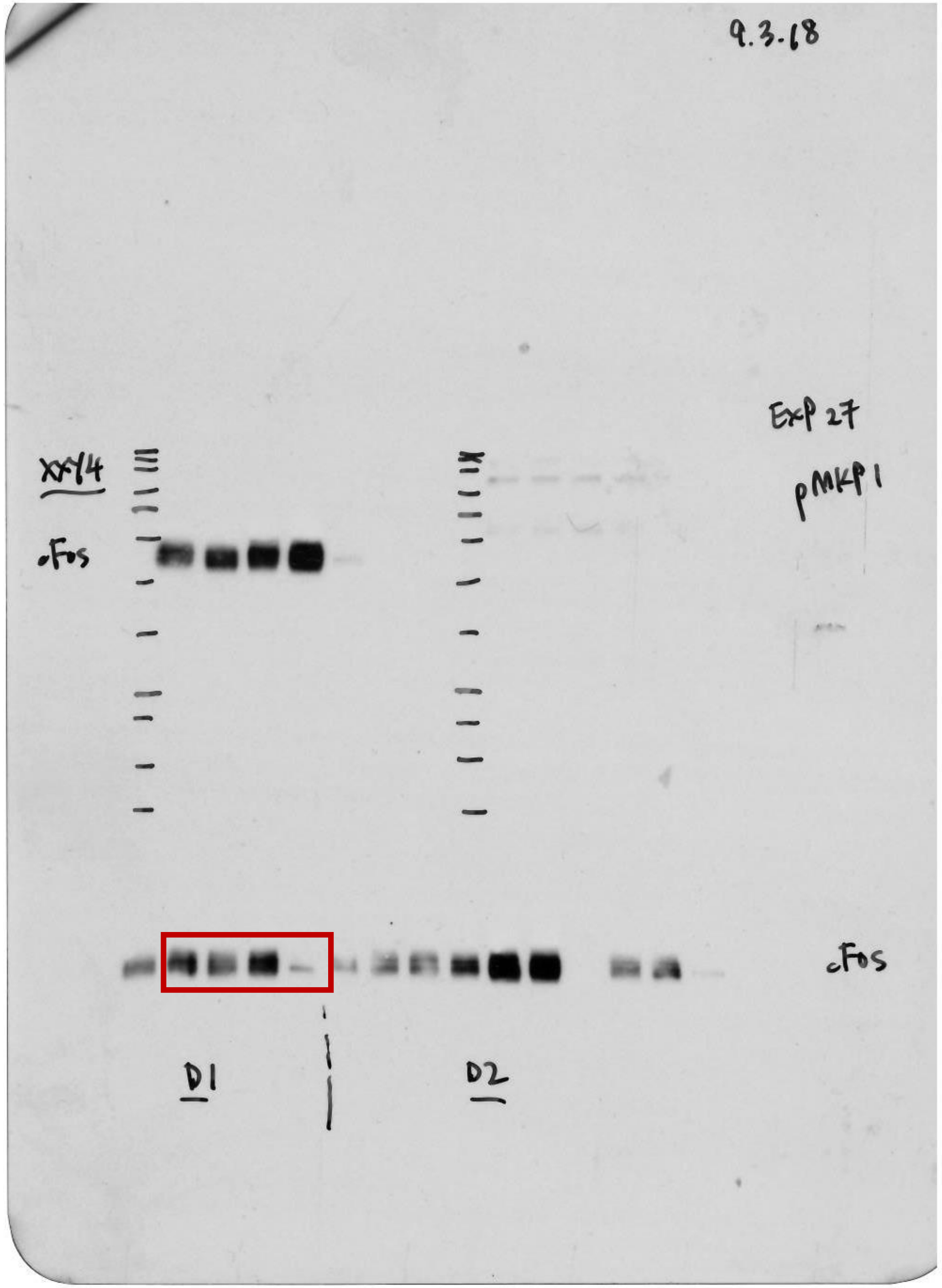

Fig 6a

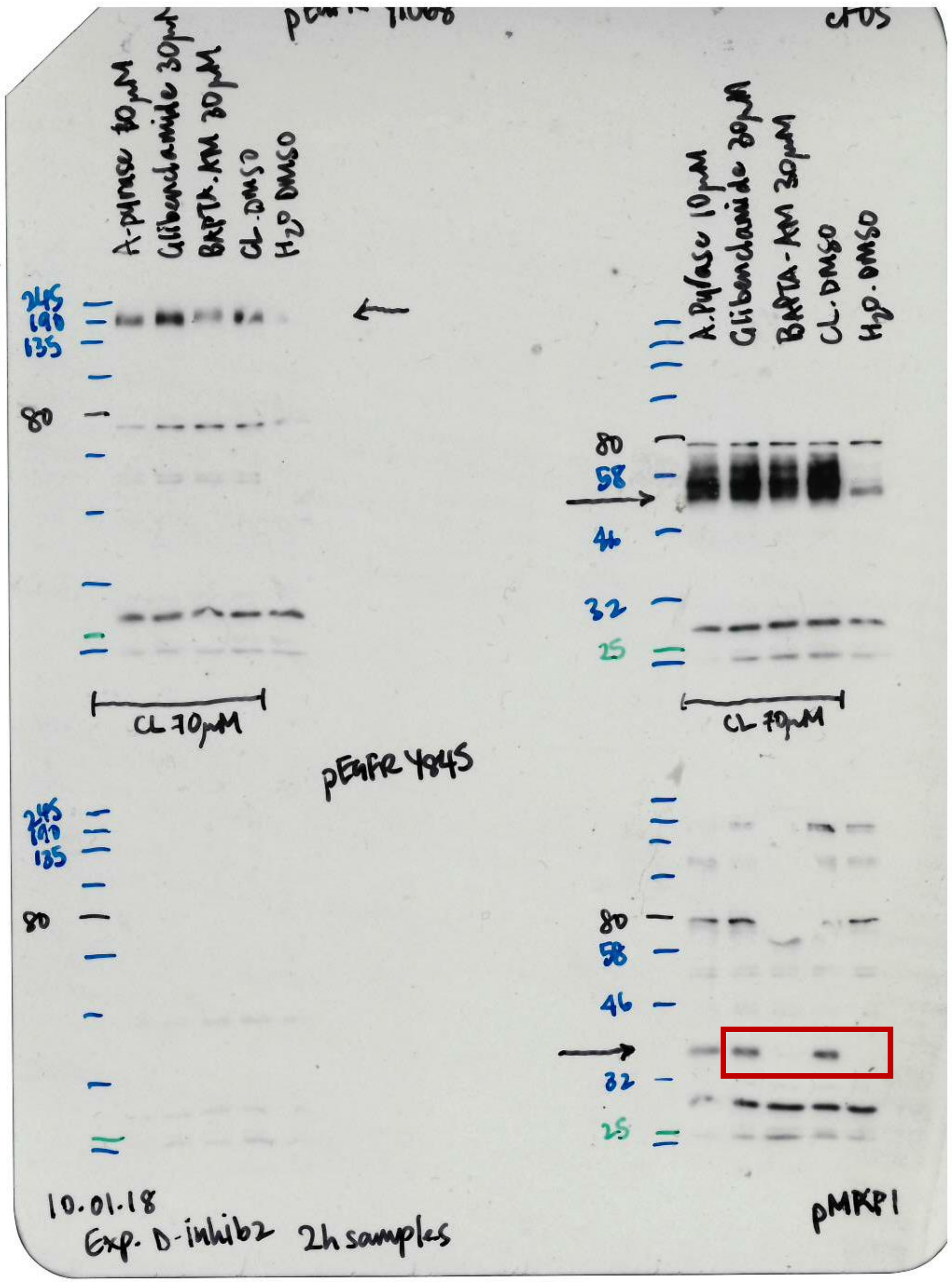
A-pylase 10μM
Glibenclamide 30μM
BAPTA-AM 30μM
CL-DMSO
H2O DMSO
CL 70μM
pEGFR Y845
cFOS
A.Pylase 10μM
Glibenclamide 30μM
BAPTA-AM 30μM
CL-DMSO
H2O-DMSO
245
190
135
80
58
46
32
25
10.01.18
Exp. D-inhib2 2h samples
pMKP1

pMKP1

Fig 6A

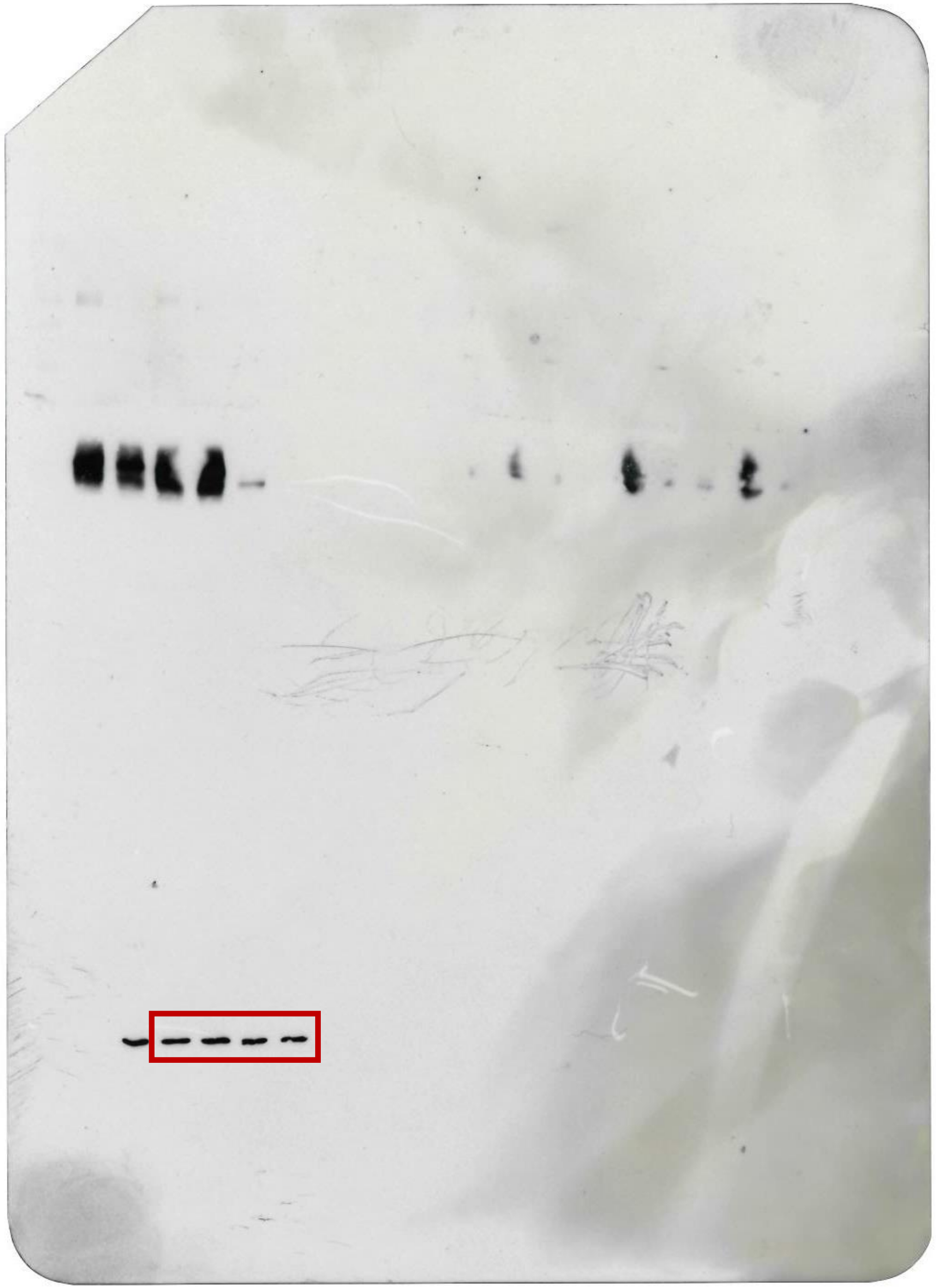

α-actin
