# Supplementary material for: Candidalysin activates innate epithelial immune responses via epidermal growth factor receptor
Source: Nat Commun. 2019 May 24;10:2297. doi: 10.1038/s41467-019-09915-2 (PMC6534540; doi:10.1038/s41467-019-09915-2)
Supplement: Supplementary file 3 — Reporting Summary [file 41467_2019_9915_MOESM3_ESM.pdf]

nature research

Corresponding author(s): *Double-blind peer review submissions: write DBPR and your manuscript number here instead of author names.*

Reporting Summary

Nature Research wishes to improve the reproducibility of the work that we publish. This form provides structure for consistency and transparency in reporting. For further information on Nature Research policies, see Authors & Referees and the Editorial Policy Checklist.

Statistical parameters

When statistical analyses are reported, confirm that the following items are present in the relevant location (e.g. figure legend, table legend, main text, or Methods section).

n/a	Confirmed	
☐	☒	The exact sample size (*n*) for each experimental group/condition, given as a discrete number and unit of measurement
☐	☒	An indication of whether measurements were taken from distinct samples or whether the same sample was measured repeatedly
☐	☒	The statistical test(s) used AND whether they are one- or two-sided<br>*Only common tests should be described solely by name; describe more complex techniques in the Methods section.*
☐	☒	A description of all covariates tested
☐	☒	A description of any assumptions or corrections, such as tests of normality and adjustment for multiple comparisons
☐	☒	A full description of the statistics including central tendency (e.g. means) or other basic estimates (e.g. regression coefficient) AND variation (e.g. standard deviation) or associated estimates of uncertainty (e.g. confidence intervals)
☐	☒	For null hypothesis testing, the test statistic (e.g. *F*, *t*, *r*) with confidence intervals, effect sizes, degrees of freedom and *P* value noted<br>*Give P values as exact values whenever suitable.*
☒	☐	For Bayesian analysis, information on the choice of priors and Markov chain Monte Carlo settings
☒	☐	For hierarchical and complex designs, identification of the appropriate level for tests and full reporting of outcomes
☒	☐	Estimates of effect sizes (e.g. Cohen's *d*, Pearson's *r*), indicating how they were calculated
☐	☒	Clearly defined error bars<br>*State explicitly what error bars represent (e.g. SD, SE, CI)*

Our web collection on statistics for biologists may be useful.

Software and code

Policy information about availability of computer code

Data collection	No software used
Data analysis	Gene array analyses software: Database for Annotation, Visualisation and Integrated Discovery (DAVID)

For manuscripts utilizing custom algorithms or software that are central to the research but not yet described in published literature, software must be made available to editors/reviewers upon request. We strongly encourage code deposition in a community repository (e.g. GitHub). See the Nature Research guidelines for submitting code & software for further information.

Data

Policy information about availability of data

All manuscripts must include a data availability statement. This statement should provide the following information, where applicable:

- Accession codes, unique identifiers, or web links for publicly available datasets
- A list of figures that have associated raw data
- A description of any restrictions on data availability

All data generated or analysed during this study are included in this published article (and its supplementary information files), or available in the ArrayExpress (EBI suite) repository, https://www.ebi.ac.uk/arrayexpress/experiments/E-MTAB-7681.

Field-specific reporting

Please select the best fit for your research. If you are not sure, read the appropriate sections before making your selection.

☒ Life sciences ☐ Behavioural & social sciences ☐ Ecological, evolutionary & environmental sciences

For a reference copy of the document with all sections, see nature.com/authors/policies/ReportingSummary-flat.pdf

Life sciences study design

All studies must disclose on these points even when the disclosure is negative.

Sample size	No sample size calculations were performed. Sample sizes were based on previously published data and experimental work from within the group and other publications using similar models. These date back several years, spanning over a range of journals and publications, and have been peer reviewed multiple times.
Data exclusions	Data exclusions were not pre-established. Data were excluded where the investigator noted an technical error that may have affected results. Where errors were determined (based on results e.g. whether control samples performed as expected), they were excluded from analyses.
Replication	Data from Supp Fig.6 was acquired from 1 experiment (6 mice per group). The results correspond with previously published data and show statistical significance. Repeated experimentation would have been unethical as the findings would not have been novel or able to improve upon the obtained dataset. Data from Fig. 1D was obtained from 1 experiment (5 mice per group). All other findings were reproducible.
Randomization	All allocation was conducted at random
Blinding	Murine studies were not blinded as experimentation was conducted by one senior PIL holder and one newly trained investigator. Experiment size and animal numbers were high, providing difficulties in ensuring both blinding and experimental accuracy were achieved. IHC staining and quantification however was blinded. Zebrafish experiments were not blinded as only 1 investigator conducted each study.

Reporting for specific materials, systems and methods

Materials & experimental systems

n/a	Involved in the study	
☒	☐	Unique biological materials
☐	☒	Antibodies
☐	☒	Eukaryotic cell lines
☒	☐	Palaeontology
☐	☒	Animals and other organisms
☒	☐	Human research participants

Methods

n/a	Involved in the study	
☒	☐	ChIP-seq
☒	☐	Flow cytometry
☒	☐	MRI-based neuroimaging

Antibodies

Antibodies used

Cell Signalling Technologies:
- Phospho-EGF Receptor (Tyr1068) (D7A5) XP® Rabbit mAb #3777
- Phospho-EGF Receptor (Tyr845) (D63B4) Rabbit mAb, #6963
- c-Fos (9F6) Rabbit mAb #2250
- Phospho-DUSP1/MKP1 (Ser359) (125E2) Rabbit mAb #2857

Biolegend:
- APC anti-human EGFR Antibody [Clone: AY13] #352905

Jackson ImmunoResearch:
- Peroxidase-AffiniPure Goat Anti-Rabbit IgG (H+L) #111-035-003
- Peroxidase AffiniPure Goat Anti-Mouse IgG (H+L) #115-035-062

Millipore:
- Anti-Actin Antibody, clone C4 #MAB1501

Modified Anti-EGFR Affibody: Detailed and validated in:
- J Mol Biol. 2008 Mar 7;376(5):1388-402. doi: 10.1016/j.jmb.2007.12.060

	- PLoS One. 2013 May 1;8(5):e62331. doi: 10.1371/journal.pone.0062331
Validation	#3777: Quality control performed by manufacturers.<br>https://media.cellsignal.com/coa/3777/13/3777-lot-13-coa.pdf?__hstc=260778322.b3544842cade1b49267068c6eaa7de15.1533654742618.1533654742618.1537949925467.2&__hssc=260778322.12.1537949925467&__hsfp=1960916031<br><br>#6963: Citations obtained from manufacturer's website.<br>Cooper, J.A. and Howell, B. (1993) Cell 73, 1051-4.<br>Hubbard, S.R. et al. (1994) Nature 372, 746-54.<br>Biscardi, J.S. et al. (1999) J Biol Chem 274, 8335-43.<br>Emlet, D.R. et al. (1997) J Biol Chem 272, 4079-86.<br><br>#2250: Certificate of Analysis from manufacturer<br>https://media.cellsignal.com/coa/2250/10/2250-lot-10-coa.pdf?__hstc=260778322.b3544842cade1b49267068c6eaa7de15.1533654742618.1537954018841.1537957942875.4&__hssc=260778322.5.1537957942875&__hsfp=1960916031<br><br>- #2857. Citation provided by manufacturer<br>Sun, H. et al. (1993) Cell 75, 487-93.<br>Brondello, J.M. et al. (1997) J Biol Chem 272, 1368-76.<br>Franklin, C.C. and Kraft, A.S. (1997) J Biol Chem 272, 16917-23.<br>Li, M. et al. (2003) J Biol Chem 278, 41059-68.<br>Sandberg, E.M. et al. (2004) J Biol Chem 279, 1956-67.<br>Brondello, J.M. et al. (1999) Science 286, 2514-7.<br><br>#352905: Statement from manufacturer's website 'Each lot of this antibody is quality control tested by immunofluorescent staining with flow cytometric analysis'<br><br>#111-035-003: Citation from manufacturer's website<br>Nat Commun. 2018 Aug 15;9(1):3267. doi: 10.1038/s41467-018-05763-8.<br><br>- #115-035-062 Citation provided by manufacturer's website<br>Nat Commun. 2017 Nov 6;8(1):1336. doi: 10.1038/s41467-017-01399-2.<br><br>#MAB1501: Statement and citation from manufacturer's website<br>'Reliably and specifically detect actin using this Anti-Actin Antibody, clone C4. This highly published monoclonal antibody is validated for use in ELISA, IC, IF, IH, IH(P) & WB.' (Otey et al. J Cell Biochem. 1987 Jun;34(2):113-24.)<br><br>Modified Anti-EGFR Affibody: Detailed and validated in:<br>- J Mol Biol. 2008 Mar 7;376(5):1388-402. doi: 10.1016/j.jmb.2007.12.060 (charatisation of binding specificity)<br>- PLoS One. 2013 May 1;8(5):e62331. doi: 10.1371/journal.pone.0062331 (validation of no EGFR activation)

Eukaryotic cell lines

Policy information about cell lines

Cell line source(s)	TR146 cells purchased from the European Collection of Authenticated Cell Cultures (ECACC)
Authentication	Cell lines were not authenticated.
Mycoplasma contamination	Cells were tested negative for mycoplasma contamination
Commonly misidentified lines<br>(See ICLAC register)	NA

Animals and other organisms

Policy information about studies involving animals; ARRIVE guidelines recommended for reporting animal research

Laboratory animals	Mus musculus, Balb/c , female, 11-12 weeks<br>Danio rerio, AB or Tg(mpx:GFP)i114, indeterminate sex, 4 days post fertilisation
Wild animals	NA
Field-collected samples	NA
