# Supplementary material for: Candidalysin activates innate epithelial immune responses via epidermal growth factor receptor
Source: Nat Commun. 2019 May 24;10:2297. doi: 10.1038/s41467-019-09915-2 (PMC6534540; doi:10.1038/s41467-019-09915-2)
Supplement: Supplementary file 2 — Peer Review File [file 41467_2019_9915_MOESM2_ESM.pdf]

Reviewers' comments:

Reviewer #1 (Remarks to the Author):

This is a very well conducted study describing the activation of epidermal growth factor receptor (EGFR) by the Candida albicans secreted peptide toxin, candidalysin (CL). EGFR has been previously described to be hijacked by a variety of pathogens, including C. albicans, to invade mammalian epithelial cells. The authors previously discovered that CL is required for virulence of C. albicans mucosal infections. The toxin is a processed peptide of the ECE1 gene product expressed during hyphal formation which is responsible for epithelial cell invasion resulting in cell lysis and Ca2+ influx.
In this study, they convincingly show that influxed Ca2+ (due to CL lysis of epithelial cells) and EGFR ligands (released by the matrix metalloproteinases [MMPs]) activate the EGFR leading to its phosphorylation and subsequent internalization into the cytosol. The activated EGFR results in release of neutrophil activators such as IL-1β, IL-1α, GM-CSF and G-CSF via the c-Fos and MKP1 pathway. The novelty of the study stems from the description of the role of EFGR in recognition of infection and immunostimulation via a PAMP-independent pathway. Thereby, describing a model by which C. albicans invades the epithelium by binding to EGFR/Her2 tetrodimer via Als3p Candida invasin, while EGFR signaling stimulates the immune system via secretion of PMN-recruiting cytokines. This is an outstanding study that furthers our understanding of the host-pathogen interplay during mucosal candidiasis. I have minor comments.
There needs to be a better explanation to the discordant results seen with the zebrafish and the mouse models. Previous results also show that targeting the EGFR with inhibitors in mice relieves symptoms of OPC which the authors also confirmed. This is not the case in zebrafish. Is it possible that EGFR do not represent a receptor for invasion in the zebrafish model? Alternatively, inhibition of EGFR in zebrafish has been reported to inhibit cardiovascular development (Mech Dev. 2003 Jul;120(7):811-22.). Could there be toxicity issues not realized in uninfected zebrafish?
In Figure 1B. What is the unit for internalization?
Some data are presented as mean + Standard deviation and others are presented as median + interquartile range. There should be uniformity in the data presentation.
Figure 4I ligand, please delete “those not unable…”
There is inconsistency in IL-6 stimulation between Fig 1J (barely any stimulation with CL), and Fig 6D which shows close to 10000 pg/mL stimulation. Is there an explanation? Also, is it true that the difference in Fig 6D is not significant between CL- and B30-treated cells?
Have the authors considered seeing the effect of increased Ca ions on their in vitro system? Would increasing concentrations of Ca without CL, trigger phosphorylation of EGFR?

Reviewer #2 (Remarks to the Author):

Ho et al - Naglik Nature Communications

This is a thorough investigation of the mechanism involved in C. albicans candidalysin (CL) signaling via EGFR, and explores a previously unknown role of this receptor in host defense against Candida mucosal infection. The authors demonstrate that EGFR is required for downstream production of neutrophil chemokines in a CL-dependent manner. However, CL is not a ligand for EGFR, but rather works by increasing shedding of EFGR endogenous ligands (involving MMPs) along with calcium influx (likely the direct effect of CL membrane perturbation. The authors propose that CL-EGFR signaling mediates the immunostimulatory activity of CL, while cytolytic activity mediates cell damage. Whether that immunostimulatory response serves the host vs. the pathogen is dependent on the type of infection and mucosal surface, which needs to be more thoroughly addressed in the discussion, especially since the two models used (immunosuppressed murine OPC vs immunocompentent zebrafish swim bladder) show divergent phenotypes in response to EGFR blockade. Questions below -

Major points:
1. OPC model: PMNs are involved in controlling fungal burden in OPC mice (IL-17 KO and IL-23KO mice administered depleting antibody show increased fungal burden – Gaffen Lab JI 2014). Is the

discrepancy here due to the model using cortisone induced immunosuppression? Cortisone does not render mice neutropenic, but does it prevent neutrophil antifungal defenses? In that case, neutrophils are dispensable and recruitment does not contribute to protection so the role of CL/EGFR in stimulating recruitment in this model is also dispensable. This is evident from previous pubs showing reduced inflammatory infiltrate with the CL mutants but also reduced fungal burden. These issues should be more thoroughly explained in the discussion. In this model the EGFR blockade phenocopied the CL mutant in terms of readouts (reduced CFUs), however, effects on neutrophil recruitment were not shown or discussed. If neutrophils do not play a role in controlling infection, this model really only addresses neutrophil independent roles of CL in pathogenesis.

2. ZF swim bladder model: As this uses immunocompetent fish, neutrophils clearly play a major role in protection as evidenced by increased mortality with EGFR blockage and concomitant reduced neutrophil recruitment. Did this also correlate with increased fungal burden? It isn't clear whether EGFR blockade phenocopies the CL mutants in this model. CL mutants were previously reported to elicit reduced cell damage and reduced neutrophil recruitment – what about CFUs or mortality? It is believed that the cell damage induced by CL overrides the protective effect of PMN recruiting activity? Parallel studies with EGFR blockade with WT and CL mutants would be helpful here along with the neutrophil, CFU, and mortality readouts to make interpretations on the relative role of CL in cell damage vs. PMN recruitment (host defense).

Minor – line 234: refs 4 and 9 appear to refer to studies with oral epithelial cells, not in vivo models – may need to cross-check and update these.

All responses/amendments can be found in blue.

<u>Reviewer #1 (Remarks to the Author):</u>

This is a very well conducted study describing the activation of epidermal growth factor receptor (EGFR) by the Candida albicans secreted peptide toxin, candidalysin (CL). EGFR has been previously described to be hijacked by a variety of pathogens, including C. albicans, to invade mammalian epithelial cells. The authors previously discovered that CL is required for virulence of C. albicans mucosal infections. The toxin is a processed peptide of the ECE1 gene product expressed during hyphal formation which is responsible for epithelial cell invasion resulting in cell lysis and Ca2+ influx.

In this study, they convincingly show that influxed Ca2+ (due to CL lysis of epithelial cells) and EGFR ligands (released by the matrix metalloproteinases [MMPs]) activate the EGFR leading to its phosphorylation and subsequent internalization into the cytosol. The activated EGFR results in release of neutrophil activators such as IL-1β, IL-1α, GM-CSF and G-CSF via the c-Fos and MKP1 pathway. The novelty of the study stems from the description of the role of EFGR in recognition of infection and immunostimulation via a PAMP-independent pathway. Thereby, describing a model by which C. albicans invades the epithelium by binding to EGFR/Her2 tetrodimer via Als3p Candida invasin, while EGFR signaling stimulates the immune system via secretion of PMN-recruiting cytokines. This is an outstanding study that furthers our understanding of the host-pathogen interplay during mucosal candidiasis. I have minor comments.

<u>Response</u>

We thank the reviewer for their kind words.

<u>Reviewer comment 1A</u>

There needs to be a better explanation to the discordant results seen with the zebrafish and the mouse models. Previous results also show that targeting the EGFR with inhibitors in mice relieves symptoms of OPC which the authors also confirmed. This is not the case in zebrafish.

<u>Response</u>

We agree and have amended the text accordingly. See revised manuscript, starting on line 240.

<u>Reviewer comment 1B</u>

Is it possible that EGFR do not represent a receptor for invasion in the zebrafish model?

<u>Response</u>

We have addressed this issue together with the response above (starting on line 240).

<u>Reviewer comment 1C</u>

Alternatively, inhibition of EGFR in zebrafish has been reported to inhibit cardiovascular development (Mech Dev. 2003 Jul;120(7):811-22.). Could there be toxicity issues not realized in uninfected zebrafish?

<u>Response</u>

While we cannot exclude this possibility, we do not believe this to be the case. The reference provided by Reviewer 1 (Goishi et al. 2003) describes a study that uses AG1478 inhibitor immediately post-fertilisation. In our experiments, AG1478 was used starting at 96 hours post-fertilisation and we observed no issues with enlarged hearts, narrowed vessels, heart beat or circulation. Additionally, we saw no mortality in fish treated with inhibitor alone (Fig. 3A). However, we do appreciate the large range of biological functions to which EGFR is known to contribute. Thus,

the use of EGFR inhibitors may have other subtle effects that are not evident in this model. As the data stands, we feel confident that there is no overt toxicity of AG1478 treatment within the context of our study.

Reviewer comment 2
In Figure 1B. What is the unit for internalization?

Response
Values are shown as a Log transformed ratio of the intensity of the inside of the cell to the total cell intensity. This has been amended. See revised manuscript (Fig. 1B).

Reviewer comment 3
Some data are presented as mean + Standard deviation and others are presented as median + interquartile range. There should be uniformity in the data presentation.

Response
Yes, we have done this where possible. However, where the data is not normally distributed, it is necessary to use non-parametric tests and we have presented them as such.

Reviewer comment 4
Figure 4I ligand, please delete "those not unable…"

Response
Amended.

Reviewer comment 5
There is inconsistency in IL-6 stimulation between Fig 1J (barely any stimulation with CL), and Fig 6D which shows close to 10000 pg/mL stimulation. Is there an explanation? Also, is it true that the difference in Fig 6D is not significant between CL- and B30-treated cells?

Response
Yes, we appreciate this comment and Figures 2J, 4M, 5F and 6D indeed show a range of IL-6 concentrations (1000 - 10,000 pg/mL) that were detected following (candidalysin) CL-stimulation. We believe these to be a true reflection of the system, which is of course affected by various factors, such as cell batch and passage number, as these studies were conducted over the course of 3+ years. Additionally, there may be some variation due to different individual researchers who conducted these studies. However, CL is consistently and reliably able to induce IL-6 in all our experiments, which is the main conclusion of these datasets. The reviewer is correct in their final comment, in that no statistical significance was found in Fig. 6D.

Reviewer comment 6
Have the authors considered seeing the effect of increased Ca ions on their in vitro system? Would increasing concentrations of Ca without CL, trigger phosphorylation of EGFR?

Response
We thank the reviewer for this suggestion. Calcium chloride is commonly used to increase calcium availability in cell culture models. This question has actually been addressed in our results as the media used in these studies already contained 1M $CaCl_2$. However, we have now conducted an additional experiment where normal medium was supplemented with $CaCl_2$ to result in total concentrations of 5 mM and 2 mM $CaCl_2$ and applied to cells in the absence of candidalysin (CL). As expected, we found no induction of pEGFR or other readouts of epithelial activation (LDH, c-Fos,

pMKP1) in the presence of additional calcium **(See Fig X1 below (n=1)).** This supports our original theory of EGFR (and epithelial) activation being due to candidalysin-mediated calcium influx (likely through damage/pore formation), as concluded in the primary submission.

Reviewer #2 (Remarks to the Author):

This is a thorough investigation of the mechanism involved in C. albicans candidalysin (CL) signalling via EGFR, and explores a previously unknown role of this receptor in host defense against Candida mucosal infection. The authors demonstrate that EGFR is required for downstream production of neutrophil chemokines in a CL-dependent manner. However, CL is not a ligand for EGFR, but rather works by increasing shedding of EFGR endogenous ligands (involving MMPs) along with calcium influx (likely the direct effect of CL membrane perturbation. The authors propose that CL-EGFR signalling mediates the immunostimulatory activity of CL, while cytolytic activity mediates cell damage. Whether that immunostimulatory response serves the host vs. the pathogen is dependent on the type of infection and mucosal surface, which needs to be more thoroughly addressed in the discussion, especially since the two models used (immunosuppressed murine OPC vs immunocompetent zebrafish swim bladder) show divergent phenotypes in response to EGFR blockade. Questions below -

Reviewer comment 1

OPC model: PMNs are involved in controlling fungal burden in OPC mice (IL-17 KO and IL-23KO mice administered depleting antibody show increased fungal burden – Gaffen Lab JI 2014). Is the discrepancy here due to the model using cortisone induced immunosuppression? Cortisone does not render mice neutropenic, but does it prevent neutrophil antifungal defenses? In that case, neutrophils are dispensable and recruitment does not contribute to protection so the role of CL/EGFR in stimulating recruitment in this model is also dispensable. This is evident from previous pubs showing reduced inflammatory infiltrate with the CL mutants but also reduced fungal burden. These issues should be more thoroughly explained in the discussion. In this model the EGFR blockade phenocopied the CL mutant in terms of readouts (reduced CFUs), however, effects on neutrophil recruitment were not shown or discussed. If neutrophils do not play a role in controlling infection, this model really only addresses neutrophil independent roles of CL in pathogenesis.

Response

We thank Reviewer 2 for their analyses on these points. We appreciate this suggestion and cannot rule this out based on our data, though the use of distinct animal models may also play a large role here. Importantly, in the event that these discrepancies were indeed due to cortisone usage, it would not question the role of neutrophils in protection, but rather in fungal load. Neutrophils have long been considered to have relatively one-dimensional functions, existing solely for pathogen killing. More recently, however, additional functions have come to light which describe the ability of neutrophils to help guide the course of inflammatory responses through non-killing and non-damaging actions, including wound repair and even anti-inflammation. We believe our data obtained in zebrafish, showing an unchanged fungal load despite reduced neutrophil recruitment, may provide supportive evidence of such neutrophil functions and have now included a paragraph with references within the main body of text to discuss this (starting on line 250). Thus, rather than these discrepancies in fungal load being attributed to cortisone use, we propose that a combination of distinct animal models and emerging neutrophil functions may contribute, highlighting a potential role for neutrophils in controlling the pathogenic environment of the infection site rather than the pathogen directly.

Reviewer comment 2

ZF swim bladder model: As this uses immunocompetent fish, neutrophils clearly play a major role in protection as evidenced by increased mortality with EGFR blockage and concomitant reduced neutrophil recruitment. (i) Did this also correlate with increased fungal burden? It isn't clear whether EGFR blockade phenocopies the CL mutants in this model. CL mutants were previously reported to elicit reduced cell damage and reduced neutrophil recruitment – what about (ii) CFUs or (iii) mortality? (iv) It is believed that the cell damage induced by CL overrides the protective effect of PMN recruiting activity? Parallel studies with EGFR blockade with WT and CL mutants would be helpful here along with the neutrophil, CFU, and mortality readouts to make interpretations on the relative role of CL in cell damage vs. PMN recruitment (host defense).

Response

The reviewer touches on several interesting and complex issues, which we have numbered for clarity. Firstly, the reviewer is correct, our data suggests that neutrophils play a major role in protection during *C. albicans* infection, which may not be through traditional killing actions. This is highlighted by an unchanged fungal load despite reduced neutrophil recruitment (i). The data supports our previous work showing that CL-mutants do not exhibit deficient fungal loads (ii) *(Richardson et al. 2017)*, and as CL signals through EGFR, it is reasonable that we observe a similar effect in EGFR inhibited animals (as shown in the current study). Unfortunately, previous studies using CL-mutants were conducted in mice which do not mortally succumb to infection, so this was not a possible readout (iii). Interestingly, we have shown that CL is able to induce both cell damage and protective neutrophil functions. The question of whether and under what conditions cell damage will 'win out' over neutrophil protection (iv), is a complex and multifactorial matter, which we agree is very interesting. This aspect would require a thorough investigation however and may not be within the scope of our study which primarily aims to provide evidence for the importance of EGFR in governing protective host responses to CL. We have, however, now provided comment on this issue within the revised manuscript (starting line 261).

Reviewer comment 3

Minor – line 234: refs 4 and 9 appear to refer to studies with oral epithelial cells, not in vivo models – may need to cross-check and update these.

Response

Ref 4 is *in vitro* whilst ref 9 is *in vivo* but we have added an additional reference for support (Line 235).

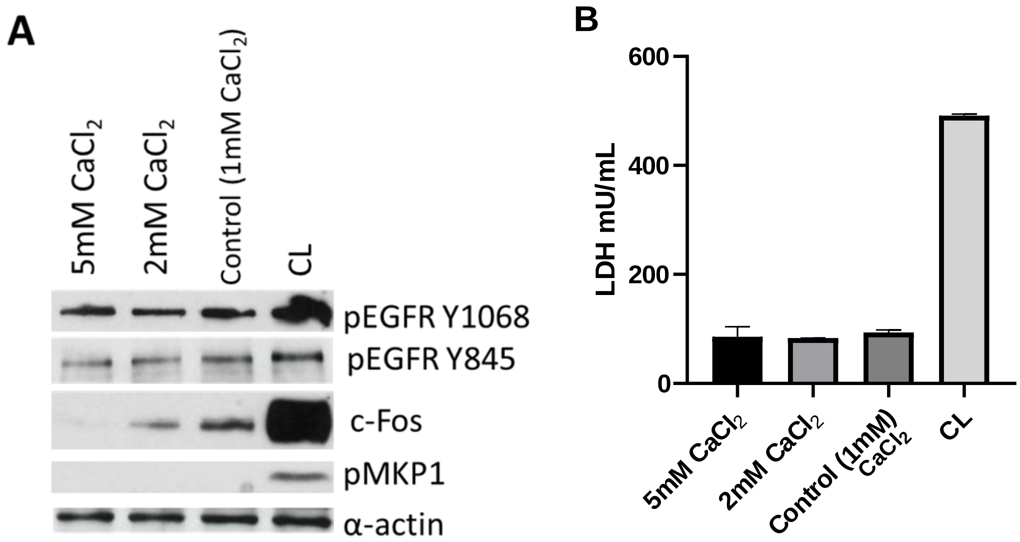

Fig. X1: Addition of exogenous calcium chloride does not mimic CL function. TR146 cells were cultured in media containing 5mM $CaCl_2$, 2mM $CaCl_2$, normal medium (1mM $CaCl_2$) or 70 µM CL. The presence of additional available Ca^{2+} did not induce pEGFR (Y1068 or Y845), c-Fos, pMKP1 (A) or LDH release (B).

REVIEWERS' COMMENTS:

Reviewer #1 (Remarks to the Author):

I have no further comments. The authors have satisfactorily answered the critiques.

Ashraf Ibrahim
